# Deconvoluting the Convoluted: Structural Ramifications of Postsynthetic Linkage Modification in COF-366-Co Catalysts

**DOI:** 10.1021/acs.chemmater.6c01374

**Published:** 2026-10-07

**Authors:** Samuel Van Gele, Amelie Heilmaier, Fabian Heck, Kathrin Küster, Armin Schulz, Artem Shevchenko, Liang Yao, Bettina V. Lotsch

**Affiliations:** † 28326 Max Planck Institute for Solid State Research, Heisenbergstr. 1, 70569 Stuttgart, Germany; ‡ Department of Chemistry, Ludwig-Maximilians-Universität (LMU), Butenandtstr. 5-13, 81377 Munich, Germany; § Collaborative Research Center 1333, Universität Stuttgart, Pfaffenwaldring 55, 70569 Stuttgart, Germany; ∥ 26467 South China University of Technology, State Key Laboratory of Luminescent Materials and Devices, 510640 Guangzhou, China

## Abstract

Postsynthetic modification (PSM) is a powerful strategy for modifying properties such as pore environment, stability and catalytic activity in covalent organic frameworks (COFs). While the chemical outcomes of PSMs are well documented, their impact on the underlying crystal lattice is rarely quantified. Yet, when translating modified COF systems toward catalytic applications, such structural changes can impact performance. In this work we interrogate precisely that blind spot for the benchmark electrocatalyst COF-366-Co. We apply three chemical PSM routes to the imine backbone: (i) quinoline cyclization (q-COF-366-Co), (ii) amine reduction (r-COF-366-Co), and (iii) nitrone oxidation (o-COF-366-Co) and investigate chemical as well as structural modifications induced by the functionalization. The catalysts are further investigated toward the electrochemical carbon dioxide reduction reaction in an attempt to decouple the multitude of convoluted aspects that affect catalytic activity, stability and selectivity such as particle size, disorder, strain, catalytic environment, and framework stability. Among the series, q-COF-366-Co showed a higher stability toward CO selectivity over time compared to the pristine COF-366-Co at −0.88 V vs reversible hydrogen electrode (RHE). Investigating q-COF-366-Co further showed, however, that higher functionalization concentrations led to simultaneous increase in disorder and a lower selectivity toward CO during catalysis. This indicates that a balance must be found between functionalization and preservation of crystalline lattice, offering insights into the design importance of maintaining structural coherence when comparing COF-based materials toward applications.

## Graphical abstract

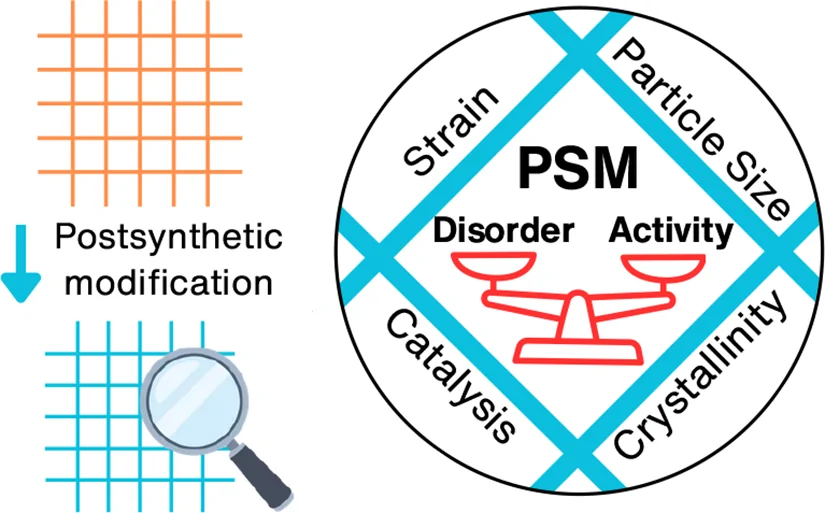

## Introduction

Postsynthetic modifications (PSMs) are widely employed to tune the functionality of covalent organic frameworks (COFs), yet their structural consequences are often insufficiently characterized, limiting the ability to compare materials as well as establish meaningful structure–property relationships.^1,2^ This challenge is particularly relevant in the broader context of reticular materials, where precise structural control is central to function. COFs, with their crystalline order and modular porosity, offer a versatile platform for designing functional materials for a wide range of applications spanning from separation and drug delivery, to catalysis and energy storage.^3–6^ Their tunable architectures make them especially attractive for heterogeneous catalysis, where PSM can serve as a key strategy to introduce catalytic functionality or modulate local environments without disrupting the overall framework. Since the seminal report by Yaghi et al. demonstrating that the imine-based COF-366-Co outperforms its molecular analogue in the aqueous electrocatalytic CO_2_ reduction reaction (CO_2_RR), COFs have attracted intense interest in electrocatalysis.^7–9^ Many research efforts have focused on incorporating diverse catalytic linkers, such as metallic porphyrins and phthalocyanines, and on embedding additional catalytic functionalities within the preformed COF scaffold.^9–12^ Building on this foundation, researchers have subsequently turned to structural tuning strategies to further boost COF-366-Co performance, for example by expanding the pore dimensions through longer linkers or by altering the electronic environment via reticular linker modification.^13–15^

Even though being interesting catalysts toward CO_2_RR, many imine-based COFs suffer from stability issues over time. Some porphyrin and phthalocyanine systems have been shown to exhibit long-term electrocatalytic stability over many hours, yet most porphyrin-based systems show low stability toward CO production, especially at more negative potentials.^10^ To overcome this limitation, researchers have turned to various complementary routes to enhance framework as well as catalyst stability by (i) strengthening the COF backbone by employing chemically robust linkages (β-ketoenamine, dioxin, vinylene, etc.),^12,16–23^ (ii) strengthening the backbone by employing postsynthetic modifications,^24–27^ (iii) utilizing donor–acceptor type linker combinations to facilitate electron transfer,^13^ and (iv) introducing mixed-metal centers that can share the catalytic load and work synergistically.^28^ These approaches are crucial for COFs, as it is necessary to address these stability challenges by developing robust frameworks to harness their full potential and implement them in larger scale operations which can withstand industry-relevant conditions without significant degradation.^29^

Nevertheless, the local chemistry surrounding the catalytic center is not the sole factor influencing catalytic stability and activity. Our group has previously demonstrated that particle-size engineering is a decisive lever for unlocking the intrinsic activity of COF electrocatalysts, independent of the underlying framework chemistry.^30^ Indeed, smaller particles expose more catalytic sites, which improves COF-electrode interactions. Thus, as the catalytic performance of COFs is tightly coupled to particle size and morphology, any chemical-modification protocol must strive to leave the material overall untouched, otherwise the observed activity changes could simply reflect altered morphology rather than true framework structure–property effects. Maintaining these structural features is essential for a fair, head-to-head comparison between pristine COF-366-Co and its functionalized counterparts. For instance, reductions with NaBH_4_ have been employed to convert Co-porphyrin based COF imine linkages to amine bonds and further introduce pendant functional groups onto COF backbones, but in the case of COF-366-Co, such treatments have led to a loss of crystallinity.^24,25^ This degradation makes it difficult to determine whether observed changes in catalytic activity stem from the intended chemical modification or from inadvertent alterations in sheet stacking, amorphization, particle size, or site accessibility.

Herein we translate three postsynthetic modification strategies to COF-366-Co to obtain a quinoline-linked (q-COF-366-Co), amine-linked (r-COF-366-Co), and nitrone-linked (o-COF-366-Co) COF. These PSMs were selected in an attempt to simultaneously improve COF stability toward electrocatalysis as well as tune local hydrophilicity. The PSMs were obtained all the while attempting to preserve the crystalline and nanoscale structure, to investigate linkage stability, as well as local environment effects toward CO_2_ electrocatalysis, deconvoluted from structural aspects. We observed that PSM reaction conditions (i.e., functionalization concentrations) do influence the pristine structure of COFs. In the case of q-COF-366-Co, light PSM concentrations offer a modest increase in activity whereas a large PSM concentration leads to disorder-induced deactivation, offering insights into the effect of material order on electrocatalysis.

## Experimental Section

### Materials and Equipment

#### General Methods

Reaction temperatures were electronically monitored with a probe in the oil bath. Reagents were purchased from different commercial sources and used without further purification.

#### Gas Sorption Measurements

N_2_ sorption isotherms were recorded on a Quantachrome Instruments Autosorb iQ 3 sorptometer at 77 K. BET analyses were conducted using a program written in our group by Alexander M. Pütz (https://github.com/AlexanderPuetz) based on the software BETSI by Osterrieth and Fairen-Jimenez.^2^ The fitting ranges were selected to meet all four Roquerol criteria and to minimize the error under the fourth Rouquerol criterium, i.e., between the experimental and theoretical partial monolayer loading. The cutoff value for *R* ^2^ was set at 0.99 to obtain a satisfactory number of valid fitting ranges. Valid ranges were further limited to those where the maximum partial pressure was equal to the partial pressure of the highest value of the Rouquerol representation. The pore size distributions (PSD) were calculated with a model of N_2_ at 77 K on carbon using the quenched solid density functional theory (QSDFT) (cylindrical pores, adsorption branch) in AsiQwin software v3.01. The samples were activated under a high vacuum at 120 °C for 12 h before measurements.

#### Powder X-ray Diffraction (PXRD)

Powder X-ray diffraction experiments were performed on a Stoe Stadi P diffractometer (Cu–Kα_1_, monochromatized with Ge(111)) in Debye–Scherrer geometry. The samples were measured in sealed glass capillaries (OD = 1.0 mm) and spun for improved particle statistics. fwhm analysis of the 100 peak was performed by first deconvoluting the 100 and 110 peak as their broadness in the PXRD causes them to overlap. The deconvolution was performed with TOPAS v7. The background was corrected with Chebychev polynomials (Order 5). Peaks were deconvoluted utilizing PV functions in the range of 2θ = 2.2–10°. The range was chosen to ensure correct PXRD baseline detection. COF-366-Co as well as the 1:0.5, 1:1.5, 1:2.5 were all compared within three different batches. 1:0.1, 1:1, and 1:2 variants were synthesized once to check observed trend. Reported error is the fitting error of fwhm in TOPAS v7 from 3 replicates. Pawley refinements were performed between 2θ = 2.6–40° with TOPAS v7 utilizing a simple axial model, zero-error peak shift, Thompson-Cox-Hastings pseudo-Voigt peak shapes, and Lorentzian crystallite size and strain approximations. The background was corrected with Chebychev polynomials (Order 5).

#### Raman Spectroscopy

Raman spectra of synthesized covalent organic frameworks were recorded using a Jobin Yvon Typ V 010 LabRAM single grating spectrometer, equipped with a double super razor edge filter and a Peltier-cooled charge-coupled device camera. The resolution of the spectrometer (grating 1800 lines/mm) was 1 cm^–1^. The spectra were taken in a quasi-backscattering geometry using the linearly polarized 632.817 nm line of a He/Ne gas laser, 532.0 nm line and 456.9 nm lines of diode lasers. The power was lower than 1 mW in order to avoid local heating and have been verified and reduced for every individual sample depending from the incident laser line to be safe against any influence from laser light: 0.04 mW/532.0 nm line, 0.1 mW/456.9 nm line, 0.2 mW/632.817 nm line. The spot size was 10 μm, focused by a 50x microscope objective on to the surface of the sample.

Every sample have been checked by Raman microscope objective picture before and after each measurement for stability against possible conversion by laser light. (No optical difference by taken pictures).

#### Supercritical CO_2_ Activation

Activation of the methanol-soaked COF samples with supercritical CO_2_ was performed on a Leica EM CPD300 critical point dryer. Solvent removal from the COFs was performed via supercritical CO_2_ extraction using a Tousimis Autosamdri-815 Series B Critical Point Dryer. The sample were previously washed and suspended in methanol.

#### Scanning Electron Microscopy (SEM)

SEM SE (secondary electron) detector images were obtained on a Zeiss Merlin SEM.

#### Transmission Electron Microscopy (TEM)

TEM analysis was performed with a Philips CM30 ST (300 kV, LaB6 cathode). The samples were prepared dry onto a copper lacey carbon grid (Plano).

#### Inductively Coupled Plasma Optical Emission Spectroscopy (ICP-OES)

ICP-OES measurements were conducted on a Varian Vista-PRO spectrometer. The samples were digested with a CEM Discover SP-D microwave reactor using 65% HNO_3_ aq at 185 °C for 25 min and were diluted with water.

#### Infrared (IR) Absorbance Spectroscopy

IR absorbance spectra were recorded on a PerkinElmer Spectrum Two spectrometer in attenuated total reflection (ATR) geometry equipped with a diamond crystal. The measurements were conducted with a resolution of 1 cm^–1^. The spectra were corrected for the ATR effect (contact parameter: 0), subtracted with baselines, and normalized at νC=C peaks at 1600 cm^–1^.

#### Solid-State Nuclear Magnetic Resonance (ssNMR) Spectroscopy

^13^C cross-polarization (CP) and ^19^F magic-angle spinning (MAS) ssNMR spectra were recorded on a Bruker Avance III 400 MHz spectrometer at a rotation frequency of 12500 Hz, a temperature of 293 K, and a contact time (p15) of 1 ms, and chemical shifts (δ) were referenced relative to α-glycine (176.5 ppm).

#### Water Contact Angle Measurement (WCA)

Water contact angle measurements were performed by dropping a 3 μL drop of milli-Q water onto the investigated substrate. Droplet sphere approximation and WCA angle analysis were performed with ImageJ and the Contact Angle plugin (Author: Marco Brugnara).^31^ WCA on the electrodes were measured precatalysis. In measurements on electrodes, three water droplets were deposited and analyzed for statistical purposes. The blank electrode consists of an electrode prepared in a typical manner but omitting the COF catalyst from the ink.

#### X-ray Photoelectron Spectroscopy (XPS)

X-ray photoelectron spectroscopy (XPS) measurements were performed using a Kratos Axis Ultra spectrometer equipped with a monochromated Al Kα source (hν = 1486.7 eV). The samples were prepared by pressing the powders into indium foil. A charge neutralizer was used to suppress surface charging and the binding energy of the C 1s was set to 284.8 eV for calibration.^32^ Survey spectra were acquired with a pass energy (PE) of 80 eV, while high-resolution detail scans were recorded at a PE of 20 eV to ensure accurate peak analysis. For quantification the XPS peak areas were corrected for photoionization cross sections and analyzer transmission using CasaXPS.^33^

### Synthesis

#### Co­(tdpp)

Co­(tdpp) (([5,10,15,20−tetrakis­(4−(4,4’’−Dimethoxytritylamino)­phenyl)­porphinato]­cobalt (II)) was synthesized similar to a method based on a previous report in our group.^30^ A 250 mL two-neck Schlenk flask with a magnetic stir bar, a reflux condenser, a rubber septum, and a paraffin bubbler was dried under vacuum with a heat gun and charged with TAPP (501 mg, 742 μmol, 1 equiv) and anh. THF (120 mL) under Ar. The mixture was cooled at 0 °C. Et_3_N (1.24 mL, 12 equiv) and TrBr (1.20 g, 5 equiv) were added to the mixture. The mixture was stirred at room temperature under the dark for 1 h. Et_3_N (2.58 mL, 25 equiv) and anhydrous CoCl_2_ (481 mg, 5 equiv) were added to the mixture. The setup was briefly purged with Ar and the mixture was bubbled with Ar for 30 min. The mixture was heated to reflux under 1.25 atm for 2 h, cooled to RT, and chromatographed through a pad of neutral Al_2_O_3_ (45 g) with THF until eluate became pale yellow. The eluate was dried by rotary evaporator and then under vacuum. The solids were dissolved in CHCl_3_ (75 mL). Reprecipitation was induced with *n*-hexane (375 mL). The precipitate was collected by suction filtration, rinsed with CHCl_3_/*n*-hexane (1:10, v/v), and dried under vacuum to yield Co­(tdpp) as red solids (73% yield).

#### COF-366-Co

COF-366-Co was synthesized similarly to a previous report by our group.^30^ An 8 mL Schlenk bomb was charged with Co­(tdpp) (80.9 mg, 41.07 μmol, 1 equiv), water (45.0 equiv), and previously prepared and sonicated PhNO_2_/DO (1:3, v/v) solution of TPA (11 mg, 82.1 μmol, 2 eq., 667 μL). The mixture was sonicated. BzOH (150 mg, 1232 μmol, 30 equiv) was added to the mixture, and the mixture was immediately frozen with a liquid N_2_ bath, the inner atmosphere was evacuated at <0.01 mbar, the tap was closed, and the mixture was thawed with a water bath at RT. This freeze–pump–thaw cycle was repeated two more times. The mixture was manually stirred, sonicated, heated at 120 °C in a convection oven for 3 days, and cooled to RT. DMF (1 mL) was added to the mixture. The solids were collected to a filter paper by suction filtration and rinsed with DMF, CHCl_3_, and MeOH while keeping the solids wet with solvents. The filter paper containing the solids was washed with Soxhlet extraction in MeOH overnight and further rinsed and activated with scCO_2_, to give COF-366-Co as dark purple solids (32 mg, 83% yield).

#### Quinoline Linkage Modification – q-COF-366-Co

q-COF-366-Co (= q-COF-366-Co 1:0.5) was synthesized similar to a previously reported method by Li et al.^34^ COF-366-Co (30 mg, 0.0323 mmol, 1 equiv) (established from COF unit cell)) and chloranil (11.9 mg, 0.049 mmol, 0.65 equiv) were added into a reaction vessel and purged with argon. Toluene (10 mL), 1-ethynyl-4-(trifluoromethyl)­benzene (10.6 μL, 0.065 mmol, 2 equiv) and boron trifluoride-diethyl ether (8.19 μL, 0.065 mmol, 2 equiv) were then added to the flask in sequence. The reaction mixture was heated at 120 °C for 1 day under stirring. The crude product (black powder) was washed with acetone, dimethylformamide, chloroform and methanol, purified via Soxhlet extraction with methanol overnight. The sample was dried with supercritical carbon dioxide to afford a dark colored powder of q-COF-366-Co (24.7 mg)

For the series of functionalization concentrations, the amounts of reactants were adjusted as described in Supplementary Table S1.1.1.

#### Amine Linkage Modification – r-COF-366-Co

r-COF-366-Co was synthesized with a method adapted on a previous report by our group.^35^ Under argon, COF-366-Co (30 mg, 0.0323 mmol, 1.00 equiv) was dispersed in a mixture of PhNO_2_/DO (1:3, 3 mL) in a microwave vial and sonicated. Formic acid (97%, 35.2 μL, 0.905 mmol, 28.0 equiv). was added. The vial was sealed and stirred at 200 rpm at 120 °C for 48 h. The crude product was washed with DMF, CHCl_3_, and methanol, purified via Soxhlet extraction with methanol overnight, and dried with supercritical carbon dioxide to afford a dark-reddish powder of r-COF-366-Co (27.4 mg).

For the series of functionalization concentrations, the amounts of reactants were adjusted as described in Supplementary Table S1.1.2.

#### Nitrone Linkage Modification – o-COF-366-Co

COF-366-Co (30 mg, 0.0323 mmol, 1.00 equiv) was dispersed in DCM (1.5 mL) in a microwave vial and cooled to 0 °C with an ice bath while stirred at 200 rpm. Next, mCPBA (22.8 mg, 0.129 mmol, 4.00 equiv) was dissolved in DCM (2.55 mL) and added dropwise to the COF mixture. The vial was sealed and stirred at 200 rpm for 18 h, while the temperature was allowed to warm up to RT. The crude product was washed with DMF, and methanol, purified via Soxhlet extraction, and dried with supercritical carbon dioxide to afford a dark-purplish powder of o-COF-366-Co (28.5 mg).

For the series of functionalization concentrations, the amounts of reactants were adjusted as described in Supplementary Table S1.1.3.

### Electrochemical Experiments

#### Electrode Preparation

Catalyst inks are prepared by mixing COF catalyst (ca. 1 mg), carbon black (Vulcan XC 72R, 2 mg/mg to COF), Nafion solution (1%, 5 μL/mg to COF), and anhydrous EtOH (500 μL/mg to COF) and sonicating for at least 30 min. 5% polytetrafluoroethylene (PTFE)-coated carbon paper (CP, Quintech TP-060-T5) was cut into a paddle shape with a catalyst-coating area (ca. 0.65 cm × 1 cm) and a noncoating area (ca. 0.4 cm × 1 cm). The electrodes were washed by sonication in ethanol (10 min) twice, followed by drying with compressed air. The catalyst ink was sonicated again and drop-cast onto the catalyst-coating areas of the carbon paper on both sides with 30 μL each (COF loading amount: 0.12 mg). The drop-casted CP pieces were dried under a vacuum and were connected to copper wires by sandwiching the uncoated areas and the copper wires with titanium foils and a custom-made polyetheretherketone (PEEK) electrode holder.

#### Electrochemistry Setup

Electrochemical measurements and electrolysis were carried out with a Pine Research WaveDriver 200 EIS Bipotentiostat and a custom-designed gastight two-compartment H-cell. The H-cell is optimized in volume, inert and gastight connections, compatibility with high-purity gas handling and instrumentation standards, modularity, and leakage-proof. Anion exchange membrane (Fumatech fumasep FAB-PK-130) was pretreated sequentially with 0.5 M NaCl aq., 0.5 M KOH aq., and 0.5 M KHCO_3_ aq., and then was assembled inside the H-cell. A three-electrode configuration was employed, with a Ag/AgCl electrode (porous glass/sat. KCl aq./AgCl/Ag) as a reference electrode and a Pt mesh as a counter electrode. The working compartment of the H-cell was equipped with a reference electrode, a working electrode, a stir bar, a CO_2_ inlet tube, and a gas outlet tube. The counter compartment was equipped with a counter electrode and a leak valve. Both compartments were charged with 0.5 M KHCO_3_ aq. electrolyte (8.5 and 6 mL for the working and the counter compartment, respectively). The working compartment was subjected to a constant bubbling flow of CO_2_ (20 mL/min, referenced to volume at 0 °C) controlled by a Bronkhorst High-Tech F-201DV mass flow controller under magnetic stirring and the control of a back pressure controller (1150 mbar). Before performing any electrochemical experiment, the electrolyte was bubbled with CO_2_ for 10 min to saturate it with CO_2_ and to remove oxygen in the system, and the working electrodes were activated by cathodic scan cycles (0 V to −0.98 V vs RHE, 20 mV/s, 10 times). The electrolyte pH was 7.3 after being saturated with CO_2_. All experiments were carried out at ambient temperature. Cyclic voltammograms were recorded with a scan rate of 5 mV/s.

Electrode potentials were converted to the reversible hydrogen electrode (RHE) scale using *E*_RHE_ = *E*_Ag/AgCl_ + 0.059 × pH + 0.197 without *iR* compensation.

#### Gaseous Product Quantification

Gas quantification is conducted in a setup similar to that reported in previous work.^3^ Outflow gas was introduced into an autosampler of a Shimadzu Nexus GC-2030 gas chromatograph (GC) by intervals of 11.2 min, chromatographed with helium (99.999%, Air Liquide) as a carrier gas, and detected with a dielectric-barrier discharge ionization detector (BID). The low headspace volume of the H-cell allows a steady-state response at the reactor outlet in the order of 1 min. The GC was calibrated with standard calibration gas mixtures containing CO, H_2_, and CH_4_ purchased from Air Liquide (4-point linear calibration, *R* ^2^ > 0.999).

#### Liquid Product Detection

A portion of the electrolyte after electrolysis was diluted with D_2_O containing DMSO as an internal standard and subjected to solution ^1^H NMR measurement. No signal of formate or other species was detected. Assuming the lower detection limit to be a signal-to-noise ratio of 3, the faradaic efficiencies for liquid products are negligible (faradaic efficiency <1%).

#### Catalysis Parameter Calculation

The faradaic efficiency (FE) for each product was calculated using the following equation:

$$\mathrm{FE}=\frac{\mathrm{xPV}}{RT_{r}}\cdot \frac{\upsilon F}{I}$$

where *x* is the mole fraction of the product in the outflow gas measured by the GC, *P* is the pressure (1150 mbar) controlled by the back pressure controller, *V* is the volumetric gas flow rate referenced to volume at 0 °C controlled by the mass flow controller (20 mL min^–1^), *R* is the ideal gas constant, *T*_r_ is the reference temperature (0 °C), υ is the number of electrons transferred (2 for CO and H_2_), *F* is the Faraday constant, and *I* is the total current measured by the potentiostat.

## Results

### Postsynthetic Modification of COF-366-Co

COF-366-Co was synthesized using an in situ deprotection method by condensing Co­(tdpp) ([5,10,15,20-tetrakis­(4-(4,4’-Dimethoxytritylamino)­phenyl)­porphinato]­cobalt­(II)) and terephtaldehyde (4,4’-phenyldicarbaldehyde) under solvothermal conditions for 72 h at 120 °C, similar to a previous report by our group.^30^ Subsequently, the framework underwent PSM to form three distinct linkages, with synthetic conditions optimized to preserve crystallinity and structure (Figure 1). The quinoline-linked framework (q-COF-366-Co) was achieved through imine cyclization via the Povarov reaction with 1-Ethynyl-4-(trifluoromethyl)-benzene to simultaneously cyclize and introduce trifluoromethyl functionalization into the pore in conditions optimized from Li and co-workers.^34^ The amine-linked variant, r-COF-366-Co (r for reduced), was obtained via a Leuckart–Wallach reduction, reacting COF-366-Co with formic acid under conditions previously described by our group.^35^ Lastly, the nitrone-linked form, o-COF-366-Co (o for oxidized), was obtained through an oxidation process using meta-chloroperoxybenzoic acid (mCPBA) as the oxidizing agent.^36^

**Figure 1:**
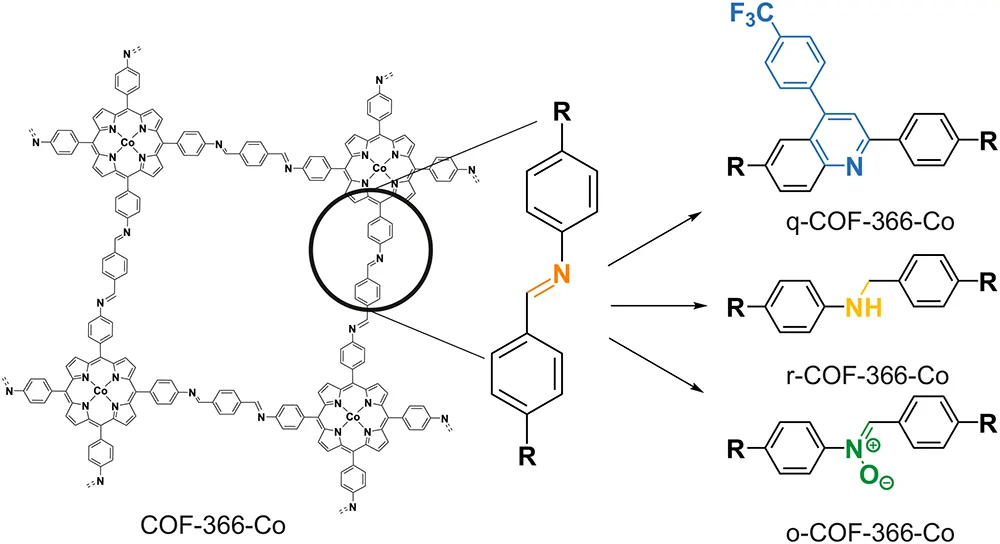
Postsynthetic functionalizations of imine-linked COF-366-Co forming quinoline-linked q-COF-366-Co, amine-linked r-COF-366-Co, and nitrone-linked o-COF-366-Co.

Powder X-ray diffraction (PXRD) analysis showed that all materials exhibited four reflections at 2θ = 3.5°, 4.1°, 7.0°, and 8.3°, corresponding to the 100, 010, 200, and 020 reflection planes, respectively. Additionally, all diffractograms exhibited identical full-width at half-maximum (fwhm) of the 100 peak (0.29 ± 0.02° for all samples), confirming the preservation of crystallinity after PSM, crucial for deriving structure–property relationships (Figure 2a). Pawley refinements additionally confirmed the retention of the unit cell parameters after functionalization, notably being identical to that of the slipped stacking previously reported for COF-366-Co (S2.1, Supplementary Figures S2.1.6–8).^30,37,38^

**Figure 2:**
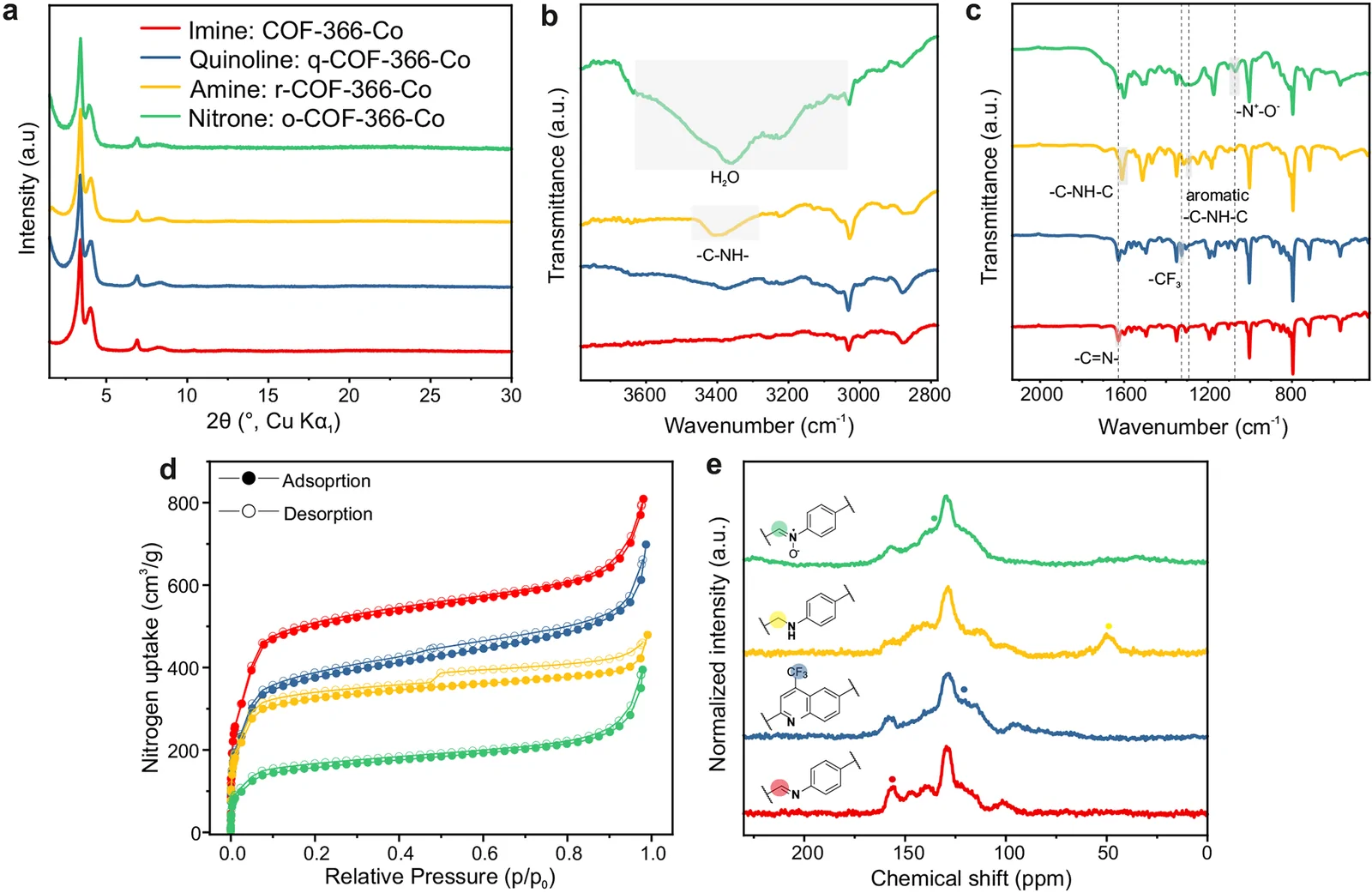
Normalized PXRD (a), normalized FTIR spectrum (b, c), nitrogen sorption (d), and ^13^C ssNMR (e) of COF-366-Co (red), q-COF-366-Co (blue), r-COF-366-Co (yellow), o-COF-366-Co (green).

Fourier-transform infrared spectroscopy (FTIR) analysis revealed successful functionalization of COF-366-Co in all three instances. Successful Povarov cyclization was indicated by the appearance of stretching bands at 1323 cm^–1^ and attributable to (νC–F_3_) for q-COF-366-Co. For r-COF-366-Co, three new vibrations assignable to secondary aromatic amines (νN–H) at 3405 cm^–1^, 1610 cm^–1^, and 1290 cm^–1^ appeared, while the intensity of the imine vibration (νN=C) at 1626 cm^–1^ disappeared, supporting the successful formation of the amine linkage. For o-COF-366-Co, the presence of a stretching band at 1070 cm^–1^ attributed to the nitrone (νN–O), the absence of an amide stretch, as well as a large broad signal between 3100 cm^–1^ and 3600 cm^–1^ concurrent with that of water indicate that the oxidation led to the formation of a nitrone, similar to previous reports (Figure 2b,c).^36,39^

Nitrogen gas sorption experiments confirmed that the postfunctionalized catalysts remained porous, with overall nitrogen uptake being, however, lower for all postfunctionalized samples compared to that of the pristine COF (Figure 2d). Calculated Brunauer-Emmet-Teller (BET) surface areas decreased from 2194 m^2^/g for COF-366-Co, to 1469 m^2^/g, 1294 m^2^/g, and 580 m^2^/g for q-, r-, and o-COF-366-Co, respectively (S2.3, Supplementary Figures S2.3.1–4). The pore sizes also reduced from being centered around 2.17 nm for COF-366-Co to 2.13 nm, 2.08 and 2.15 nm for q-, r-, and o-COF-366-Co, respectively (S2.3, Supplementary Figures S2.3.8). The decrease in uptake capacity and the reduced surface area provides the first evidence that PSM does not leave the framework unchanged and, instead readily alters the intrinsic porosity of the pristine material and by proxy the mass transport which may affect catalysis.^40^

To elucidate the local chemical environment, ^13^C cross-polarization magic-angle spinning solid-state NMR (ssNMR) experiments were conducted. Due to the inherent disorder in COFs as well as in this case, the paramagnetic Co^II^ center, ssNMR offered rather broad signals. Nevertheless, the successful postfunctionalization of q-COF-366-Co and r-COF-366-Co was identified, in the first case, by the broadening of the signal at 130 ppm and by the appearance a shoulder-like feature at 123 ppm that appears in the region expected of a broad signal for an aromatically linked −CF_3_ carbon and, in the second case by a characteristic amine carbon at 49.7 ppm for r-COF-366-Co (Figure 2e).^41^ In the case of o-COF-366-Co, elucidating the chemical environment via solid-state NMR proved challenging, as the expected nitrone signal, previously reported at approximately 135 ppm, overlaps with broader aromatic resonances from the COF (Figure 2e).^39^

The morphology of the pristine and postfunctionalized COFs was investigated by both scanning electron microscopy (SEM) and transmission electron microscopy (TEM). The SEM images reveal that all postsynthetically modified materials had similar morphology to that of pristine COF-366-Co, showing agglomerated polycrystalline particles with sizes up to several microns, with o-COF-366- showing smaller aggregates consistent across different SEM images (Figure 3a-d, right). This modification must be taken note of, as particle size and morphology readily influence catalysis.^30^ TEM images, however, suggest that the nanoscale environment remained identical pre- and postfunctionalization, with the COF clearly visible in each material, all showing two identical d-values for all materials of 24 Å and 20 Å, as obtained by Fast Fourier Transform (FFT), consistent with 100 and 010 lattice planes. (Figure 3a-d, left).

**Figure 3:**
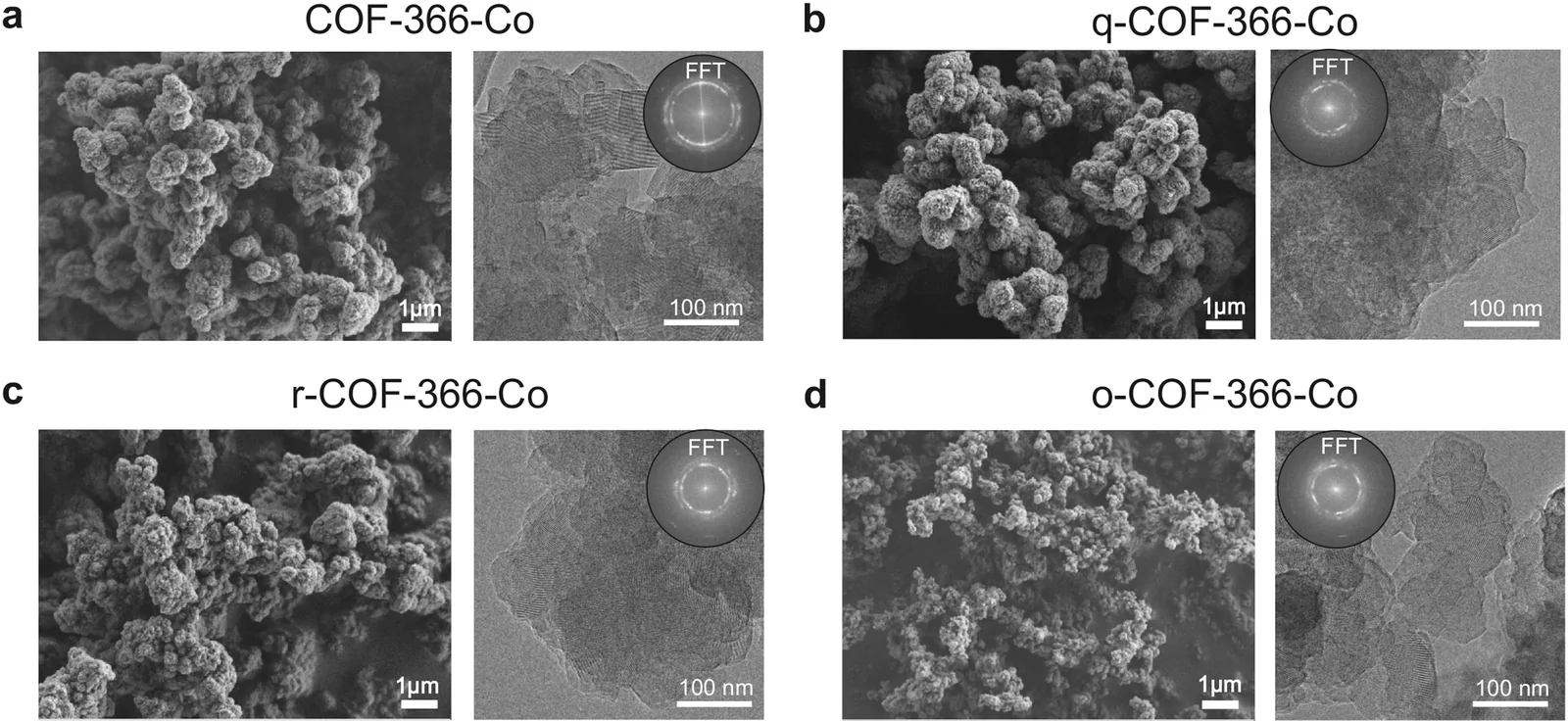
SEM (left) and TEM (right) images of COF-366-Co (a), q-COF-366-Co (b), r-COF-366-Co (c), and o-COF-366-Co (d).

All in all, the three postsynthetic transformations successfully introduced the targeted functionality while preserving the COF’s crystalline framework. Even though no increase in the fwhm of the 100 peak was observed with the utilized reaction conditions, the lower nitrogen uptake in sorption measurements and slightly smaller particle sizes in the case of o-COF-366-Co do hint that there are shortcomings in finding optimal postsynthetic conditions that embed new chemistry but leave the structure of the pristine material untouched. However, compared to previously reported PSM methodologies, the materials in this study demonstrate that functionalization and chemical modification of COFs can be achieved without sacrificing crystallinity, which is a crucial prerequisite for further investigating structure–property relationships and deconvoluting aspects influencing catalysis.

### Electrochemical CO_2_ Reduction

To investigate the influence of functionalization toward electrochemical CO_2_RR, the catalysts, COF-366-Co, q-COF-366-Co, r-COF-366-Co, and o-COF-366-Co, were compared in terms of stability and selectivity toward CO formation during electrochemical CO_2_ reduction in 0.5 M aqueous KHCO_3_ solution under CO_2_ flow in an H-Cell setup (see Experimental Section). The working electrodes were prepared with a COF sample as a catalyst, carbon black as a conducting agent, and Nafion as a binder on carbon paper electrodes. Cyclic voltammetry (CV) of the electrodes shows catalytic currents at reductive potentials. The onset, determined as the potential at a current density of −1 mA/cm^2^, was −0.58 V vs the reversible hydrogen electrode (RHE) for COF-366-Co. q-COF-366 exhibited a slightly more positive onset of −0.55 V vs RHE, whereas r- and o-COF-366-Co both have more negative onset potentials of −0.61 V and −0.65 V vs RHE, respectively (Figure 4a, inset). This change indicates modification of the catalytic environment which could be due to structural effects, inductive effects from the introduced functionalizations or modified mass transport.^42^ Overall, the functionalized variants attained higher current densities reaching between −12 mA/cm^2^ and −16 mA/cm^2^ at a potential of −0.98 V vs RHE compared to −11 mA/cm^2^ for COF-366-Co (Figure 4a).

**Figure 4:**
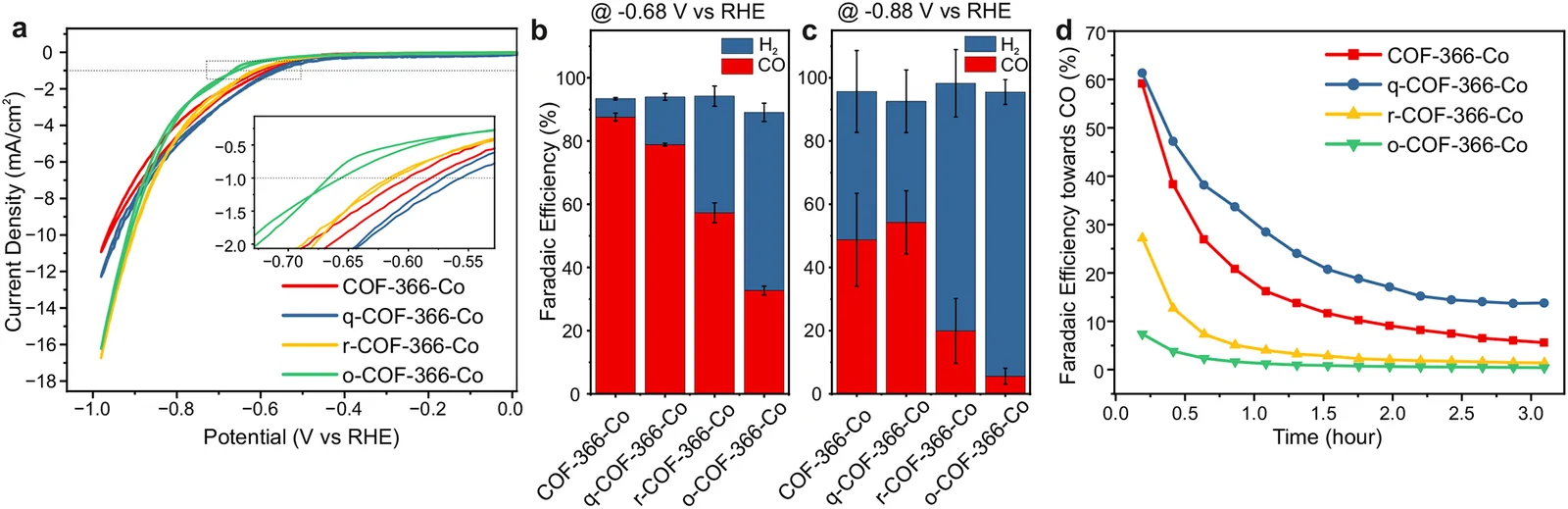
CV (a), initial FE at −0.68 V vs RHE (b), at −0.88 V vs RHE (c), and FE toward CO during a 3 h CA measurement at −0.88 V vs RHE (d) of COF-366-Co, q-COF-366-Co, r-COF-366-Co, and o-COF-366-Co. FE error was calculated from 2 GC injections during a 30 min CA experiment at the indicated potential.

To quantify the products and stability of catalysis, electrolysis was conducted at a fixed potential of −0.68 V and −0.88 V vs RHE, the second being more negative than typically explored potentials toward CO_2_RR for COF-366-Co, but necessary to investigate catalytic stability in more demanding electrocatalytic conditions. The evolution of gaseous products was monitored and quantified by online gas chromatography (GC) (see Experimental Section). COF-366-Co and its postfunctionalized equivalents solely offer CO and H_2_ gas as the products, while the formation of liquid products was not detected (S2.2, Supplementary Figure S2.2.1). As commonly observed in aqueous CO_2_RR, the competing hydrogen evolution reaction (HER) has a large influence on the Faradaic Efficiency (FE) at these negative potentials, and can be facilitated with substrates such as carbon paper. At −0.68 V vs RHE, COF-366-Co offered the highest selectivity toward CO with a FE of 87%, obtained from the average of the first two GC injections; q-, r-, and o-COF-366-Co produced CO with lower FEs of 79%, 57% and 33%, respectively. Interestingly, at the more negative potential of −0.88 V vs RHE, q-COF-366-Co was similar to COF-366-Co in terms of selectivity, producing CO with a FE of 54%, compared to 49% for COF-366-Co. In contrast, r-, and q-COF-366-Co both showed lower selectivity with FEs of 20% and 6%, respectively, demonstrating how PSM can negatively influence selectivity toward CO_2_RR.

In an attempt to understand the observed trends in selectivity toward CO production, water contact angle (WCA) measurements were performed on the electrodes to elucidate hydrophilicity trends. However, no trend was observed, and all electrodes were highly hydrophobic with WCAs ranging from 141.5 ± 1.5° to 147.6 ± 2.5° (S2.6, Supplementary Figures S2.6.1, Supplementary Table S2.6.1). The high electrode hydrophobicity is, however, expected, as the environment which enters in contact with the water is a convolution of catalyst, Nafion as a binder, carbon black, and poly­(1,1,2,2-tetrafluoroethylene) coated (5%) carbon paper. The latter—being largely hydrophobic and present in larger amounts than the catalyst—thus dominates the overall electrode-water interaction, making it difficult to measure the intrinsic hydrophilicity.

COF-366-Co has been reported to offer stable electrocatalysis at mild applied negative potentials.^7^ However, throughout our investigations, we observed the selectivity toward CO to decrease drastically over time at more negative potentials, indicating deactivation. To compare the evolution of stability toward CO production of the pristine and functionalized materials, 3-h CA experiments were performed at −0.88 V vs RHE. With regards to selectivity toward CO over longer electrocatalysis times, q-COF-366-Co performed better than its parent COF, but the FE nevertheless decreased to 14% after 3 h, still remaining over two times higher than that of COF-366-Co which reached 6% after 3 h. The reduced and oxidized versions exhibited an even stronger decrease in selectivity toward CO formation over time, reaching values close to 1%. Recycling experiments across three consecutive chronoamperometric cycles of COF-366-Co interspersed with electrode drying showed irreversible catalyst deactivation without electrode degradation, mirroring the selectivity decline observed in long-term measurements (S2.9, Supplementary Figure S2.9.6). This seemingly strong deactivation can have various causes. Reported deactivation pathways for molecular Co porphyrin catalysts consist of demetalation, reductive carboxylation and oxidation.^43^ Demetalation or the leaching of Co can occur at more negative potentials in which the metal is reduced to Co(0), which can react with water to form Co-oxo or hydroxide species.^43^ The metal content in solution was investigated by performing ICP-OES analysis of the electrolyte after the 3-h chronoamperometric (CA) experiment at −0.88 V vs RHE with COF-366-Co and q-COF-366-Co, revealing that leaching did happen, but that only 0.8–2.3% of the Co present on the electrode leached into solution (S2.7, Supplementary Table S2.7.1). A 17-h CA experiment showed cobalt leaching up to ∼ 11%, showing that long-term measurements with Co-porphyrins are prone to leaching and seems constant at this applied potential (S2.7, Supplementary Table S2.7.1). The results confirm that leaching of Co into solution is happening at these more negative potentials, however, the leaching alone would not account for the 75% or higher loss in activity toward CO production over short times and indicates that additional deactivation pathways or activation of HER processes are most-likely responsible for the observed lower selectivity toward CO production. To probe potential formation of cobalt-containing agglomerates or inorganic cobalt, SEM images of the electrodes before and after 3-h CA measurements were investigated and revealed two distinct populations of particles on all electrodes (S2.8 Supplementary Figure S2.8.2–5). Energy-dispersive X-ray (EDX) mapping identified the larger particles as potassium bicarbonate, a salt expected to form from the electrolyte. The smaller particles (10–50 nm) were, however, below the detection limit of SEM-coupled EDX. Nevertheless, these particles were also present on the blank electrode (i.e., the electrode prepared in an identical manner without the catalyst) postcatalysis, which led us to rule out the *in situ* formation of cobalt, cobalt hydroxide or cobalt oxide nanoparticles. To confirm this, the catalyst on the electrode was scratched off and further analyzed via TEM. TEM coupled with EDX of the detached catalyst layer confirmed the presence of KCl particles (S2.8, Supplementary Figure 4.8.22), which most likely originated from the reference electrode that had been immersed in a KCl solution prior to catalysis, and no Co-containing particles were found. The results above confirm that leaching of Co into solution is happening at these more negative potentials, and that no Co-containing particles form on the electrode. Chemical changes of the COF were probed via FTIR but due to high absorptance of the electrode and carbon black, and the small catalyst amount, no COF-related signals could be observed (S2.5, Supplementary Figure S2.5.4). Postcatalysis morphology analysis was further conducted with grazing incidence X-ray diffraction (GIXRD) of the PSM COF electrodes. Precatalysis and postcatalysis electrodes, however, showed no reflections attributable to the COF frameworks (expected at 2θ = 3.5°, 4.1°, 7.0°, and 8.3°, corresponding to the 100, 010, 200, and 020 reflection planes of the COF, as observed in PXRD of the COF powder) which is attributed to the low sample loading on the electrode as well as the local probing nature of GIXRD, making it difficult to draw conclusions. All GIXRD patterns of the modified COFs resembled that of the blank electrode (only consisting of carbon black and Nafion dropcast onto carbon paper) with reflections at 2θ = 18.0° and 26.6° (S2.1, Supplementary Figure S.4.1.9).

A potential reasoning to understand the selectivity and stability of the various catalysts is through the inductive effects introduced by the functionalizations. It is known that inductive effects can have a large influences on the stability of the catalytic site.^44–46^ From this point of view, in q-COF-366-Co, the introduced −CF_3_ groups, through their electron withdrawing nature, seem to lower the onset potential toward CO_2_RR compared to the pristine COF, as observed in CV measurements (Figure 2a, inset). In the case of r-COF-366-Co, the amine functionality is a known electron-donating group which could favor the above-mentioned deactivation pathways and speed up catalyst degradation. o-COF-366-Co, similar to q-COF-366-Co, also contains electron-withdrawing nitrone groups but, as the initial FE_CO_ is already much lower than that of the pristine COF, it remains difficult to draw conclusions on the extent of deactivation or activation toward HER. Additionally, nitrone-functionalized COFs are known as water harvesters which could locally flood the catalytic sites with water and favor HER.^36^

Taken together, these findings underscore that preserving the intrinsic nanostructure of a COF, including its particle size, morphology, and long-range order, is a prerequisite for any meaningful assessment of PSM effects. In the case of COF-366-Co, we have shown that PSM does not automatically translate into superior catalytic performance; rather, the net outcome reflects a delicate balance among multiple, interdependent variables. Because a single modification inevitably perturbs several of these parameters simultaneously, isolating the contribution of any one factor becomes exceedingly difficult. The effect is even more stark when investigating catalytic activity in terms of PSM concentration, which we will elucidate in the following section.

### Functionalization Concentration-Dependent Induced Disorder

From the results above, q-COF-366-Co emerged as the most promising candidate for delivering more robust CO_2_RR activity at more negative potentials compared to the pristine COF-366-Co. Consequently, we investigated whether increasing the concentration of pendant functionalities could further enhance performance. In the original report of a Povarov functionalization on imine COFs, Li et al. quantified the functionalization amount via X-ray photoelectron spectroscopy (XPS) peak area integration. The degrees of functionalization were found to be between 25 and 29%, which indicates there may be room for higher degrees of functionalization.^34^ However, as introduced above, electrocatalytic studies require PSM reaction conditions that preserve the nanostructure of the material to allow fair performance comparisons and to better understand the fundamental parameters affecting catalysis. While PSMs enable precise tuning of the local pore chemistry, they can simultaneously perturb the COF, introducing defects, altering particle size, reducing crystallinity, or limiting pore accessibility. Therefore, striking an optimal balance between functional group incorporation and structural integrity is essential for the rational design of next-generation catalysts. In our investigations, we observed that increasing the PSM functionalization moiety concentrations does offer access to higher functionalization degrees but can also lead to increased disorder within the material, which correlates to reduced material performance toward CO_2_RR. The PSM was conducted at various functionalization concentrations, ranging from 1:0.1 to 1:2.5 (corresponding to the equivalents of 1-ethynyl-4-(trifluoromethyl)-benzene per imine unit, i.e. imine bond:1-Ethynyl-4-(trifluoromethyl)-benzene) ratios), to assess how the extent of functionalization affects catalysis.

The successful gradual functionalization was monitored through FTIR, revealing an increase in band intensities characteristic of the introduced trifluoromethyl groups (νC–F_3_) at 1323 cm^–1^, 1170 cm^–1^, 1134 cm^–1^, 1068 cm^–1^ (Figure 5a).^47^ Complementarily, increasing covalent functionalization was confirmed through Raman spectroscopy, with features associated with the imine linkage (νC=N) at 1635 cm^–1^, 1602 cm^–1^, and a band associated with benzene ring vibration modes at 1178 cm^–1^ gradually disappearing. At the same time, the band at 1571 cm^–1^ gradually shifts down to 1561 cm^–1^ upon more extensive functionalization, which we attribute to enhanced conjugation within the newly formed quinoline rings (Figure 5b). These changes are consistent with a gradual conversion of imine bonds into a more extended, fused aromatic system. The quantification of PSM is complicated by the fact that many analytical methods are either nonspecific or insensitive to the subtle chemical changes in the framework, and while XPS is frequently employed, its inherent surface sensitivity limits interpretation of bulk modifications. In q-COF-366-Co, the introduction of fluorine atoms allows precise quantification through ^19^F ssNMR analysis with CaF_2_ as an internal standard. For 1:0.5, 1:1.5, and 1:2.5 ratios, fluorine was found to be present at 7.0, 8.3, and 14.2 wt %, corresponding to real functionalization amounts of 38%, 43% and 106%, respectively (S2.4.1, Supplementary Figure S2.4.1.1, Supplementary Table S2.4.1.1). For the 1:2.5 ratio, the exceeding 100% functionalization can potentially be explained by the trapping of 1-ethynyl-4-(trifluoromethyl)-benzene) inside the framework due to functionalization-induced distortion. Additionally, ^19^F ssNMR does not allow differentiation between trapped and covalently reacted functionalization moieties in the COF structure. Surface analysis through XPS showed a similar trend of increasing functionalization, however, when compared with ssNMR, the fluorine-to-cobalt atom ratios were smaller, indicating a potential inhomogeneity in functionalization at the surface vs bulk (S2.4.2, Supplementary Tables S2.4.2.1–2, Supplementary Figures S2.4.2.1–3).

**Figure 5:**
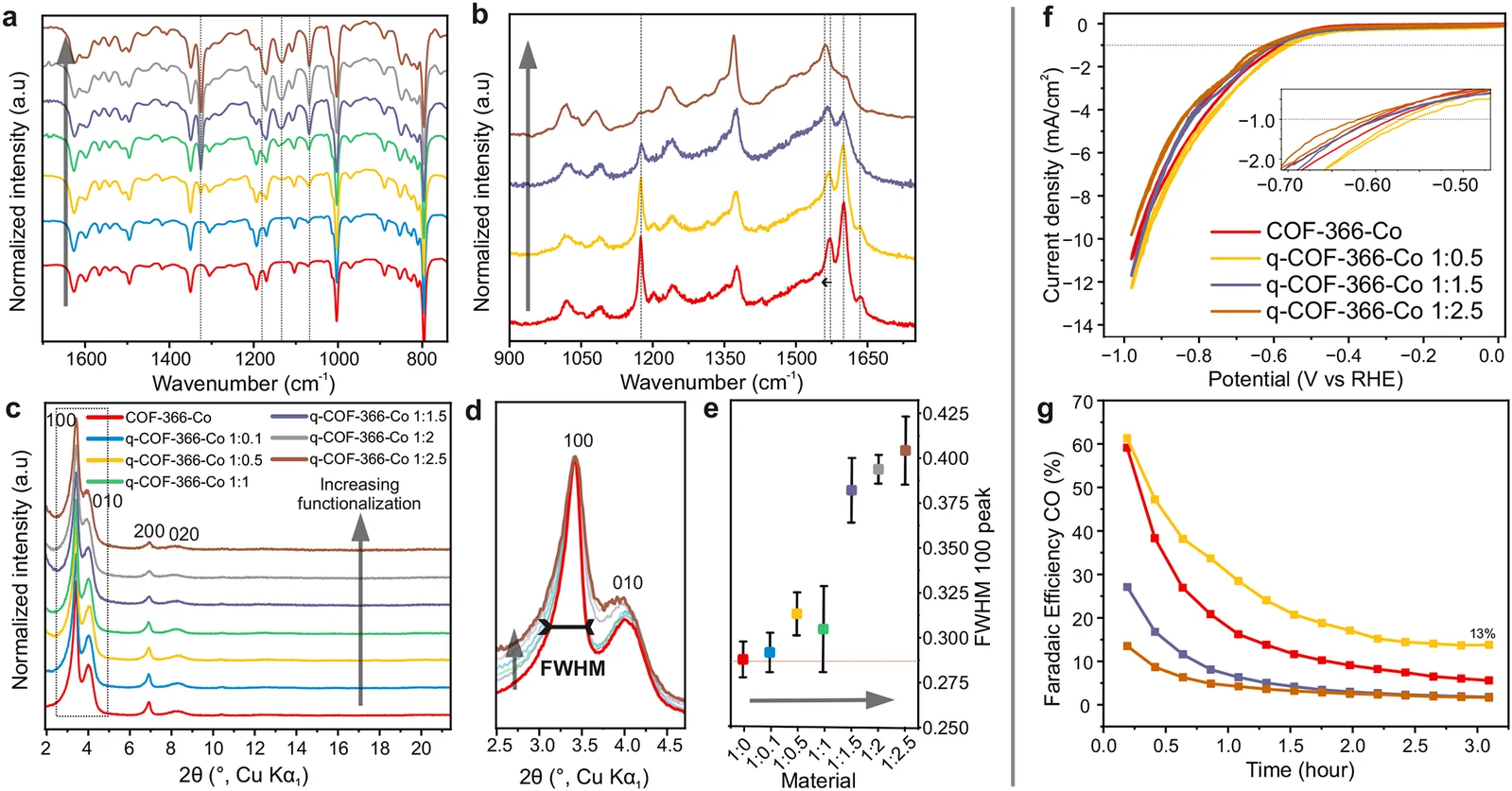
FTIR (a) and Raman spectroscopy (b), normalized PXRD (c), PXRD pattern zoomed into 100 and 010 Bragg peaks (d), fwhm analysis (e), of COF-366-Co and q-COF-366-Co with functionalization concentrations ranging from 1:0.1 to 1:2.5 (imine:ethynyl moiety). Legend for panels a–e is in panel c. CV (f) and FE toward CO of a 3-h CA experiment (g) of COF-366-Co (red) and q-COF-366-Co 1:0.5 (yellow), 1:1.5 (purple), 1:2.5 (brown).

In terms of structure, PXRD of the modified materials demonstrated a progressive decrease in peak intensities along the functionalization series, suggesting reduced crystallinity upon more extensive functionalization (Figure 5c, S2.1, Supplementary Figure S2.1.5). For PXRD, however, various factors such as air scattering and capillary filling can influence peak intensity, so in addition to ensuring all materials be measured on the same device to minimize device correction contributions, an analysis of the 100 peaks was conducted. The results additionally showed an increase in fwhm, correlating to a decrease in average coherent diffraction length within the material. Indeed, the fwhm of the 100 reflection for COF-366-Co was found to be consistent at 0.28 ± 0.01° 2θ over various batches. Functionalization ratios up to 1:1 (imine bond:functionalization moiety) exhibited similar fwhm values up to 0.31 ± 0.01 2θ, but starting at 1:1.5, the fwhm increased to 0.38 ± 0.02° 2θ, reaching 0.40 ± 0.02° 2θ for the 1:2.5 ratio, indicating the introduction of disorder and strain or potential amorphization within the material which effectively reduces the coherent scattering length (Figure 5d,e). To rule out particle size reduction as the cause, TEM images of the samples confirmed the retention of the particle sizes pre- and postfunctionalization for all functionalization ratios, indicating the reduction in coherent scattering likely originates from induced disorder within the framework (S2.8, Supplementary Figure S2.8.1). Nitrogen sorption measurements indicated a gradual decrease in nitrogen gas uptake at higher functionalization ratios, and calculated BET surface areas were reduced from 2194 m^2^/g for the pristine COF-366-Co to 814 m^2^/g for q-COF-366-Co 1:2.5, additionally hinting at induced distortion within the framework, rendering the pores less accessible (S2.3, Supplementary Figures S2.3.1–2, S2.3.5–7). All these results show that even though higher quinoline functionalization can be attained in the framework by tuning PSM concentrations, these come at the cost of increasing disorder throughout the material, potentially then affecting catalysis.

In terms of electrocatalysis, three candidates with low, medium, and high degrees of functionalization (q-COF-366-Co 1:0.5, 1:1.5 and 1:2.5) were subjected toward CO_2_RR at −0.88 V vs RHE. The low functionalized q-COF-366-Co 1:0.5 (previously shown in Figure 4) showed slightly enhanced FE toward CO as well as enhanced catalytic stability over time compared to pristine COF-366-Co. CV showed that low functionalization offered a more positive onset, whereas the highly functionalized samples shift to more negative values, further indicating that functionality and structure play a role (Figure 5f). In terms of selectivity, q-COF-366-Co 1:1.5 and 1:2.5 both showed a much lower initial FE_CO_ of 25%, and 19%, respectively (S2.9, Supplementary Figure S2.9.5). When looking at 3-h CA experiments, q-COF-366-Co 1:0.5 FE_CO_ drops from 61% to 13% which even though being large, is less significant than for FE_CO_ of the pristine COF-366-Co, which drops from 59% to 6%. q-COF-366-Co 1:1.5 and 1:2.5 both completely lose almost all FE toward CO after 3 h, reaching an FE_CO_ of 2% each (Figure 5f). This seems consistent with the increasing structural disorder observed by PXRD analysis. These results, however, show that low functionalization amounts suffice or are even advantageous to modify the pore environment for a more efficient and more stable catalysis toward CO_2_ reduction at more negative potentials.

The postfunctionalization-induced disorder can be rationalized by understanding molecular requirements for the Povarov reaction. The reaction is an aza-Diels–Alder which proceeds though the cycloaddition of a diene (conjugated imine bond of the COF) and a dienophile (alkyne moiety). The cyclization likely proceeds through a stepwise process, but will at one point planarize through chloranil-assisted dehydrogenation. This planarization, combined with the fact that the diene must adopt a planar Z configuration, renders the reaction difficult due to the inherent rotational flexibility and pervasive disorder in COFs. This could mean that many imine sites may be sterically inaccessible for functionalization. However, upon increasing functionalization amounts, and through thermal vibrations, dienes could adopt planar orientations within their environment and react. This would likely further cause the self-amplifying distortion within the framework in the form of sheet buckling and linker twisting, which is consistent with the trends observed in PXRD. As the porphyrin molecular catalysts are embedded into the framework, any buckling and sheet strain induced by a PSM could directly affect the activity of the porphyrin moiety, as the literature has shown that the bending effect, saddling and ruffling of porphyrins can directly influence their electronic properties.^48–52^ This structural disordering likely influences catalytic deactivation through both local electronic perturbations at the Co centers as well as through macroscopic changes in bulk framework conductivity. Nevertheless, disentangling these coupled phenomena remains challenging.

Similar effects were observed in r-COF-366-Co and o-COF-366-Co, both showing a functionalization concentration-dependent structural change. r-COF-366-Co was functionalized with increasing concentrations of formic acid, ranging from 1:5 to 1:14 (imine bond:formic acid stoichiometric ratio). FTIR confirmed increasing functionalization with the disappearance of the stretching associated with imine bond vibrations (νN=C) at 1626 cm^–1^ and the increasing intensity of those associated with amine vibrations (νN–H) at 3405 cm^–1^, 1610 cm^–1^, and 1290 cm^–1^ (S2.5, Supplementary Figure S2.5.1–2). With higher concentration, a decrease in diffraction intensity was observed, beginning at a 1:9 ratio with complete loss of crystallinity being apparent at a 1:12 ratio. In addition to this, the gradual increase in the fwhm of the 100 peak and, interestingly, a gradual shift of the 010 and 020 peaks toward higher 2θ values is observed, indicating an increase in the slipping of the layers which can be attributed to the increase in bond length of the amine (single) over the imine (double) bond (S2.1, Supplementary Figure S2.1.3–4). In the case of o-COF-366-Co, the pristine COF was functionalized with mCPBA in various concentrations from 1:1 to 1:4 (imine bond:mCPBA stoichiometric ratio). FTIR showed gradual increase in functionalization with the disappearance of the imine bond stretching (νN=C) at 1626 cm^–1^, as well as the presence of a nitrone (νN–O) stretching at 1070 cm^–1^ (S2.5, Supplementary Figure S2.5.3). From a functionalization ratio of 1:3 onward, PXRD indicated a stark loss of crystallinity which is likely linked to the increased functionalization concentration and the harsh oxidation conditions (S2.1, Supplementary Figure S2.1.5). Due to the intrinsically lower performance toward CO_2_RR of the lightly functionalized materials, the higher concentrations were not investigated toward electrocatalysis, nevertheless, this demonstrates that optimization of functionalization concentrations is crucial to preserving the framework and conclusively comparing materials.

## Conclusion

In this work we have synthesized a family of postsynthetically modified variants of the benchmark COF-366-Co electrocatalyst and demonstrated that the nature and density of the introduced functionality influence both the electronic environment of the cobalt active site and the long-range order of the framework. Three chemically distinct linkages, notably quinoline (q-COF-366-Co), amine (r-COF-366-Co) and nitrone (o-COF-366-Co), were accessed by cyclization, reduction and oxidation of the native imine bonds, respectively. Comprehensive structural investigation confirmed that the crystallinity of the framework survives the PSM at modest functionalization levels, whereas higher loadings progressively attenuate diffraction intensities, broaden the 100 reflection, and diminish nitrogen uptake, signaling disorder and strain within the COF material.

Electrochemical CO_2_ reduction in aqueous media revealed that only the quinoline-derived material had a higher selectivity toward CO at the demanding potential of −0.88 V vs RHE over the pristine material. At a low functionalization ratio (imine:ethynyl = 1:0.5), q-COF-366-Co delivers a FE toward CO of 54% together with a lower decay of selectivity toward CO production over 3 h, outperforming the pristine COF-366-Co. By contrast, the amine- and nitrone-modified analogues exhibit pronounced HER competition (FE_H_2__ > 70%) and rapid loss of CO production, underscoring the potential effect of electron-donating substituents on the cobalt redox. Incremental increases in the quinoline functionalization (ratios 1:1.5 and 1:2.5) lead, however, to a gradual decline in FE_CO_ that mirrors the concomitant rise in lattice strain observed by PXRD and the reduction of BET surface area.

These findings demonstrate that PSM of COFs can fundamentally reshape the framework, rather than merely decorating an inert scaffold, highlighting a critical structure–stability trade-off in COF-based electrocatalysts. Consequently, any structural perturbation introduced by PSM must be rigorously characterized. While PSM remains a powerful lever for chemically fine-tuning COF-based electrocatalysts, the altered intertwined electronic, geometric, structural and mass-transport properties demand a holistic evaluation for conclusive catalyst comparison. Only by integrating these multidimensional insights can we reliably benchmark and advance COF catalysts for efficient CO_2_ electroreduction.

## Supplementary Material



## Data Availability

The data that support the findings of this study are available in the Supporting Information of this article. The primary data before processing are available in the repository DaRUS (10.18419/DARUS-6295).

## Notes

Synthetic conditions for concentration-dependent functionalizations as well as comprehensive analytical data, including, PXRD, liquid NMR spectra, N_2_ sorption analysis, functionalization quantification via ^19^F solid-state NMR and XPS, FTIR, water contact angle measurements, cobalt leaching tests, SEM/TEM analysis, and electrochemical data (PDF)

## Abbreviations

^13^C NMR: Carbon-13 Magic-Angle Spinning NMR (solid-state)
^19^F NMR: Fluorine-19 Solid-State NMR
BET: Brunauer–Emmett–Teller (surface-area analysis)
BSE: Back Scattered Electrons
CA: Chronoamperometry
CO_2_RR: Electrochemical CO_2_ Reduction Reaction
COF: Covalent Organic Framework
CV: Cyclic Voltammetry
EDX: Energy-Dispersive X-ray
FFT: Fast Fourier Transform
FTIR: Fourier-Transform Infrared Spectroscopy
fwhm: Full-Width at Half-Maximum
GIXRD: Grazing Incidence X-ray Diffraction
HER: Hydrogen Evolution Reaction
ICP-OES: Inductively Coupled Plasma – Optical Emission Spectroscopy
PSM: Postsynthetic Modification
PXRD: Powder X-ray diffraction
RHE: Reversible Hydrogen Electrode
SE: Secondary Electrons
SEM: Scanning Electron Microscopy
ssNMR: Solid-State Nuclear Magnetic Resonance
TEM: Transmission Electron Microscopy
WCA: Water Contact Angle
XPS: X-ray Photoelectron Spectroscopy
