# Supplementary material for: Deconvoluting the Convoluted: Structural Ramifications of Postsynthetic Linkage Modification in COF-366-Co Catalysts
Source: Chem Mater. 2026 Oct 7;38(20):10465–76. doi: 10.1021/acs.chemmater.6c01374 (PMC13718983; doi:10.1021/acs.chemmater.6c01374)
Supplement: Supplementary file 1 [file cm6c01374_si_001.pdf]

Supporting Information:

Deconvoluting the Convoluted: Structural Ramifications of Postsynthetic Linkage Modification in COF-366-Co Catalysts

Samuel Van Gele[a,b,c] , Amelie Heilmaier[a,c], Fabian Heck[a,b,c], Kathrin Küster[a], Armin Schulz[a], Artem Shevchenko[a], Liang Yao[d], Bettina V. Lotsch[a,b,c]*

[a] Max Planck Institute for Solid State Research, Heisenbergstr. 1, 70569 Stuttgart, Germany

[b] Department of Chemistry, Ludwig-Maximilians-Universität (LMU), Butenandtstr. 5-13, 81377 Munich, Germany

[c] Collaborative Research Center 1333, Universität Stuttgart, Pfaffenwaldring 55, 70569 Stuttgart, Germany

[d] South China University of Technology, State Key Laboratory of Luminescent Materials and Devices, 510640 Guangzhou, China

Contents

S1. Postsynthetic modifications of COF-366-Co 3
S1.1 Quinoline linkage modification 3
S1.2 Amine linkage modification 3
S1.3 Nitrone linkage modification 4
S2. Analytical Data 5
S2.1 PXRD Data 5
S2.2 Liquid NMR Spectra 14
S2.3 N_2 Sorption Analysis 15
S2.4 q-COF-366-Co functionalization quantification 23
S2.4.1 ^{19}F solid state NMR 23
S2.4.2 XPS Analysis 24
S2.5 FTIR 27
S2.6 Water Contact Angle measurements 30
S2.7 Cobalt leaching test 32
S2.8 SEM/TEM Analysis 32
S2.9 Electrochemical data 53
References 59

S1. Postsynthetic modifications of COF-366-Co

S1.1 Quinoline linkage modification

For the series of functionalization concentrations, the amounts of reactants were adjusted as described in **Supplementary Table S1.1.1**

Material	COF-366-Co	Chloranil	Toluene	1-ethynyl-4-(trifluoromethyl)benzene	Boron trifluoride-diethyl ether
q-COF-366-Co 1:0.1	21.4 mg, 0.0231 mmol	1.7 mg, 0.007 mmol	10 mL	1.51 µL, 0.009 mmol	1.17, 0.009 mmol
q-COF-366-Co 1:0.5	30 mg, 0.0323 mmol	11.9 mg, 0.049 mmol	10 mL	10.6 µL, 0.647 mmol	8.19 µL, 0.647 mmol
q-COF-366-Co 1:1	12 mg, 0.013 mmol	9.54 mg, 0.0388 mmol	10 mL	8.46 µL, 0.0517 mmol	6.56 µL, 0.517 mmol
q-COF-366-Co 1:1.5	30 mg, 0.0323 mmol	35.8 mg, 0.145 mmol	10 mL	31.7 µL, 0.194 mmol	24.6 µL, 0.194 mmol
q-COF-366-Co 1:2	12 mg, 0.013 mmol	19.1 mg, 0.078 mmol	10 mL	16.9 µL, 0.10 mmol	13.1 µL, 0.10 mmol
q-COF-366-Co 1:2.5	30 mg, 0.0323 mmol	59.6 mg, 0.242 mmol	10 mL	52.9 µL, 0.323 mmol	41 µL, 0.323 mmol

Supplementary Table S1.1.1: Reactant ratios in synthesis of various q-COF-366-Co (from 1:0.1 to 1:2.5, imine:1-ethynyl-4-(trifluoromethyl)benzene ratio)

S1.2 Amine linkage modification

For the series of functionalization concentrations, the amounts of reactants were adjusted as described in **Supplementary Table S1.1.2**.

Material	COF-366-Co	Formic Acid	PhNO2/DO
r-COF-366-Co 1:5	10 mg, 0.0108 mmol	8.36 µL, 0.216 mmol	1 mL
r-COF-366-Co 1:6	10 mg, 0.0108 mmol	10.1 µL, 0.2592 mmol	1 mL
r-COF-366-Co 1:7	10 mg, 0.0108 mmol	11.7 µL, 0.3024 mmol	1 mL

r-COF-366-Co 1:8	10 mg, 0.0108 mmol	13.4 µL, 0.3456 mmol	1 mL
r-COF-366-Co 1:9	10 mg, 0.0108 mmol	15.1 µL, 0.388 mmol	1 mL
r-COF-366-Co 1:10	10 mg, 0.0108 mmol	10.1 µL, 0.432 mmol	1 mL
r-COF-366-Co 1:12	10 mg, 0.0108 mmol	11.7 µL, 0.5184 mmol	1 mL
r-COF-366-Co 1:14	10 mg, 0.0108 mmol	13.4 µL, 0.605 mmol	1 mL

Supplementary Table S1.1.2: Reactant ratios in synthesis of various r-COF-366-Co (from 1:5 to 1:14, imine:formic acid ratio)

S1.3 Nitrone linkage modification

For the series of functionalization concentrations, the amounts of reactants were adjusted as described in **Supplementary Table S1.1.3**.

Material	COF-366-Co	mCPBA	DCM
o-COF-366-Co 1:1	9.9 mg, 0.010 mmol	7.6 mg, 0.044 mmol	1.35 mL
o-COF-366-Co 1:2	10 mg, 0.0108 mmol	15.2 mg, 0.088 mmol	1.35 mL
o-COF-366-Co 1:3	10 mg, 0.0108 mmol	22.8 mg, 0.132 mmol	1.35 mL
o-COF-366-Co 1:4	10 mg, 0.0108 mmol	30.4 mg, 0.172 mmol	1.35 mL

Supplementary Table S1.1.3: Reactant ratios in synthesis of various o-COF-366-Co (from 1:1 to 1:4, imine mCPBA ratio)

S2. Analytical Data

S2.1 PXRD Data

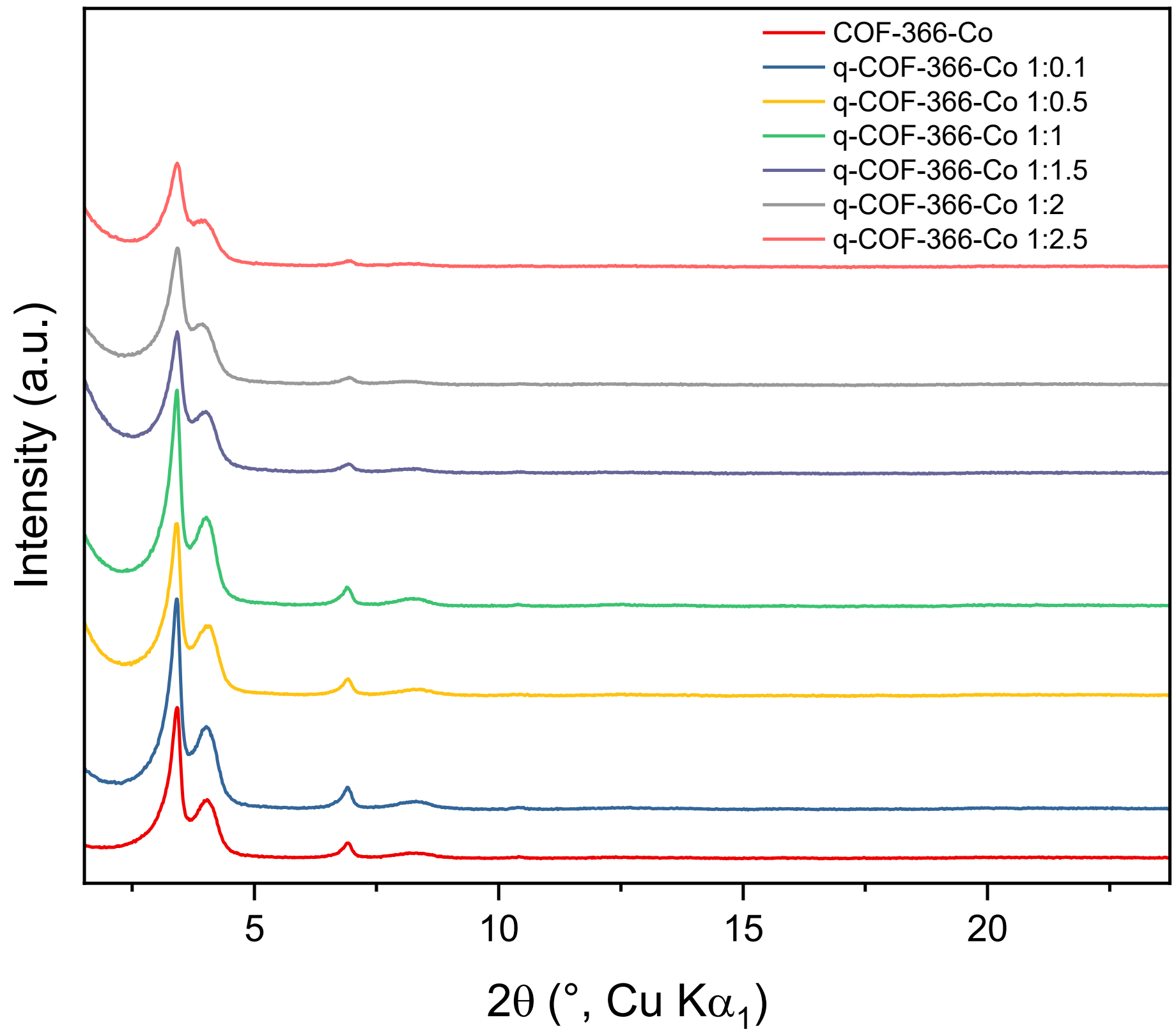

Supplementary Figure S2.1.1: Non-normalized PXRD of q-COF-series

Material	fwhm (100) peak	Error
COF-366-Co	0.28715	0.01
q-COF-366-Co 1:0.1	0.2912	0.011
q-COF-366-Co 1:0.5	0.3129	0.012
q-COF-366-Co 1:1	0.30454	0.024
q-COF-366-Co 1:1.5	0.3822	0.018
q-COF-366-Co 1:2	0.3941	0.008
q-COF-366-Co 1:2.5	0.4047	0.019

Supplementary Table S2.1.1: fwhm of the (100) peak of COF-366-Co and q-COF-366-Co functionalized variants with error. Obtained by fitting two single peak functions with a Chebychev background of order 5 in the range 2.2-10 °

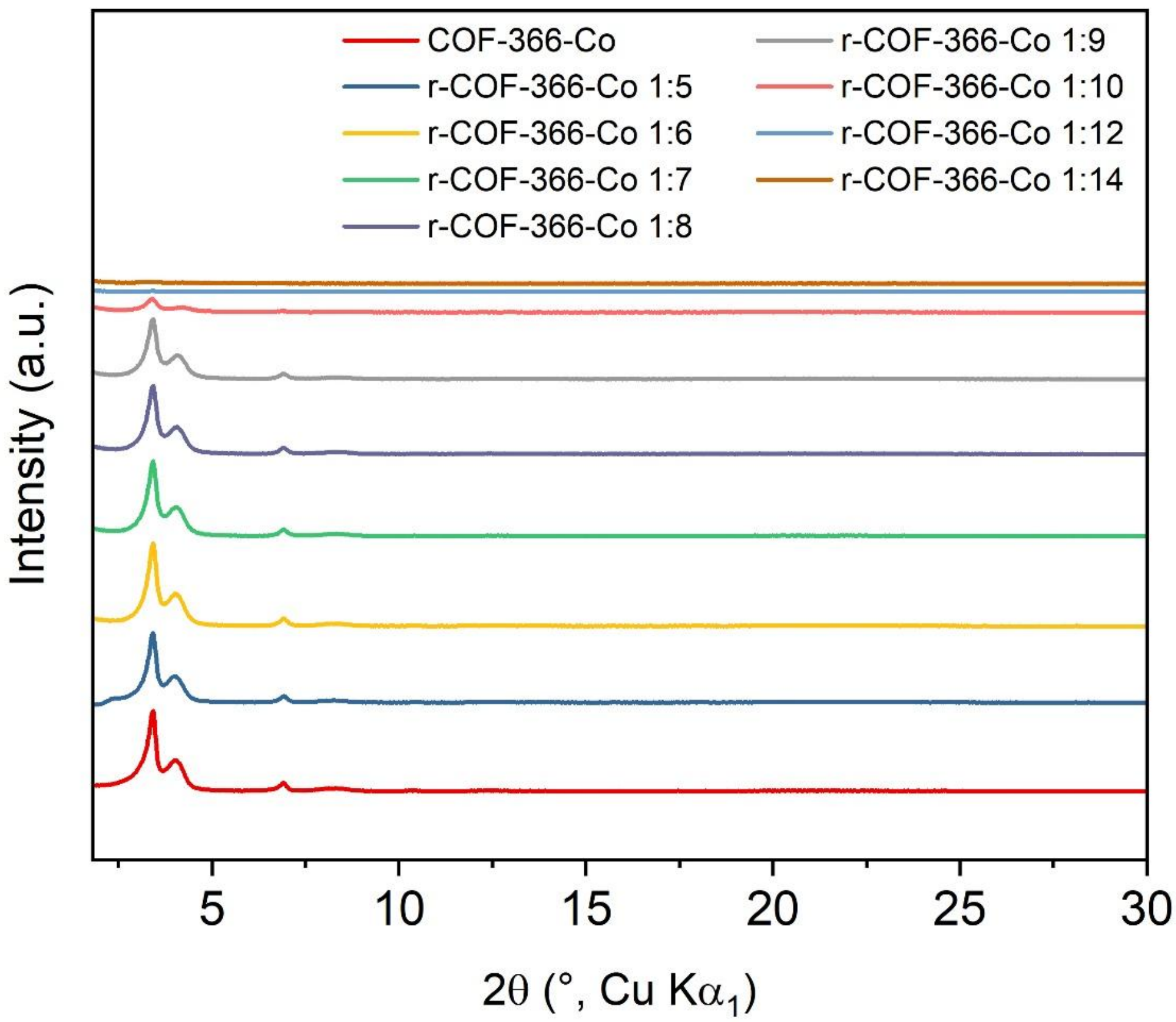

Supplementary Figure S2.1.2: Non-normalized PXRDs of r-COF-series

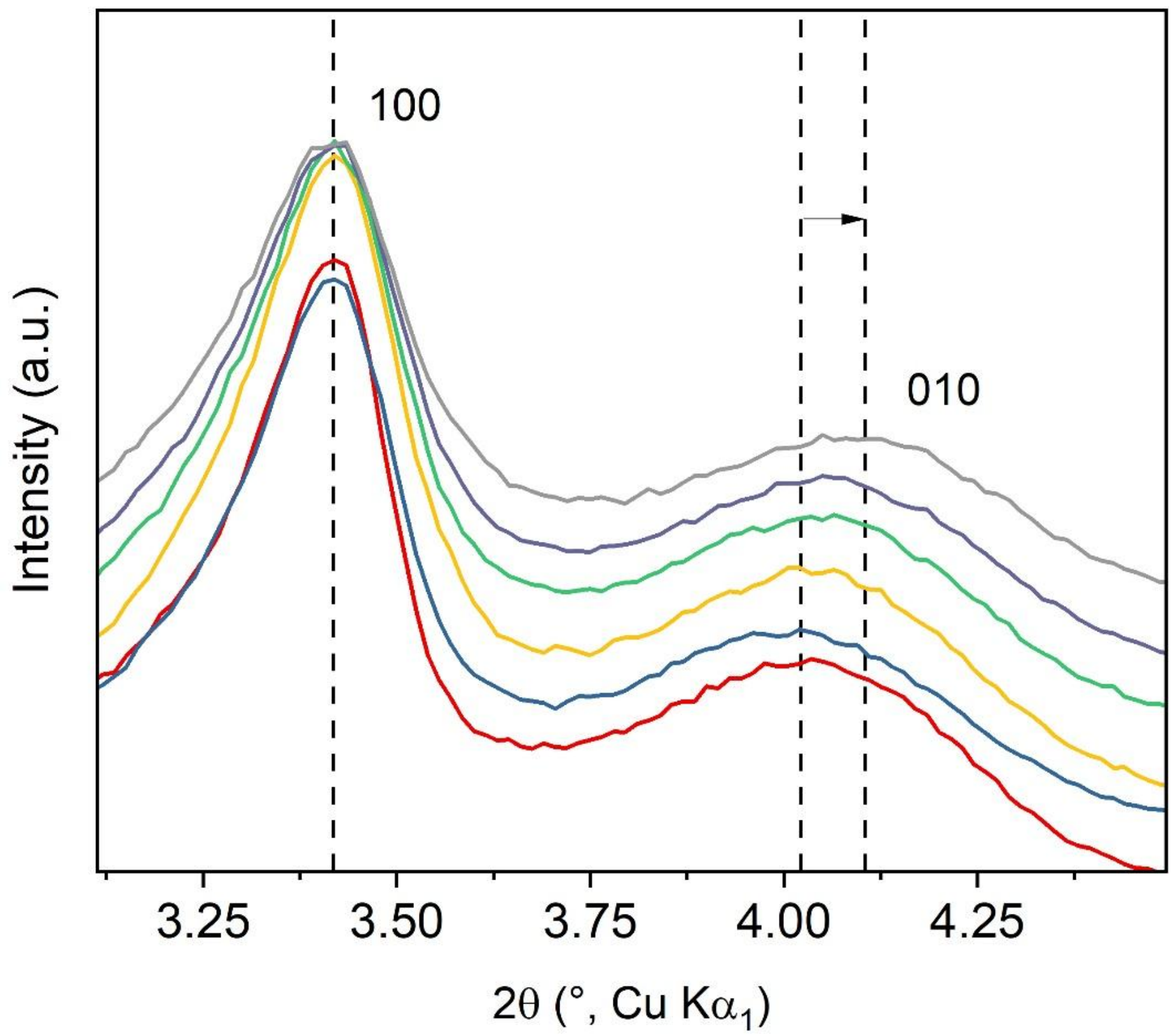

Supplementary Figure S2.1.3: PXRDs of r-COF-series zoomed in to 100 and 010 reflections, with noticeable shift of the 010 peak to higher 2θ values (see Supplementary Figure S2.1.2 for legend, r-COF-366-Co 1:10, 1.12, 1:14 omitted due to low crystallinity)

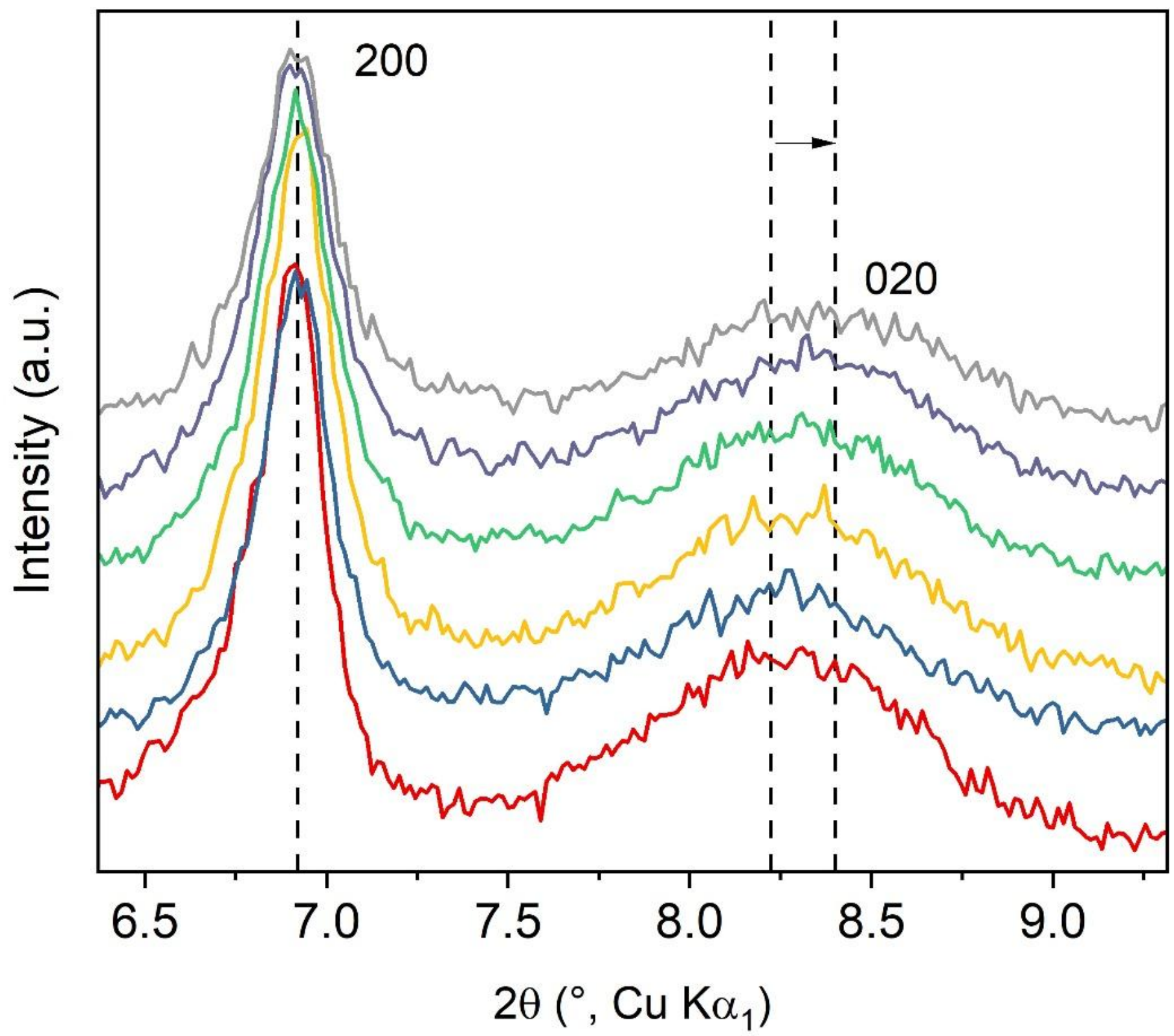

Supplementary Figure S2.1.4: PXRDs of r-COF-series zoomed in to 200 and 020 reflections, with noticeable shift of the 020 peak to higher 2θ values (see Supplementary Figure S2.1.2 for legend, r-COF-366-Co 1:10, 1.12, 1:14 omitted due to low crystallinity)

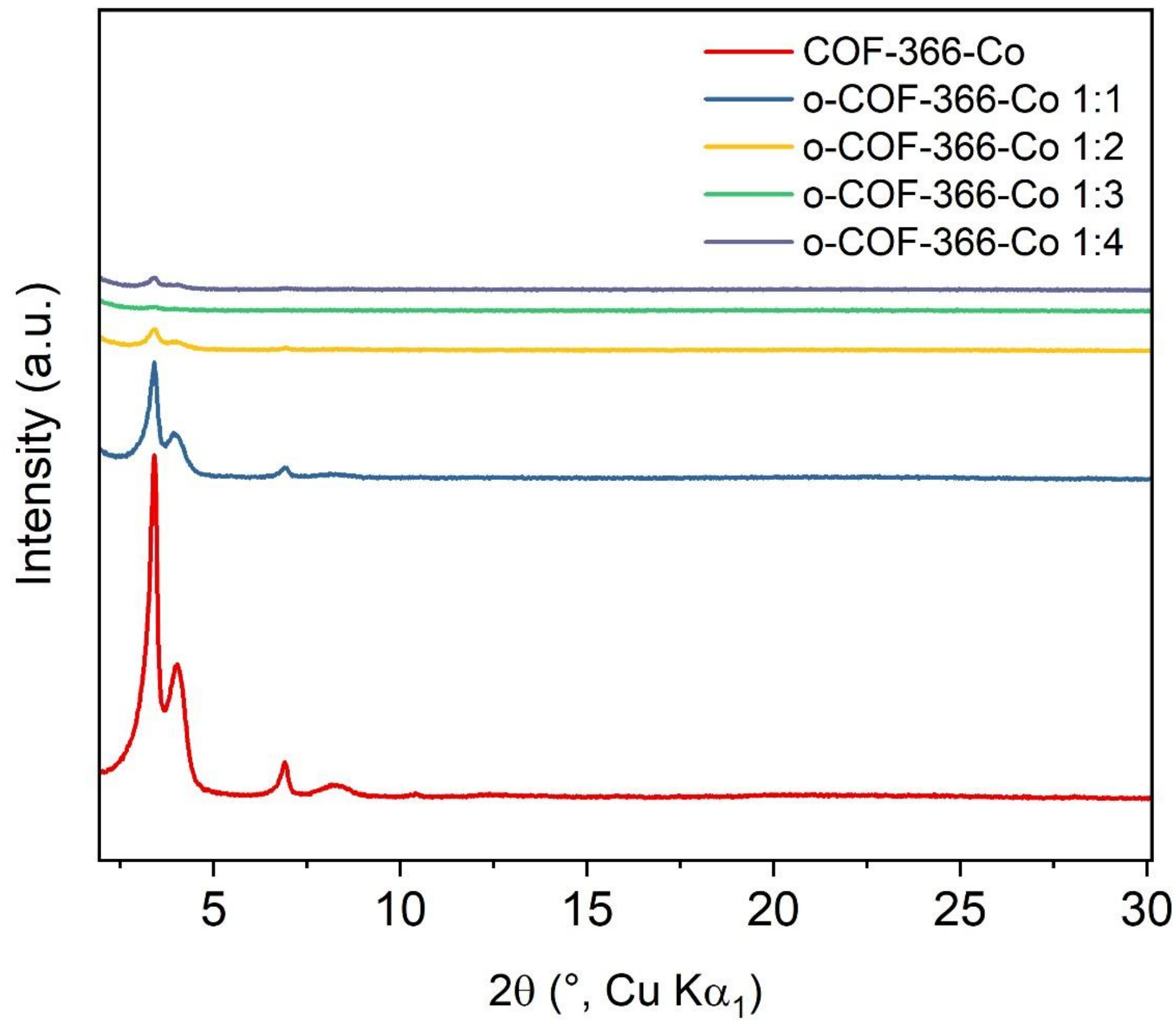

Supplementary Figure S2.1.5: Non-normalized PXRDs of o-COF-series

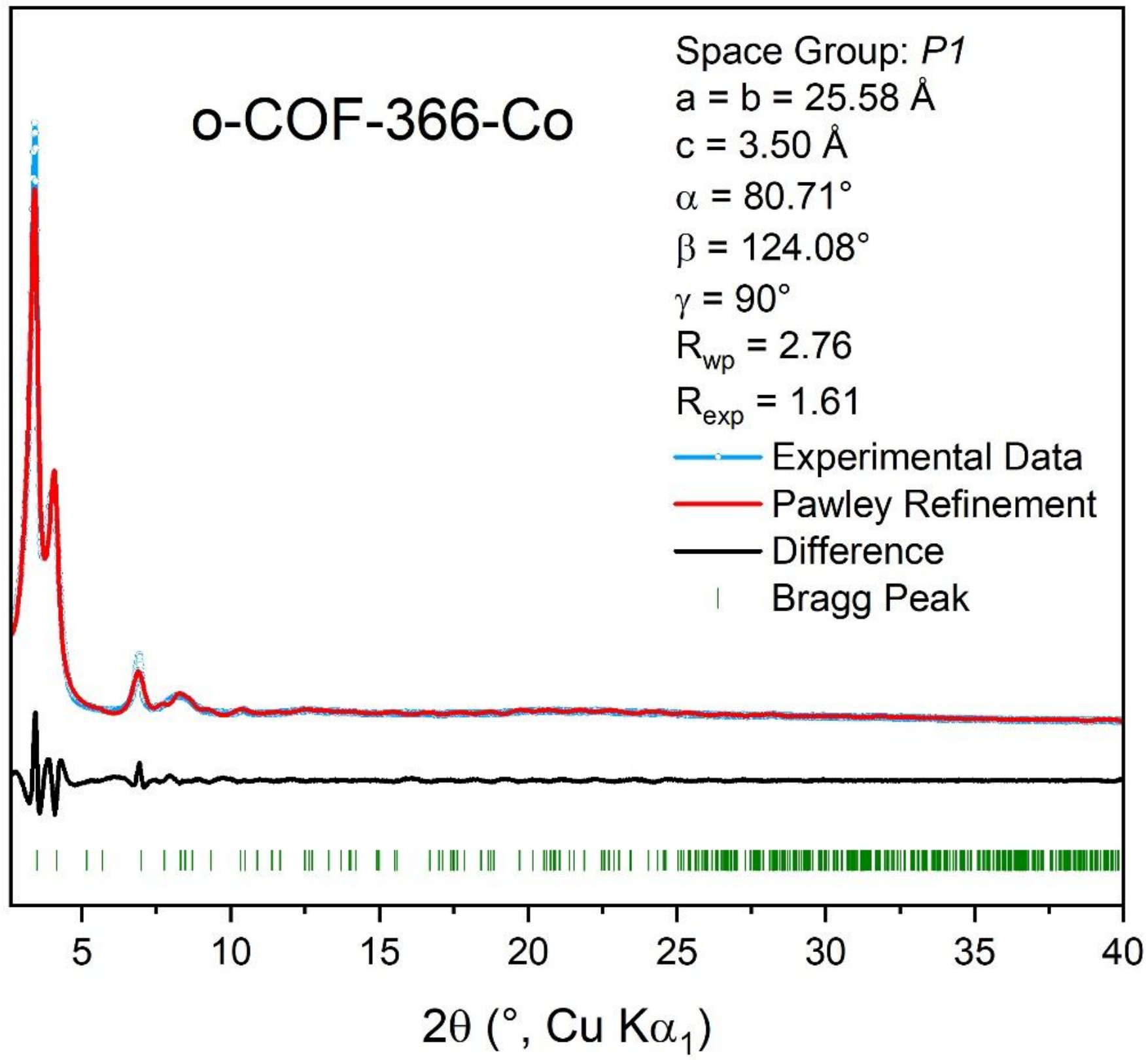

Supplementary Figure S2.1.6: Pawley refinement of o-COF-366-Co

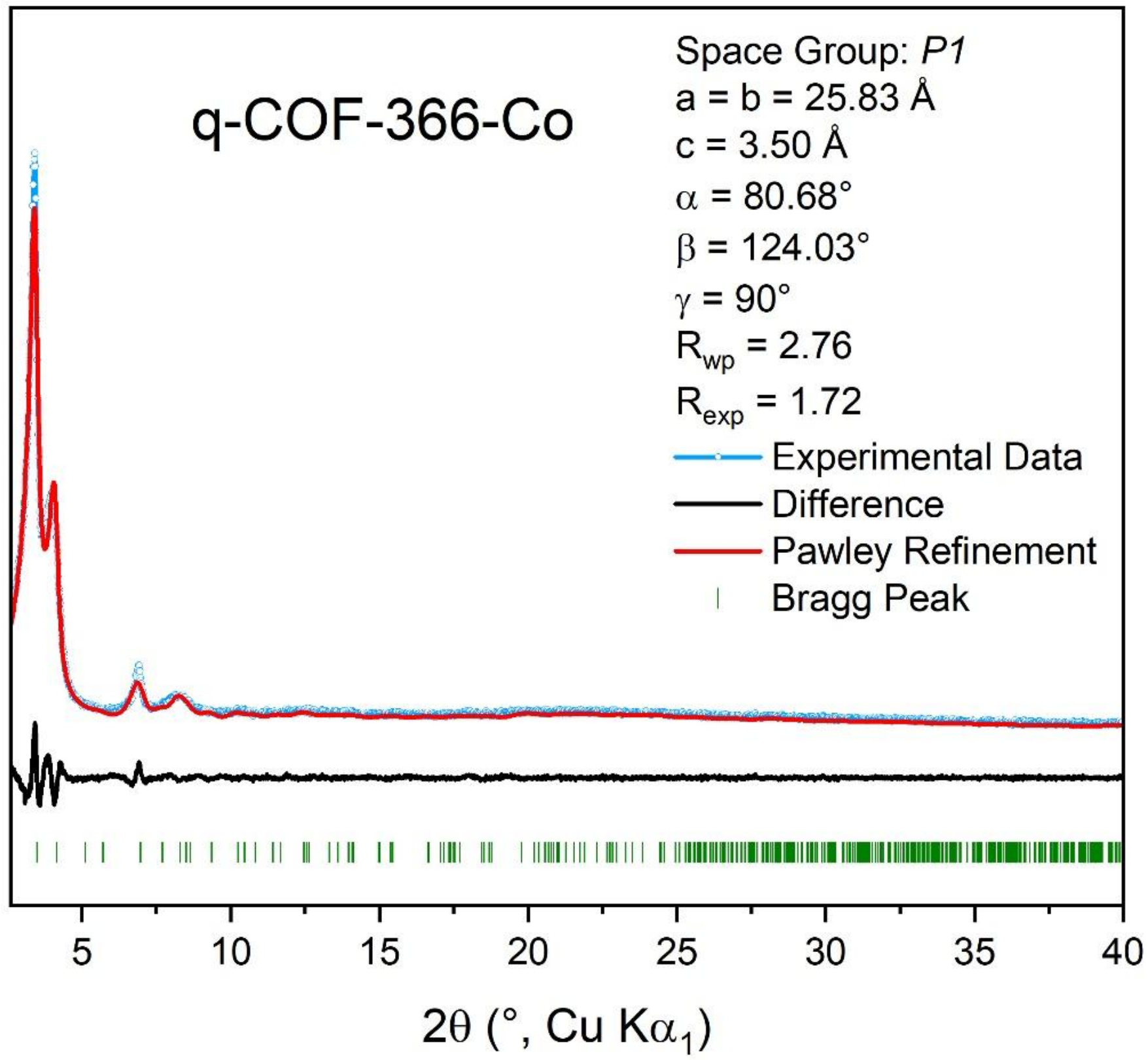

Supplementary Figure S2.1.7: Pawley refinement of q-COF-366-Co

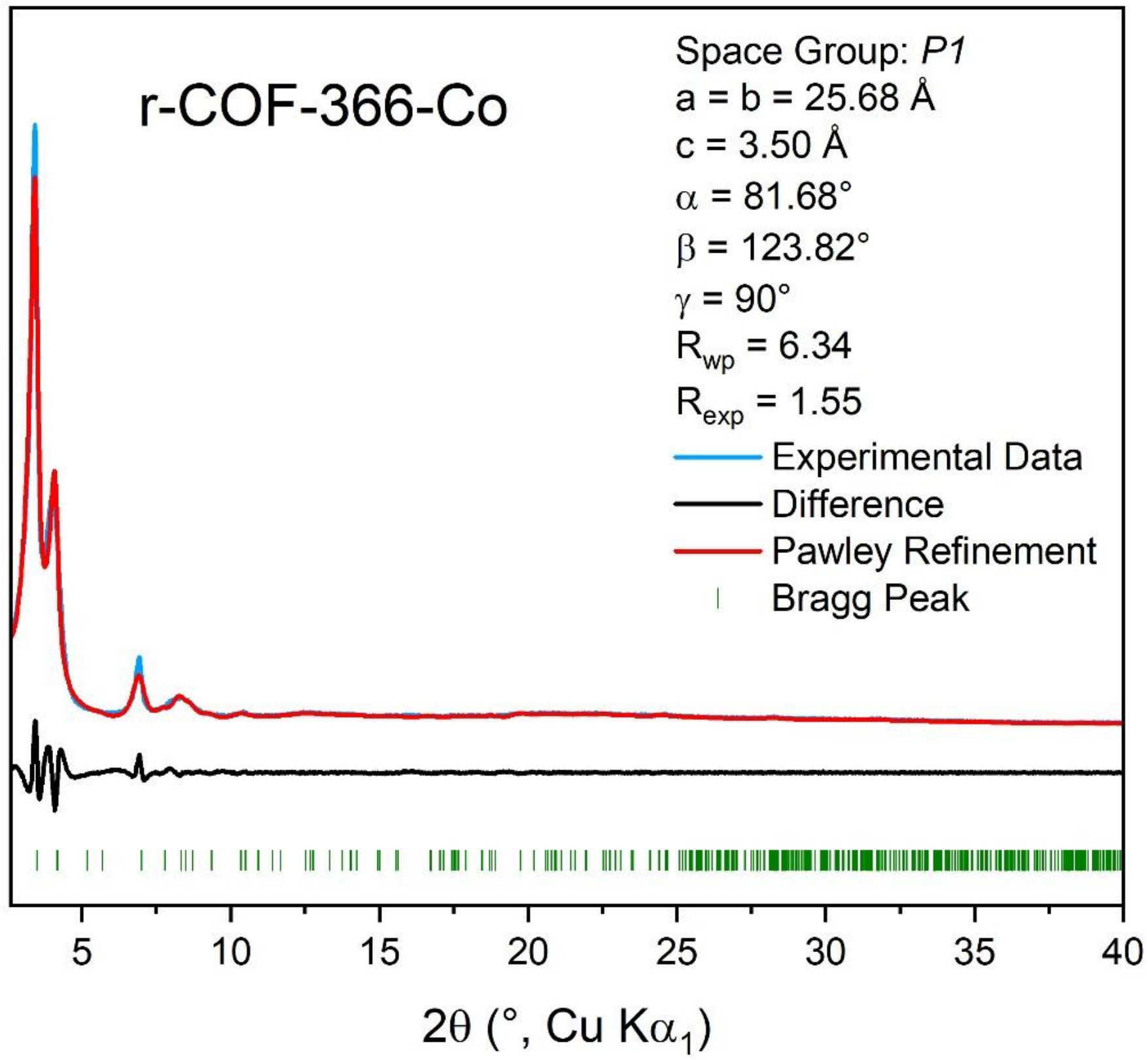

Supplementary Figure S2.1.8: Pawley refinement of r-COF-366-Co

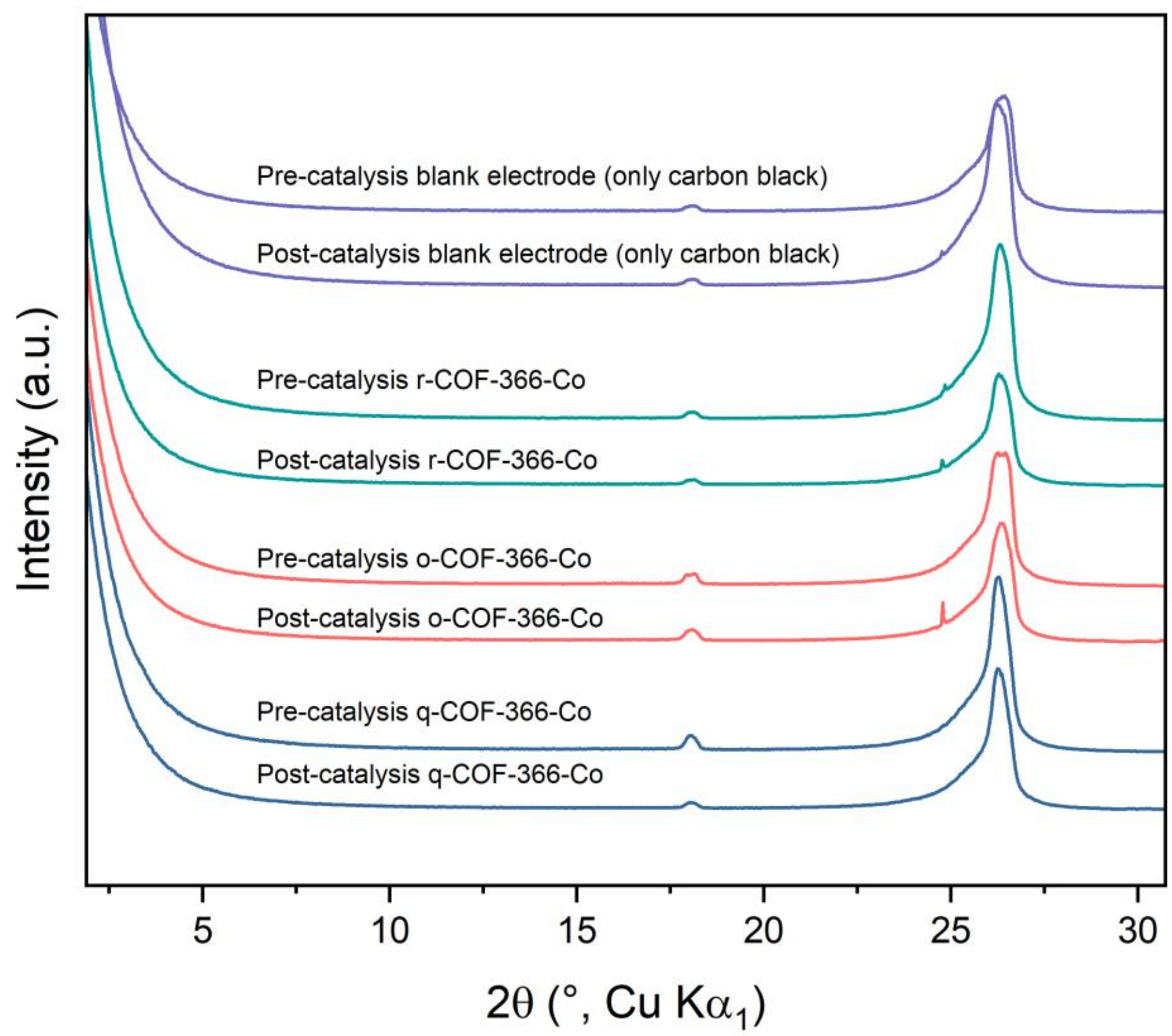

Supplementary Figure S2.1.9: GIXRD of q-, r-, and o-COF-366-Co pre- and post-catalysis.

S2.2 Liquid NMR Spectra

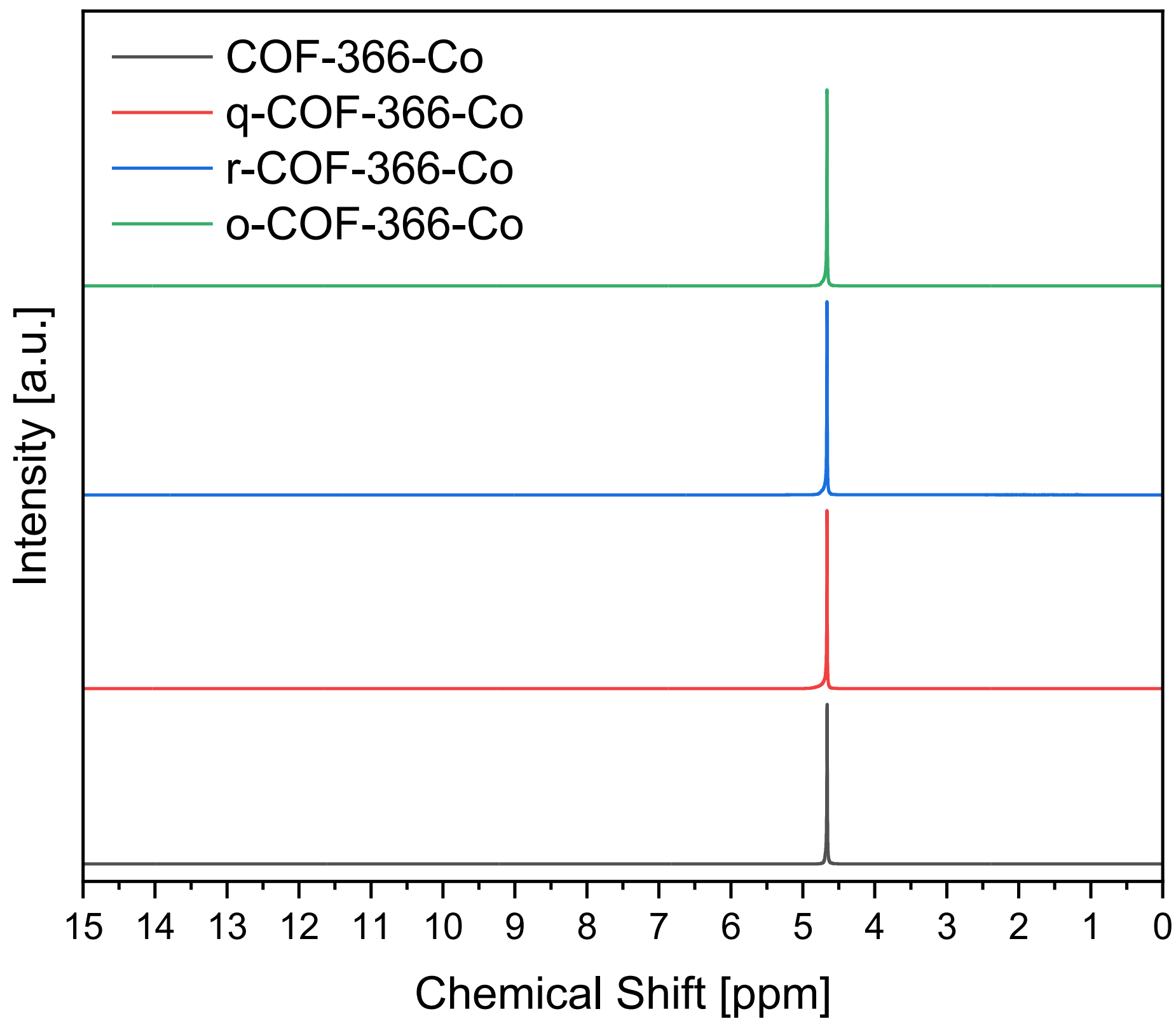

Supplementary Figure S2.2.1: Aqueous electrolyte 1H-NMR analysis. Electrolyte sampled after a 30-minute CA experiment at −0.88 V vs RHE, with DMSO as the internal standard.

S2.3 N_2 Sorption Analysis

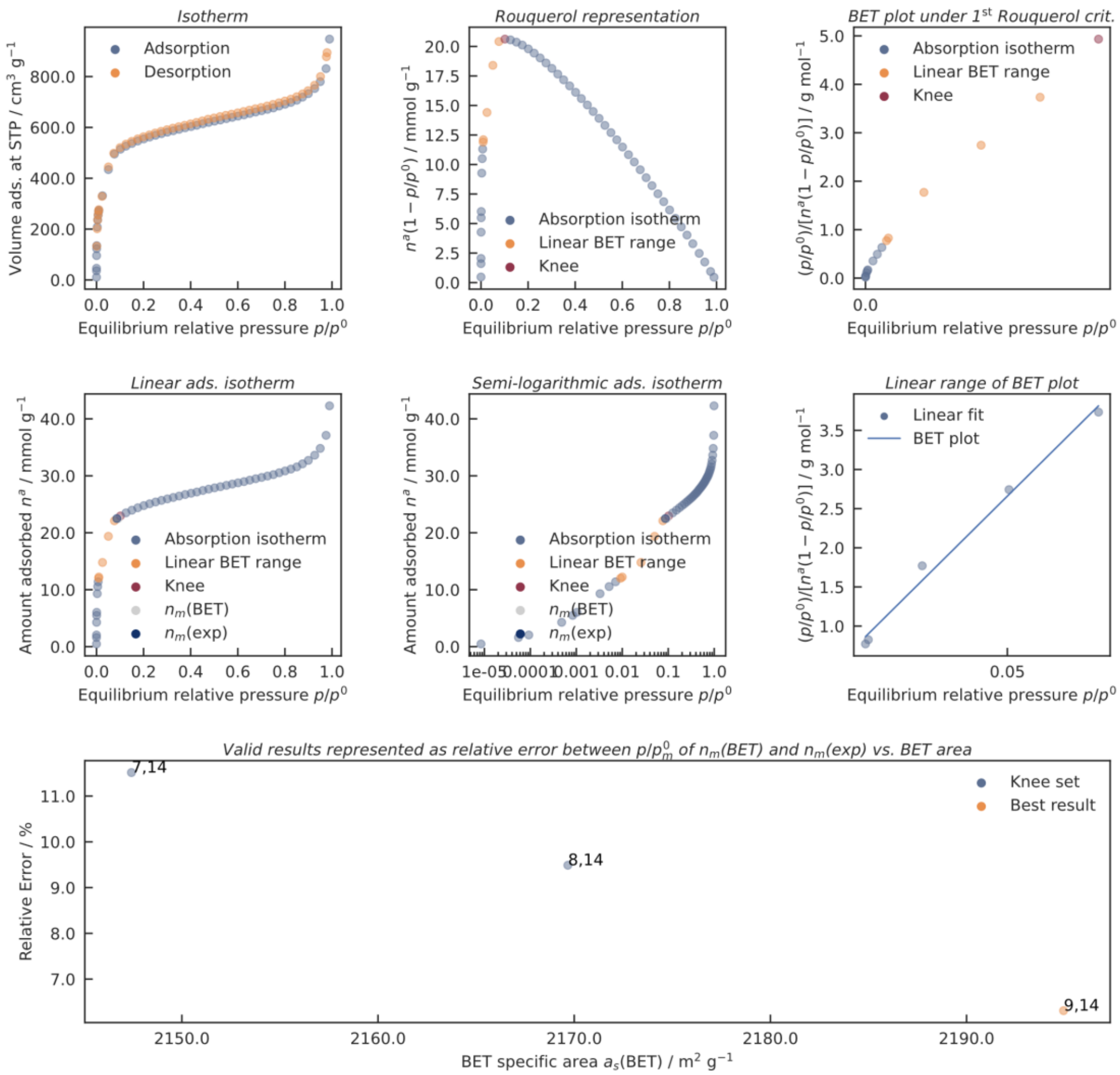

Supplementary Figure S2.3.1: Nitrogen Sorption analysis of COF-366-Co.

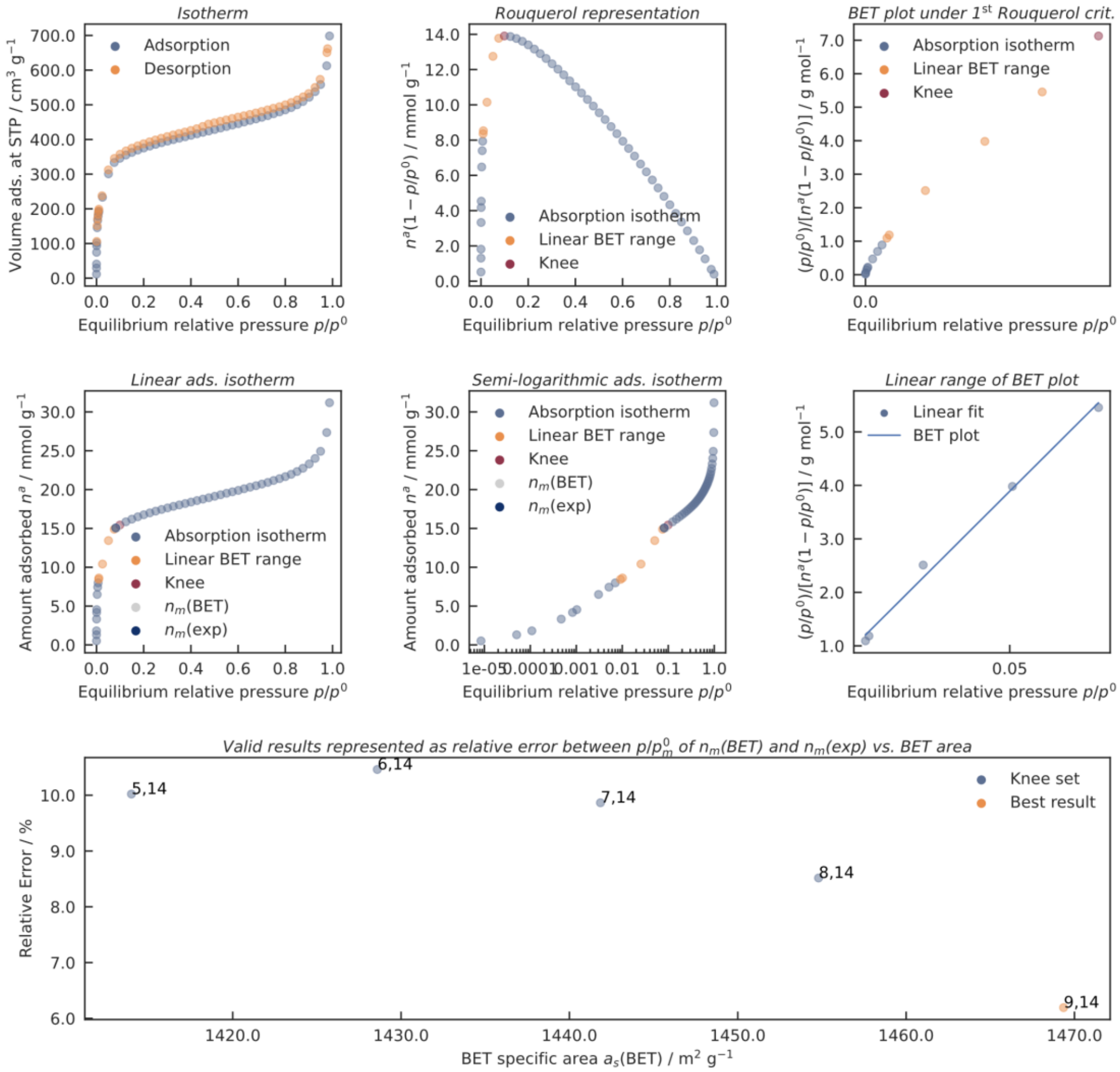

Supplementary Figure S2.3.2: Nitrogen Sorption analysis of q-COF-366-Co (q-COF-366-Co 1:0.5).

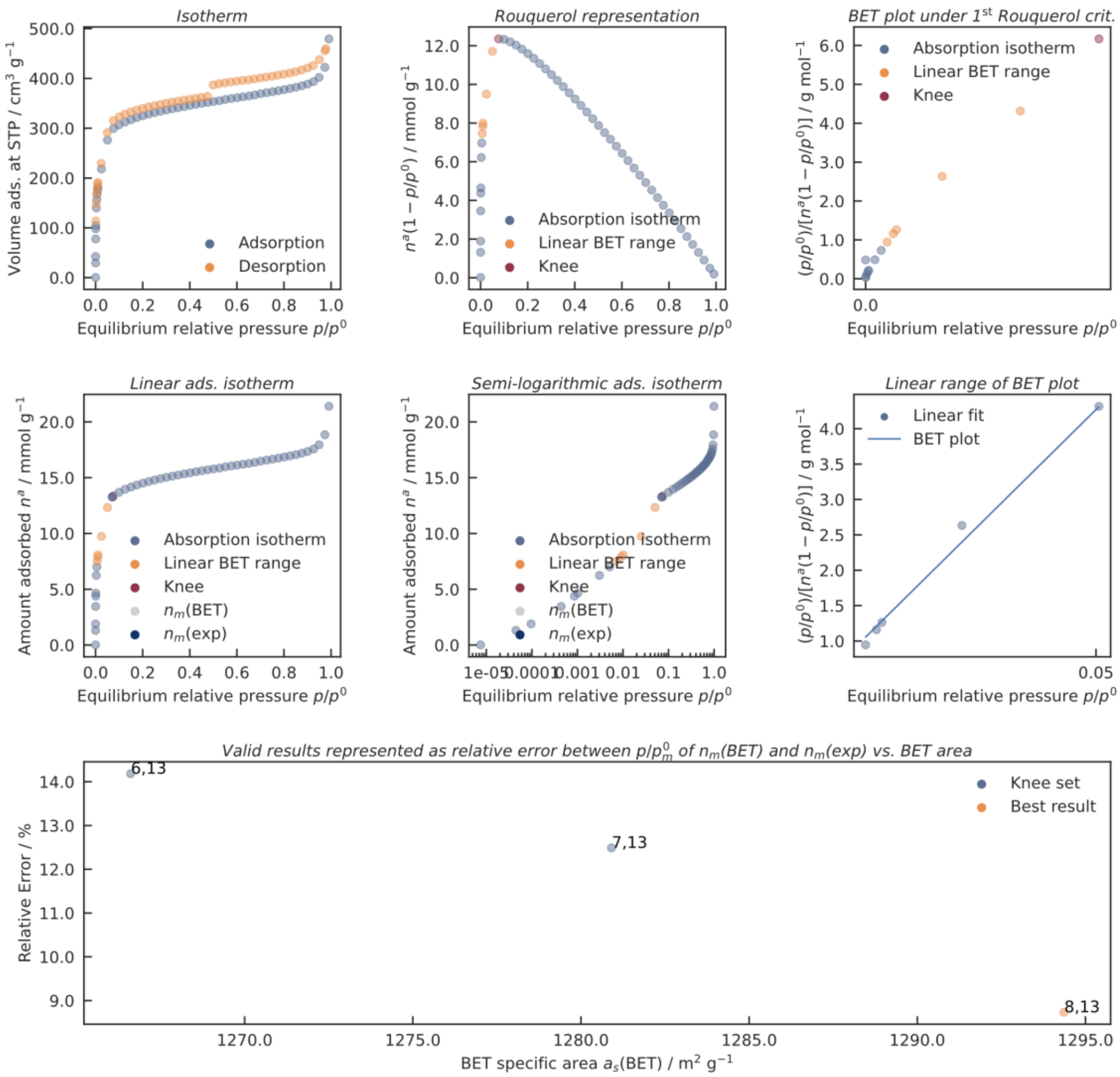

Supplementary Figure S2.3.3: Nitrogen Sorption analysis of r-COF-366-Co.

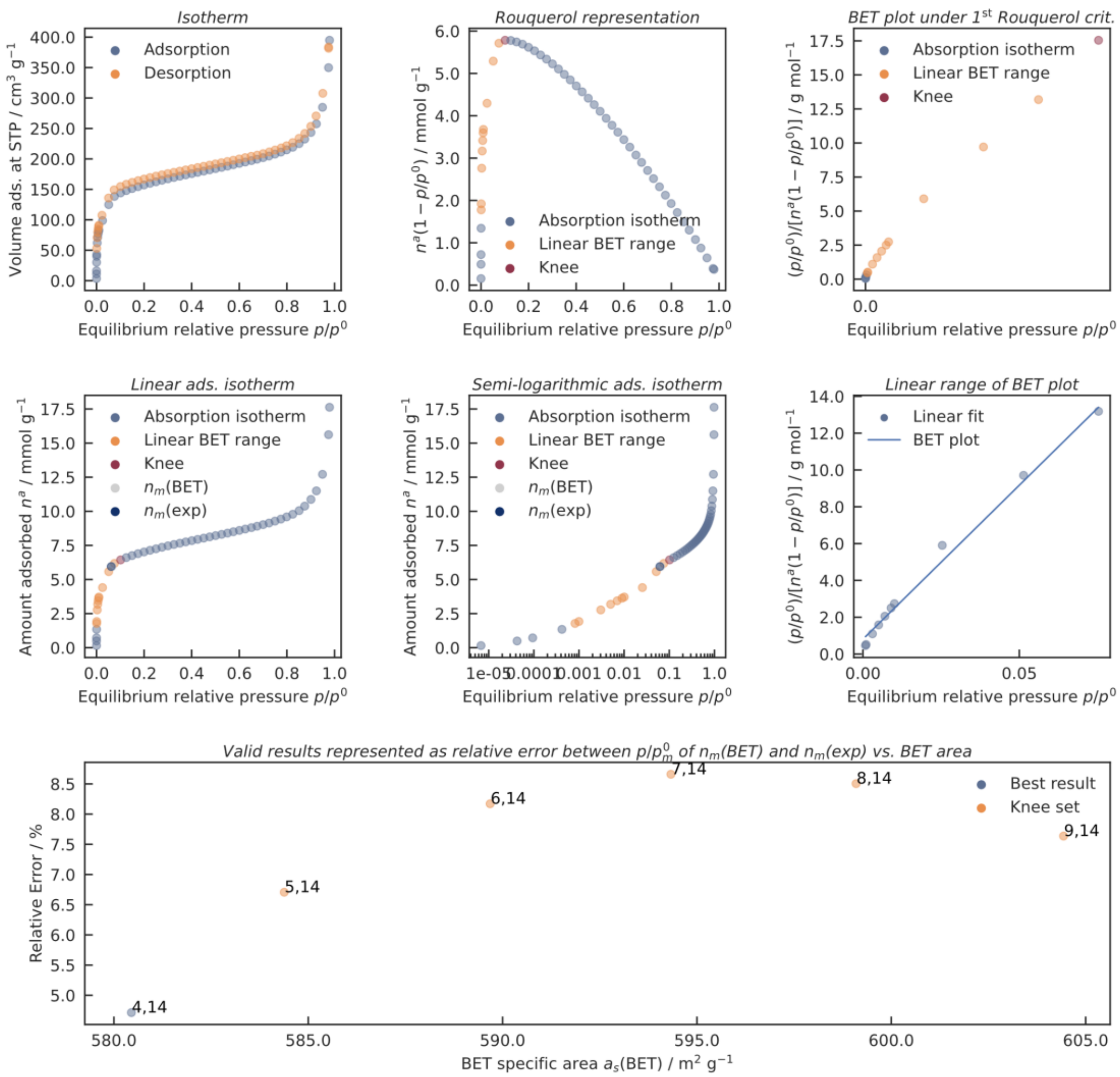

Supplementary Figure S2.3.4: Nitrogen Sorption analysis of o-COF-366-Co.

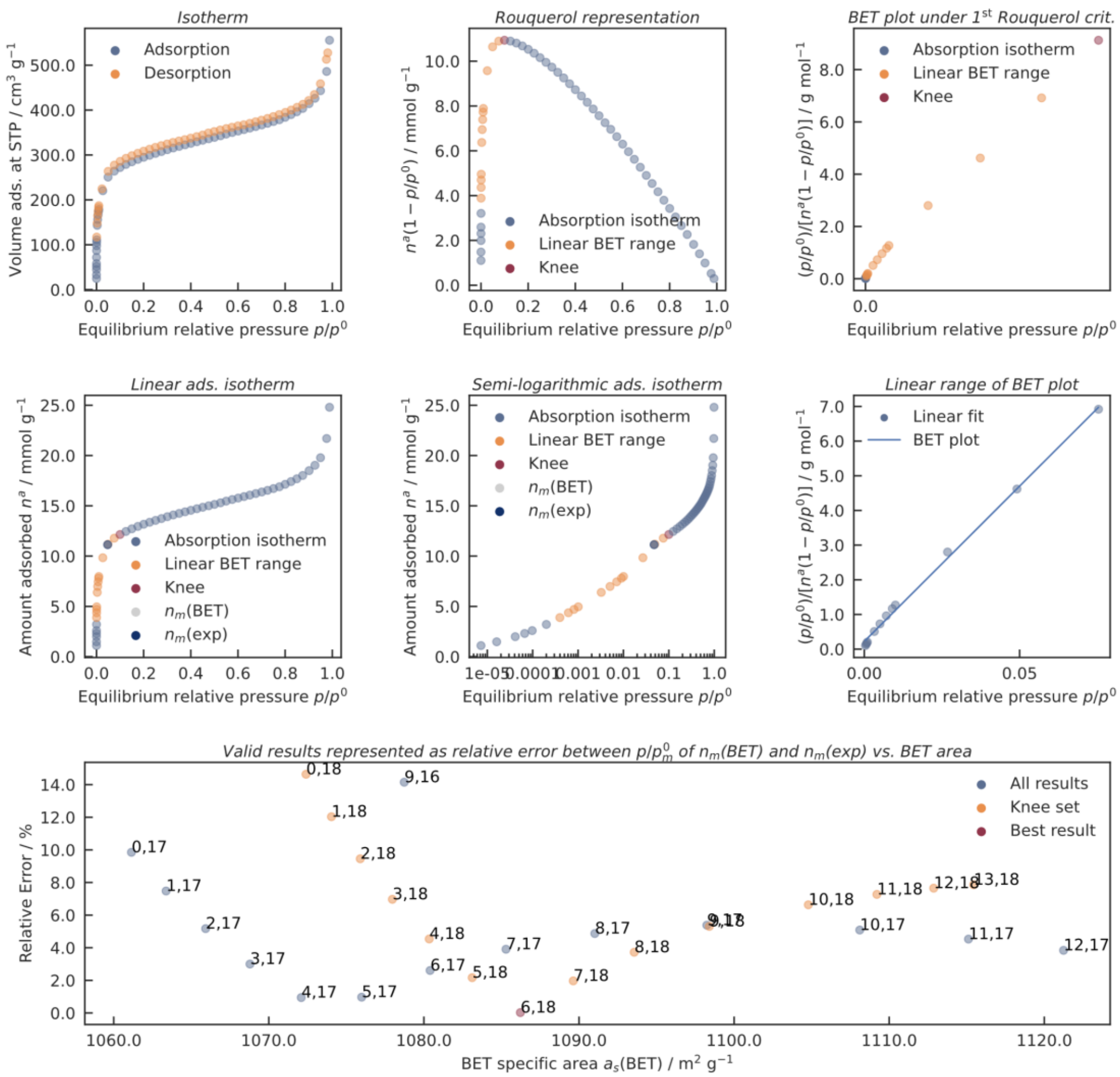

Supplementary Figure S2.3.5: Nitrogen Sorption analysis of q-COF-366-Co 1:1.5.

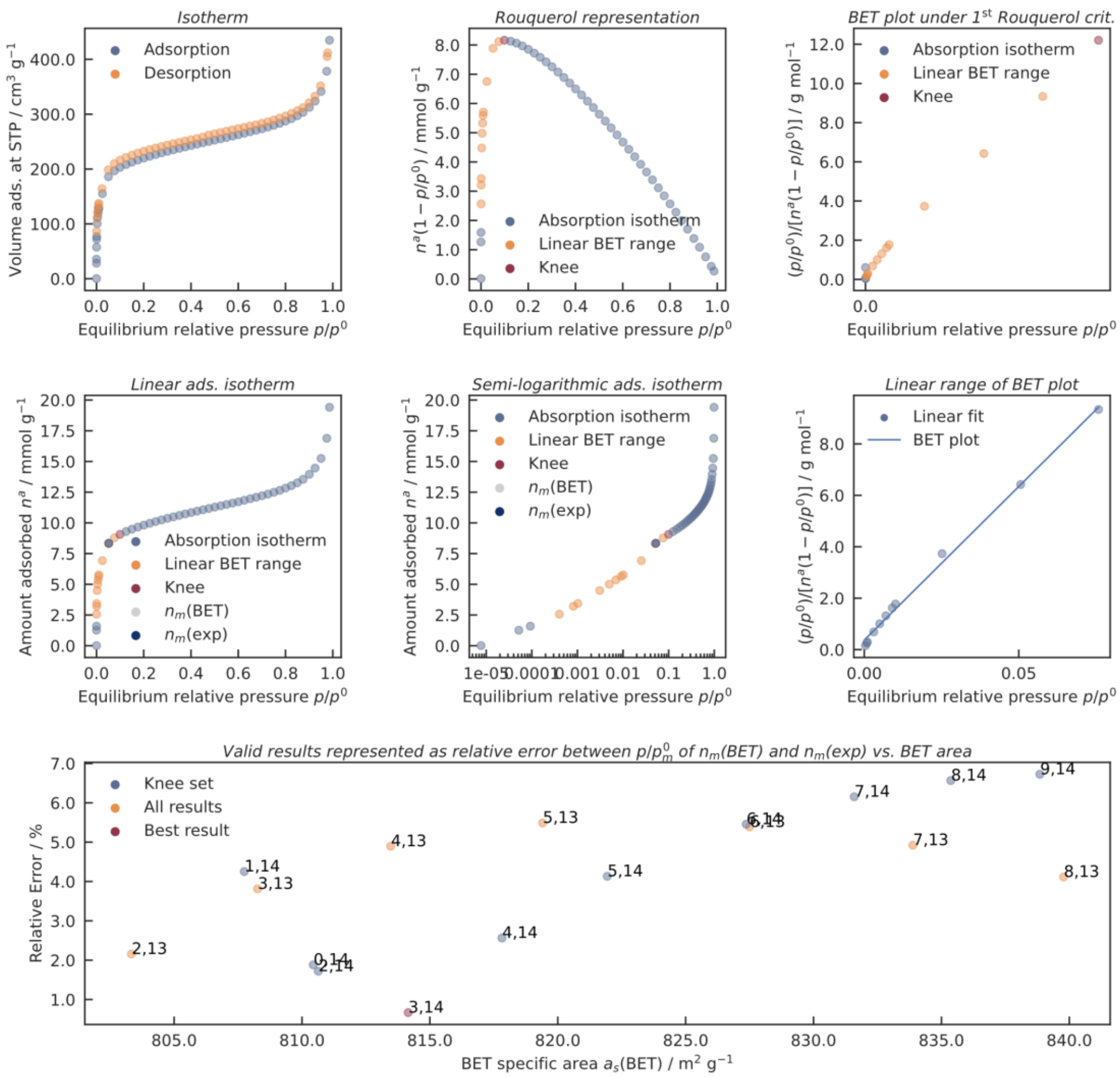

Supplementary Figure S2.3.6: Nitrogen Sorption analysis of q-COF-366-Co 1:2.5.

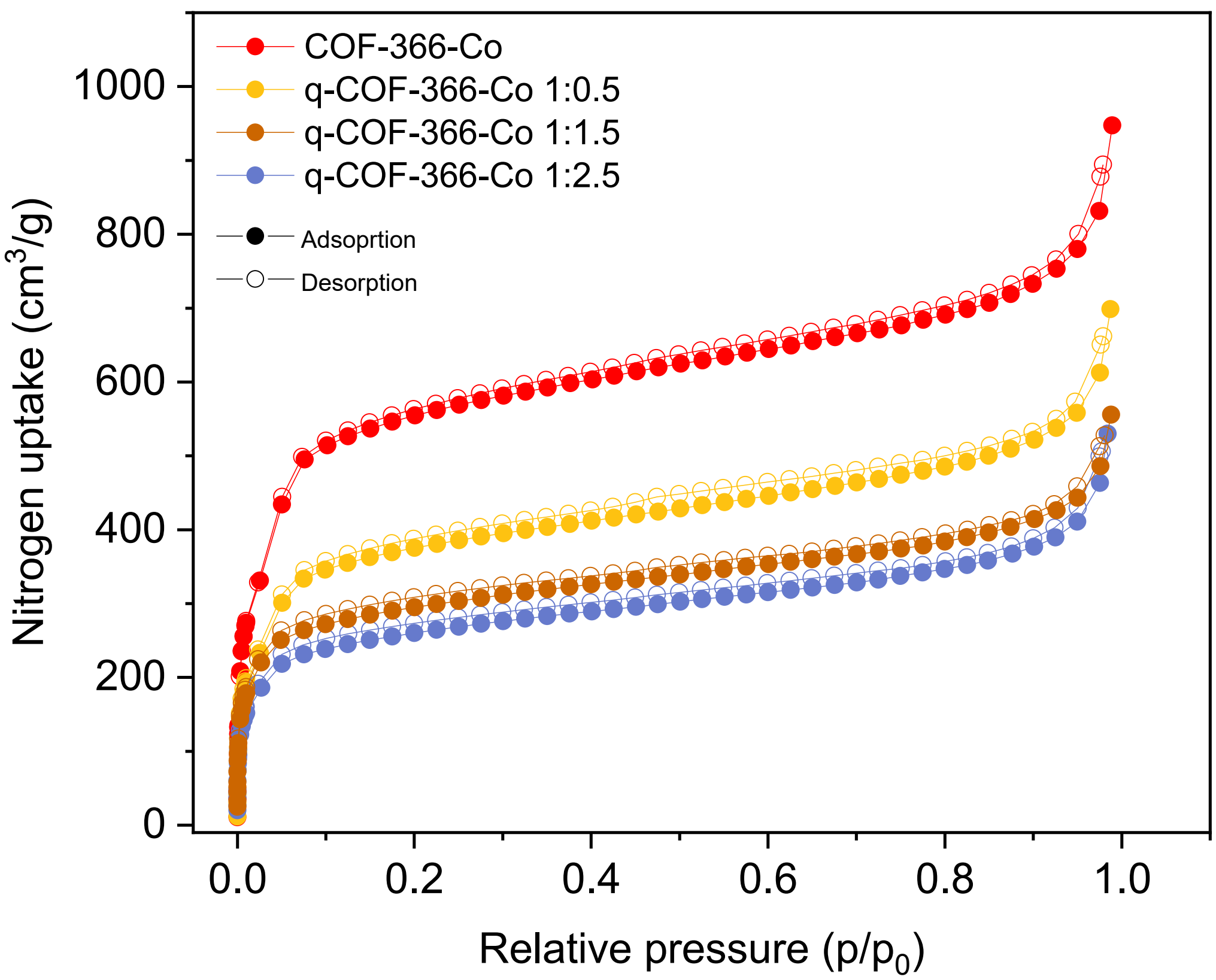

Supplementary Figure S2.3.7: Nitrogen uptake of COF-366-Co and q-COF-366-Co 1:0.5, 1:1.5, and 1:2.5 variants.

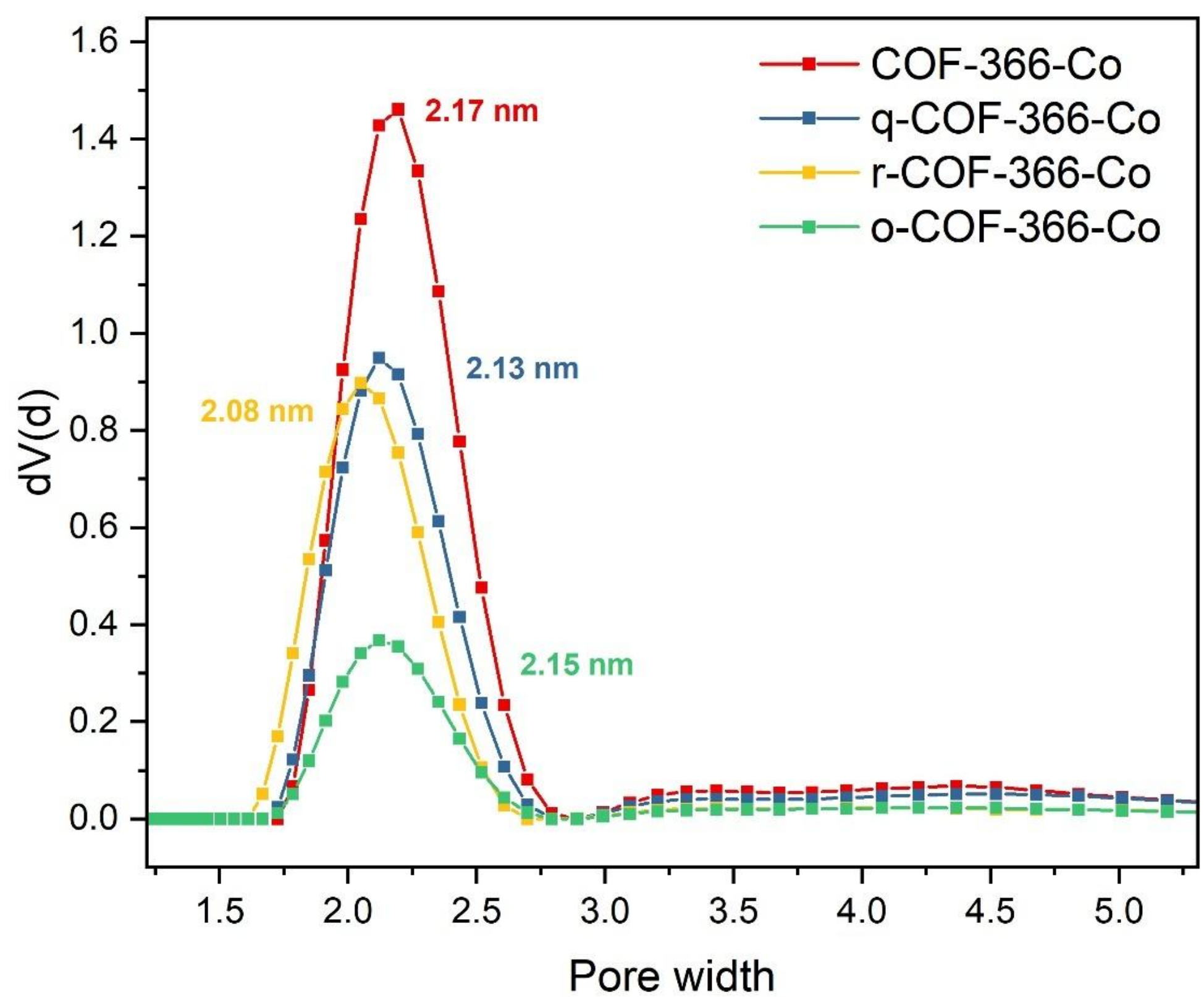

Supplementary Figure S2.3.8: Pore size distribution of COF-366-Co and q-, r-, and o-COF-366- variants. These pore size distributions must be considered lightly as different kernels and pore geometries can offer different results. However, the reducing pore size distribution trend is observed with a model of N_2 at 77 K on carbon using the quenched solid density functional theory (QSDFT) (cylindrical pores, adsorption branch) in AsiQwin software v3.01.

S2.4 q-COF-366-Co functionalization quantification

S2.4.1 ^{19}F solid state NMR

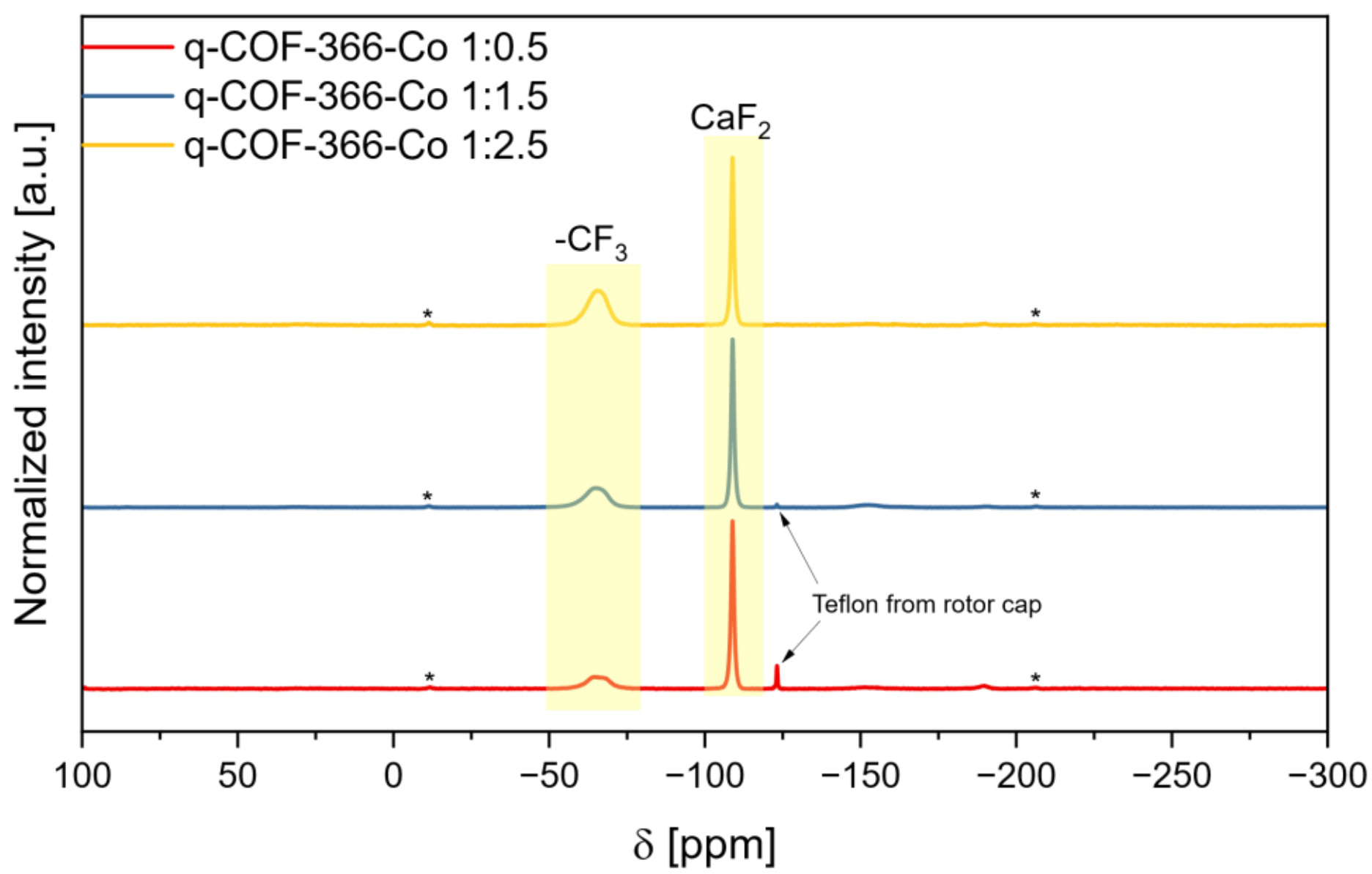

Supplementary Figure S2.4.1.1: ^{19}F ssNMR of q-COF-366-Co series (1:0.5, 1:1.5, 1:25 functionalization ratios) with CaF_2 as internal standard.

Material	Material mass (mg) (a)	CaF_2 mass (mg) (b)	q-COF-366-Co -CF_3 integral (X)	Internal Standard CaF_2 integral (Y)	Mass of F in material (mg) (c)	F wt% in material (Z)
q-COF-366-Co 1:0.5	6.21	1.9	31.37	68.63	0.423	7%
q-COF-366-Co 1:1.5	6.05	1.27	42.75	57.25	0.506	8.3%
q-COF-366-Co 1:2.5	6.00	1.44	54.89	45.11	0.853	14.2%

Supplementary Table S2.4.1.1: ^{19}F ssNMR integrals and derived fluorine wt%

Fluorine percentage in the various materials was calculated as follows:

The integral ratios are proportional to the fluorine content ratios between material and internal standard

$$\frac{X}{Y} = \frac{c}{d}$$

Where X is the integral relating to -CF_3 groups in the functionalized material, Y is the integral relating to fluorine in the CaF_2 internal standard, c is the mass of fluorine in the functionalized material, d is the mass of fluorine in the internal standard.

$$c = d * \frac{X}{Y}$$

From the molar mass of CaF_2, we obtain the weight percentage of fluorine

$$Weight\ Percentage\ Fluorine\ in\ CaF_2 = \frac{2 * M(F)}{M(CaF_2)} = \frac{2 * 19}{78.07} = 0.487 = 48.7\%$$

From this we can determine the mass of fluorine in the internal standard by

$$d = 0.487 * b$$

And thus

$$c = \frac{X}{Y} * 0.487 * b$$

Defining Z as the weight percentage of fluorine inside the material as the ratio between the amount of fluorine in the material and the mass of the material, we obtain:

$$Z = \frac{c}{a} = \frac{X * 0.487 * b}{Y * a}$$

S2.4.2 XPS Analysis

The atom percentages obtained by XPS peak integration are summarized in the table below. The spectra of the relevant components are depicted hereafter.

**Material**	O 1s	C 1s	N 1s	F 1s	Co 2p	Cl 2p
COF-366-Co	2.2%	87.2%	9.7%	N/A	1.0%	N/A
q-COF-366-Co 1:0.5	2.8%	85.5%	9.1%	1.6% total<br>1.07 % -CF_3	0.8%	0.2%
q-COF-366-Co 1:1.5	6.2%	78.6%	8.4%	6.0% total<br>4.46 % -CF_3	0.8%	N/A
q-COF-366-Co 1:2.5	2.6%	85.9%	6.3%	5.4% total<br>5.12 % -CF_3	0.6%	0.4%

Supplementary Table S2.4.2.1: Atom percentages obtained from XPS Analysis of COF-366-Co, q-COF-366-Co 1:0.5, 1:1.5, and 1:2.5.

In the spectra, two fluorine species can be observed due to the presence of boron trifluoride-diethyl ether (binding energy = 685.6 eV) as the reactant material and the incorporated -CF_3

functionality (binding energy = 688.5 eV). For calculations, the integral of the -CF_3 peak was taken. The spectra also showed the presence of chlorine, which is due to the chloranil reagent.

Material	Bulk atom ratio (F at% from ss-NMR/Co at% from ICP-OES)	Surface ratio (XPS F to Co atom percentages ratio)
q-COF-366-Co 1:0.5	4.4	1.3
q-COF-366-Co 1:1.5	5.7	5.6
q-COF-366-Co 1:2.5	10.5	8.5

Supplementary Table S2.4.2.2: Atom ratios of F/Co compared and calculated from XPS, ICP-OES, ssNMR.

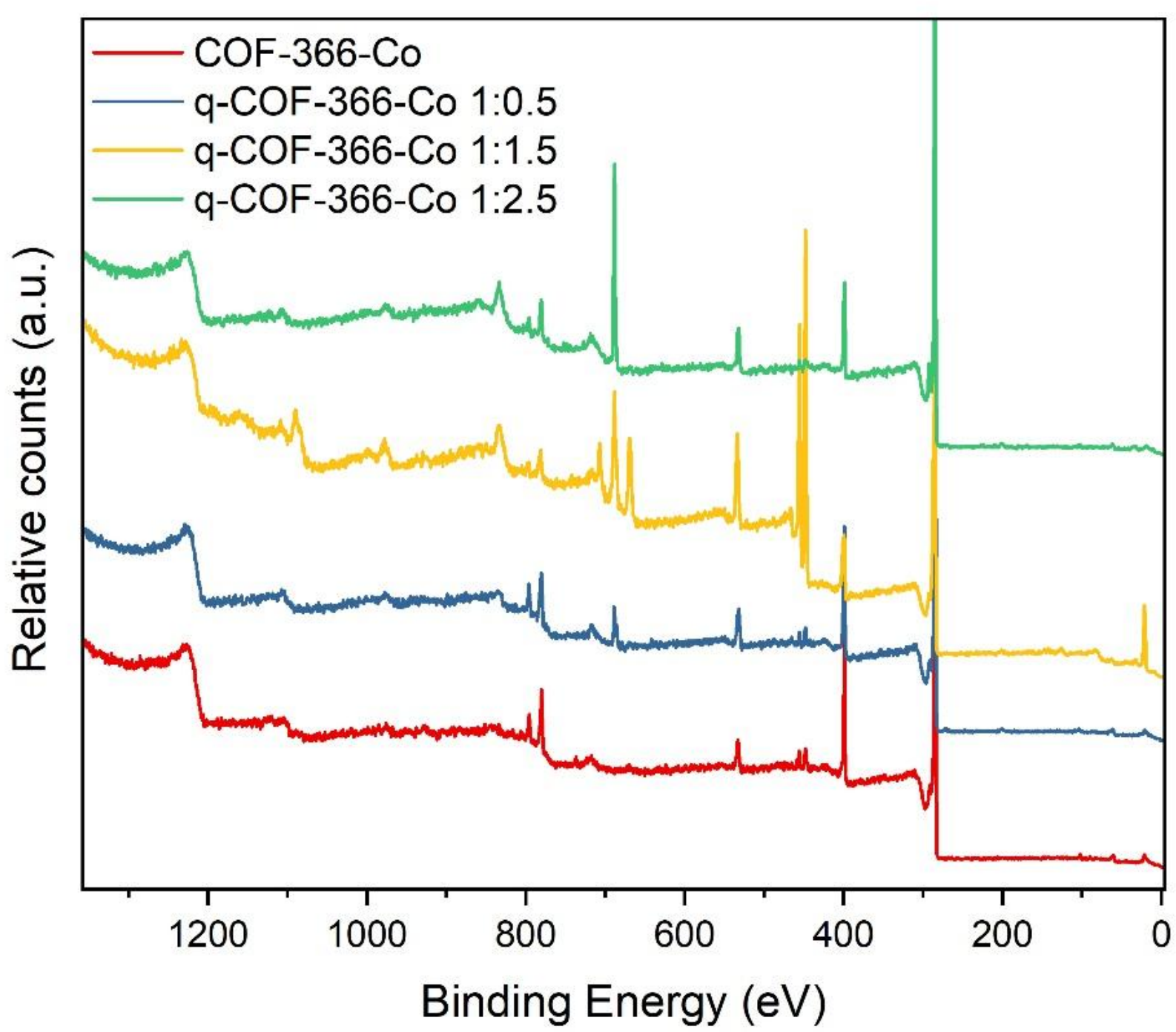

Supplementary Figure S2.4.2.1: XPS spectra of COF-366-Co and q-, r-, o- variants. The survey spectrum includes peaks corresponding to Co 2p, O 1s, C 1s, F 1s, N 1s, Cl 2p, C KLL, O KLL, and In. The presence of Indium is attributed to the In foil substrate used to support the powder samples.

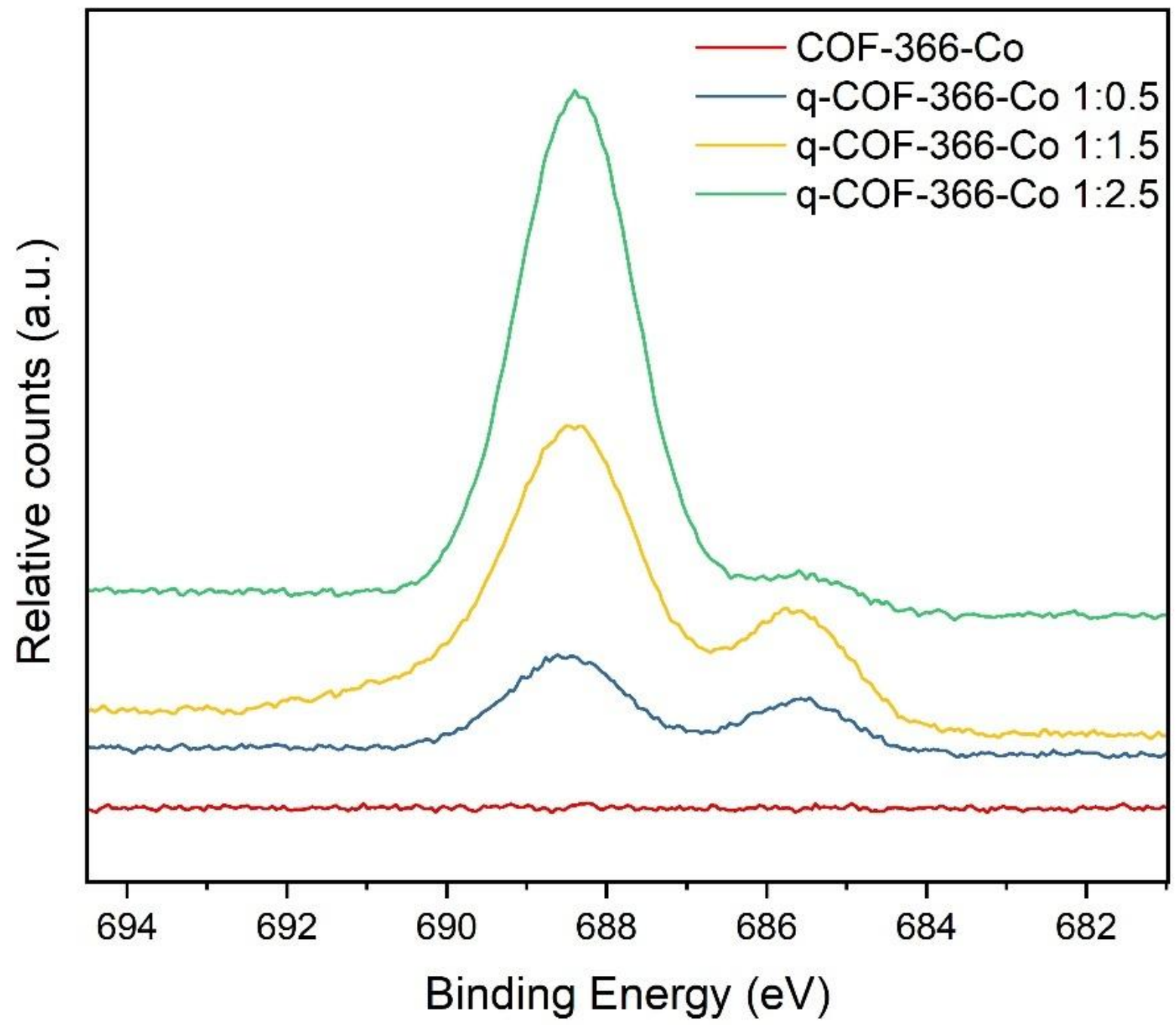

Supplementary Figure S2.4.2.2: XPS detail spectra of F1s of COF-366-Co and q-, r-, o- variants.

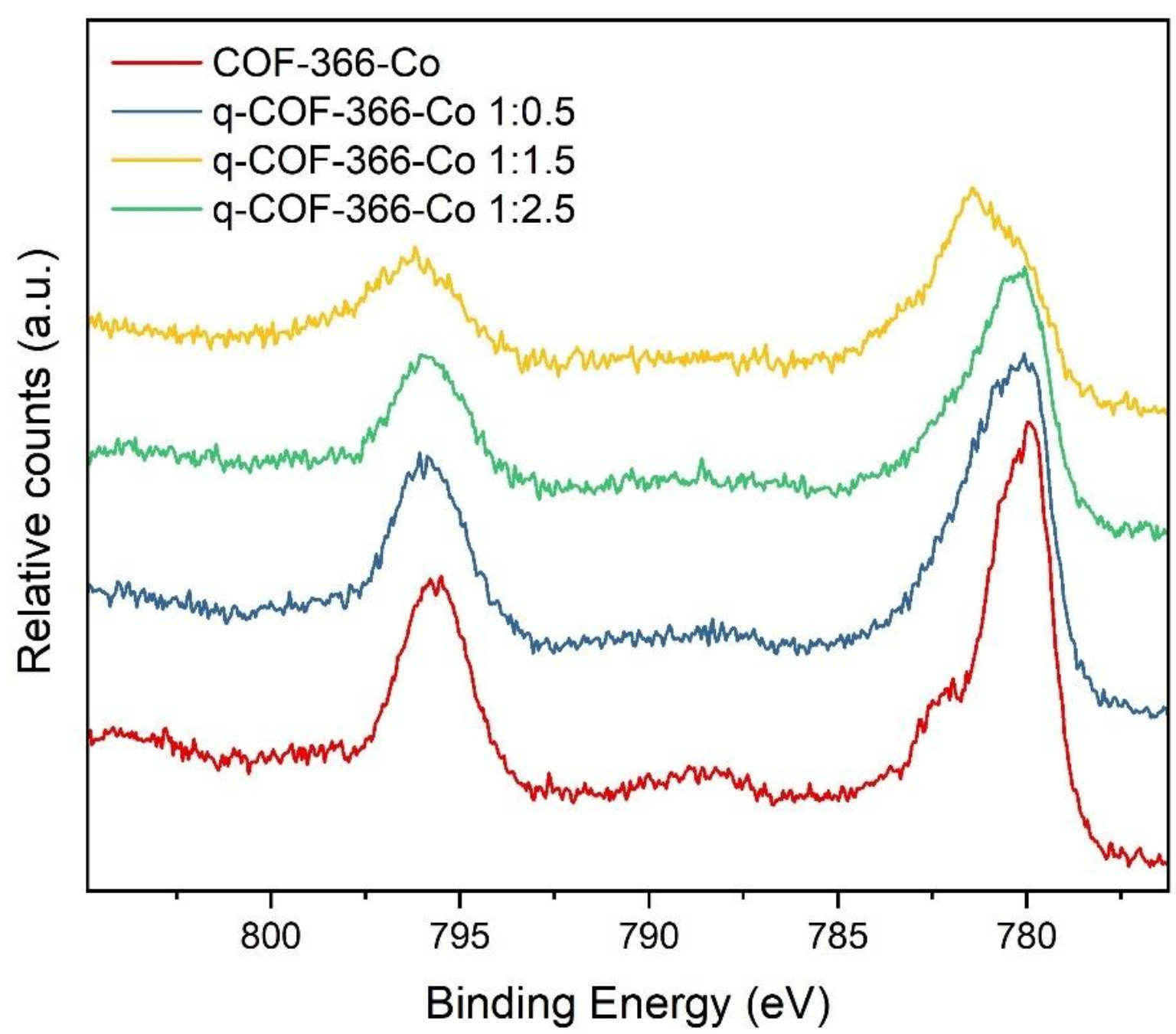

Supplementary Figure S2.4.2.3: XPS detail spectra of Co2p of COF-366-Co and q-, r-, o- variants.

S2.5 FTIR

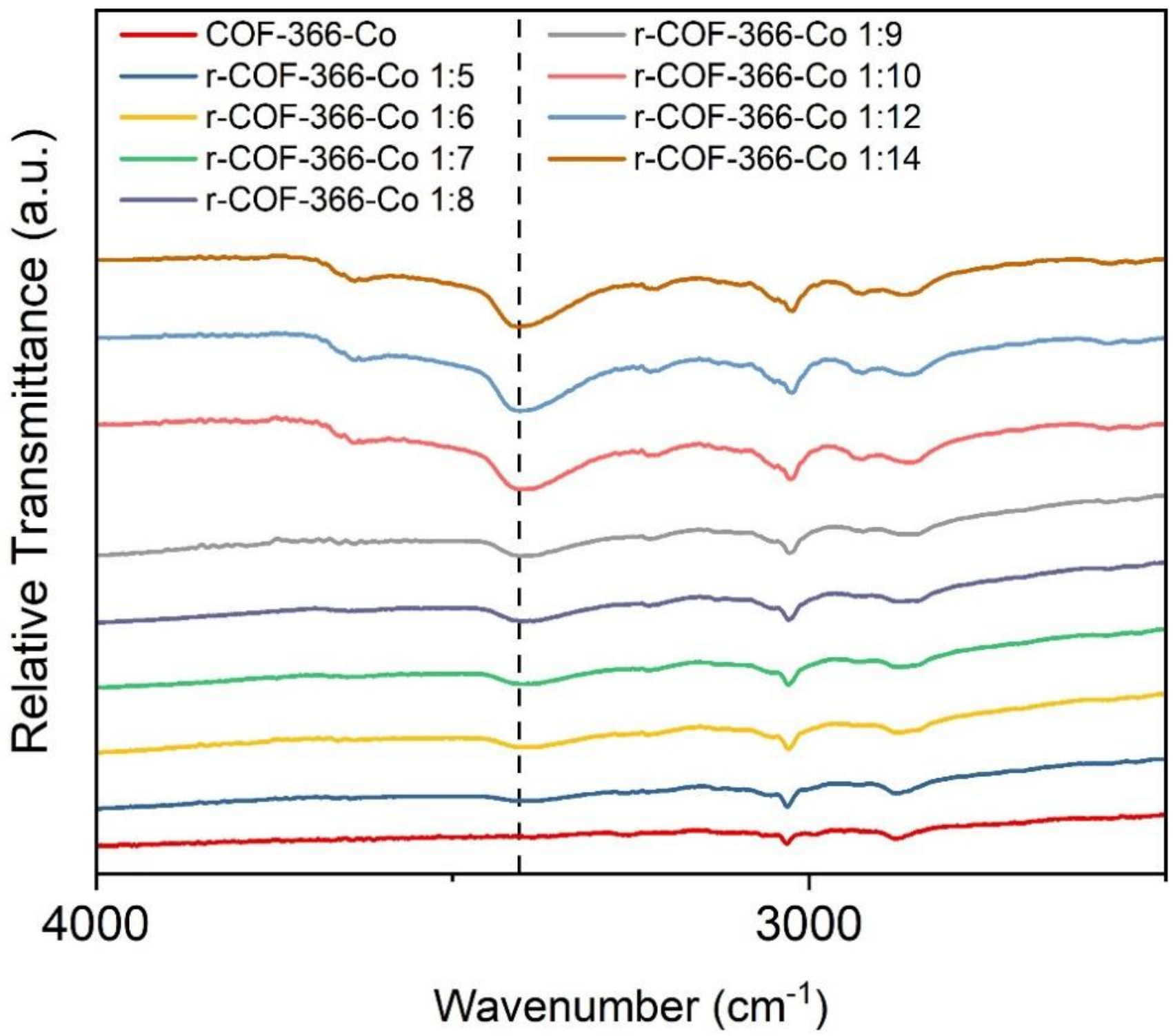

Supplementary Figure S2.5.1: High wavenumber FTIR of r-COF-366-Co functionalization series.

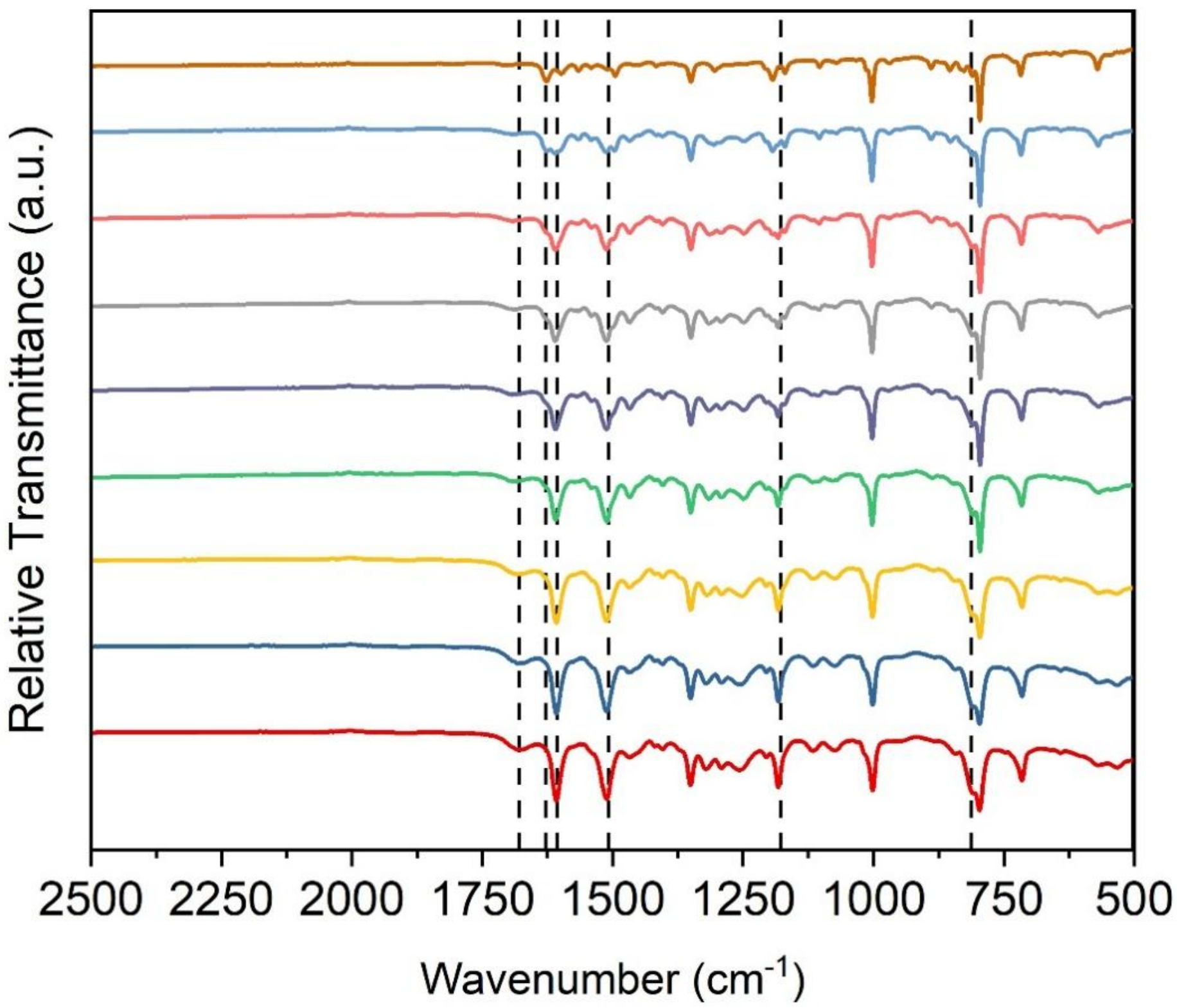

Supplementary Figure S2.5.2.: Low wavenumber FTIR of r-COF-366-Co functionalization series.

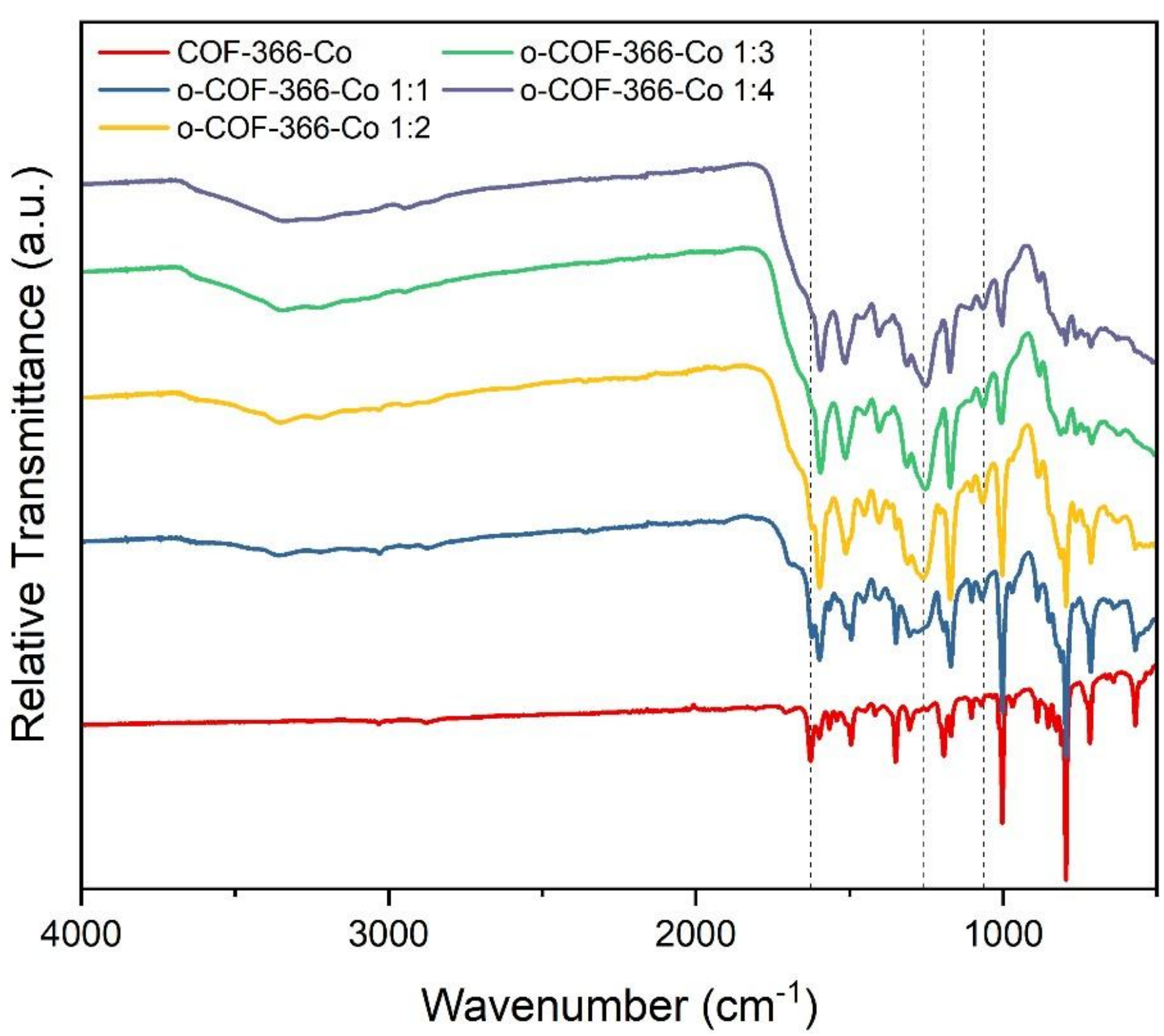

Supplementary Figure S2.5.3: FTIR of o-COF-366-Co functionalization series.

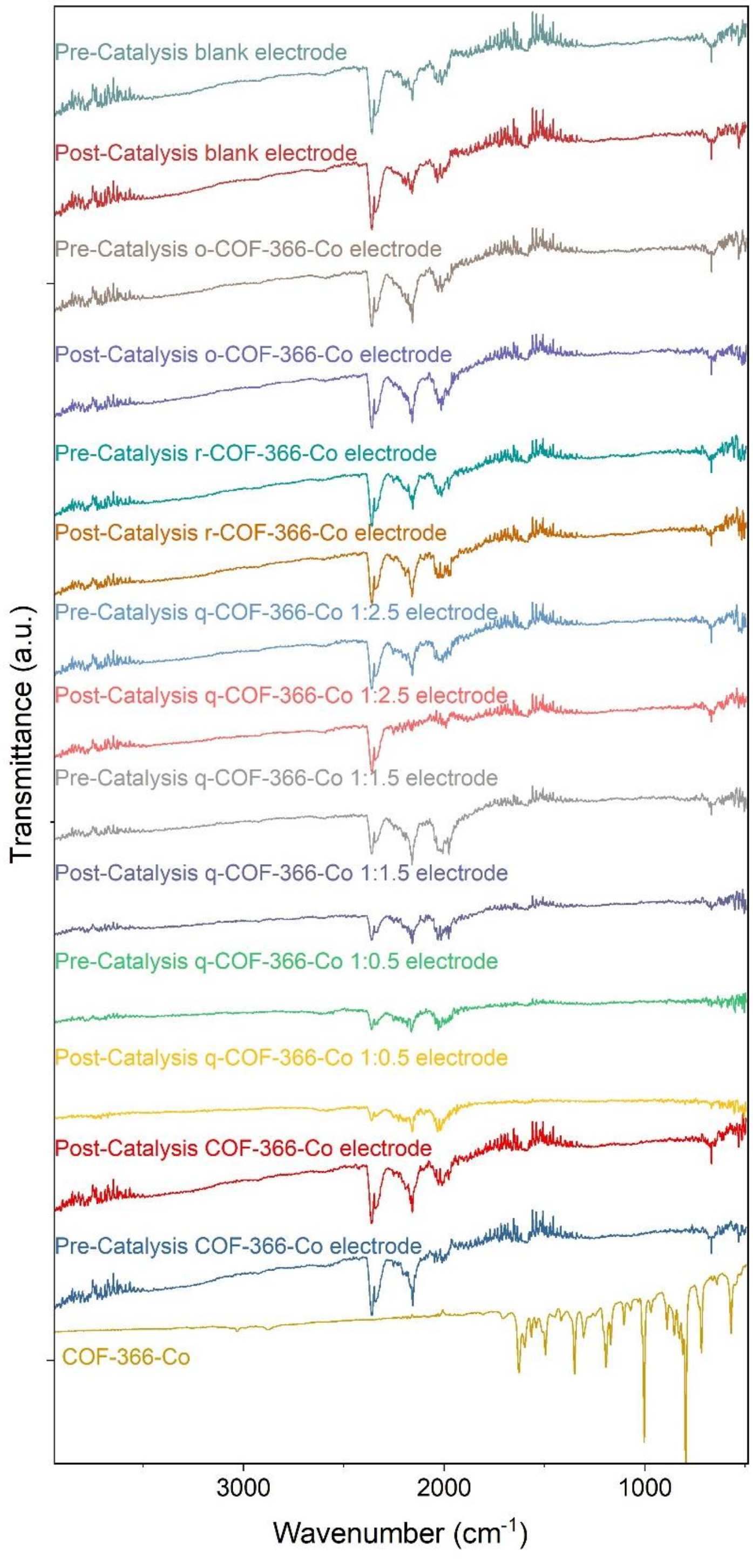

Supplementary Figure S2.5.4: FTIR of COF-366-Co compared to FTIR of electrodes pre- and post-catalysis of all materials with blank electrode

S2.6 Water Contact Angle measurements

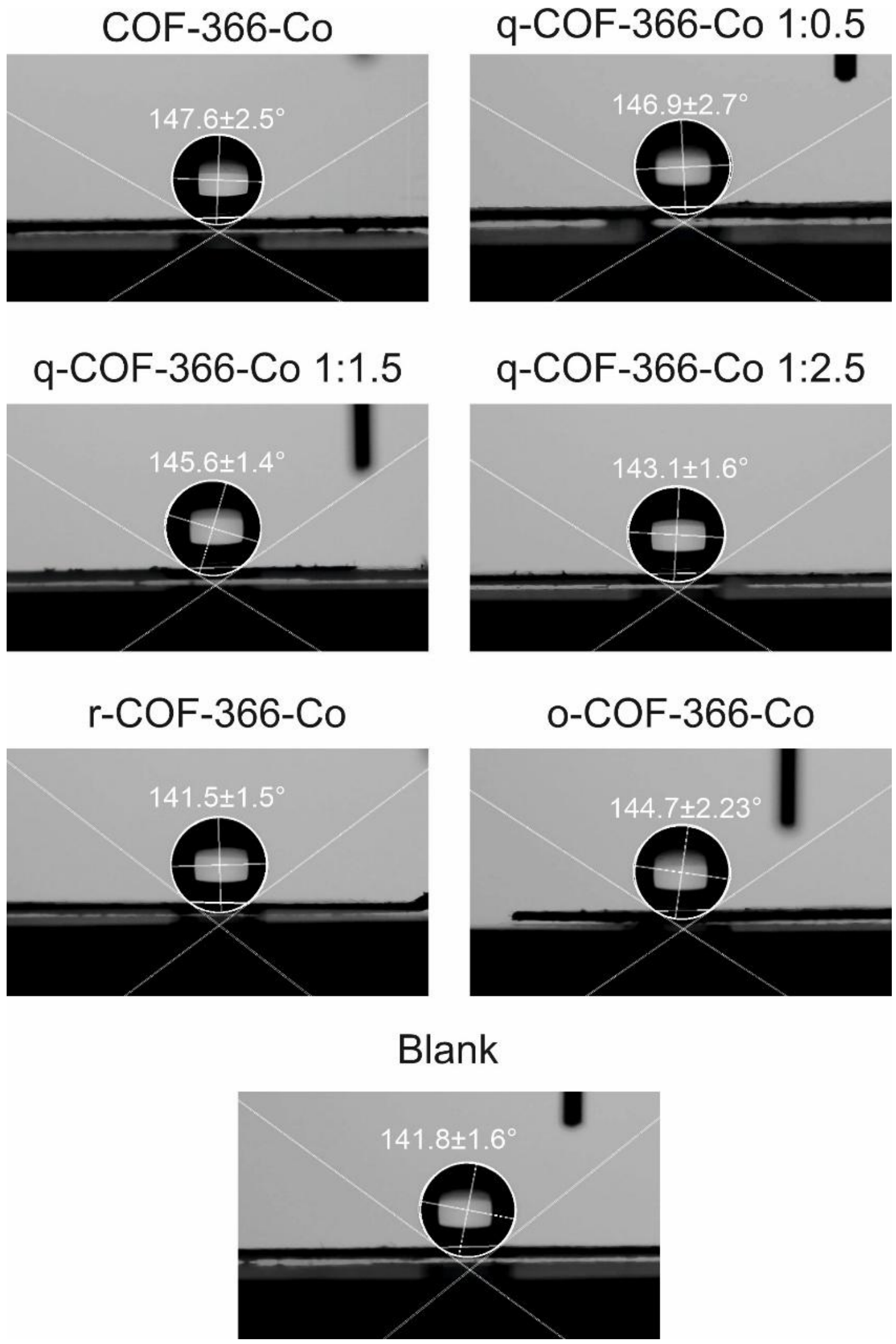

Supplementary Figure S2.6.1: Water contact angle measurements of prepared electrodes.

Material	Mean	Standard Deviation	Minimum	Median	Maximum
COF-366-Co	147.63333	2.50267	145.2	147.5	150.2
q-COF-366-Co 1:0.5	146.86667	2.70062	144.2	146.8	149.6
q-COF-366-Co 1:1.5	145.6	1.44222	144.4	145.2	147.2
q-COF-366-Co 1:2.5	143.06667	1.55027	141.3	143.7	144.2
r-COF-366-Co	141.46667	1.46401	139.9	141.7	142.8
o-COF-366-Co	144.7	2.25167	143.4	143.4	147.3
Blank	141.8	1.57162	140.1	142.1	143.2

Supplementary Table S2.6.1: Average water contact angle measurements on prepared electrodes from 3 measurements.

S2.7 Cobalt leaching test

Cobalt leaching from COF-366-Co under electrocatalytic reaction conditions (at -0.88 V vs RHE) was monitored by ICP-OES analysis of the electrolyte. The results can be found in the below table.

Material	Chronoamperometry time [h]	ICP-OES analysis [Co mg/L]	Co leaching [%]
COF-366-Co	3.5	0.008	0.8
COF-366-Co	3.5	0.021	1.2
COF-366-Co	17	0.199	11
q-COF-366-Co	3.5	0.021	2.3

Supplementary Table S2.7.1: ICP-OES results of Co amount in electrolyte and leaching %

S2.8 SEM/TEM Analysis

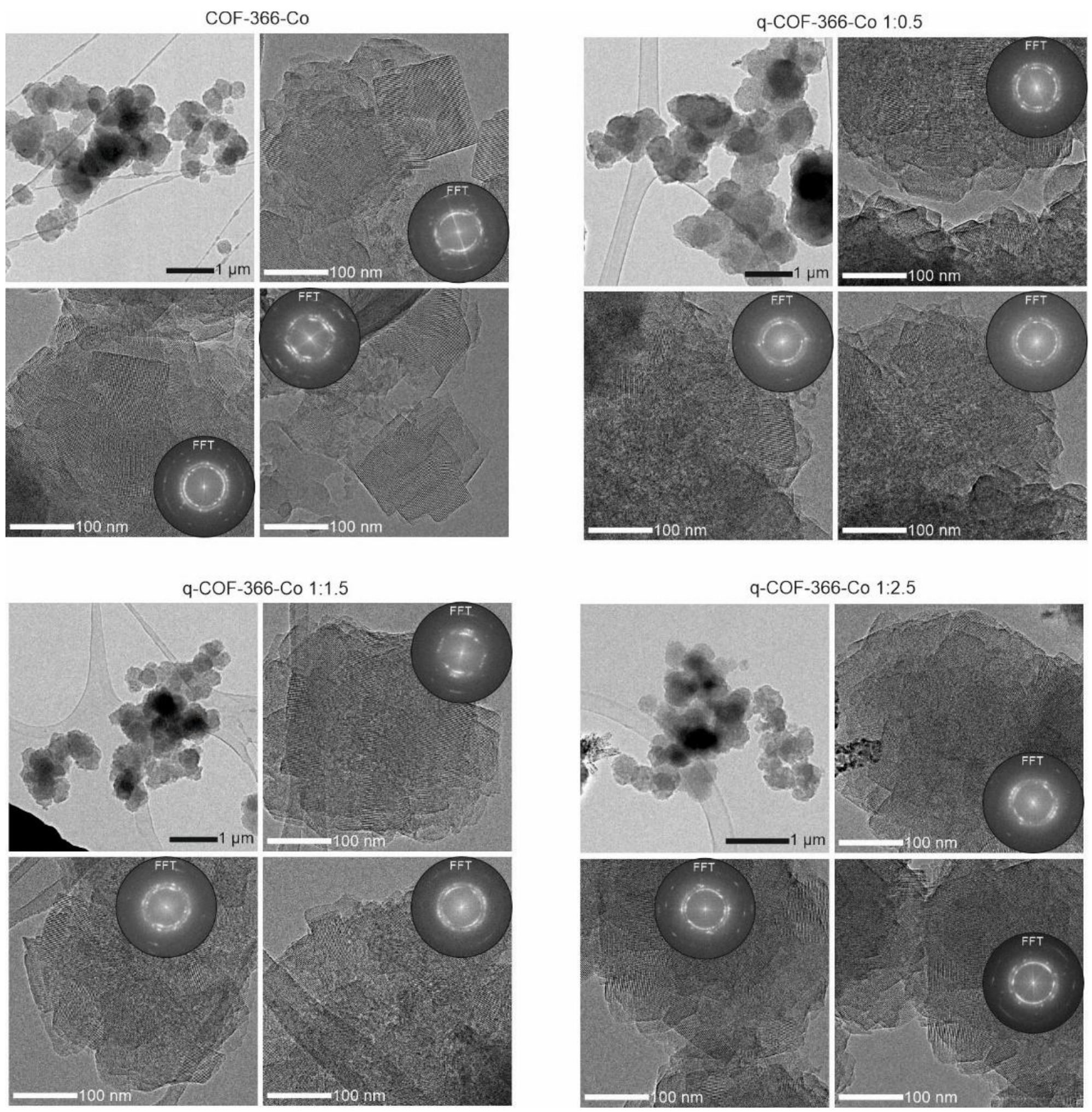

Supplementary Figure S2.8.1: TEM images of COF-366-Co, q-COF-366-Co 1:0.5, 1:1.5, and 1:2.5.

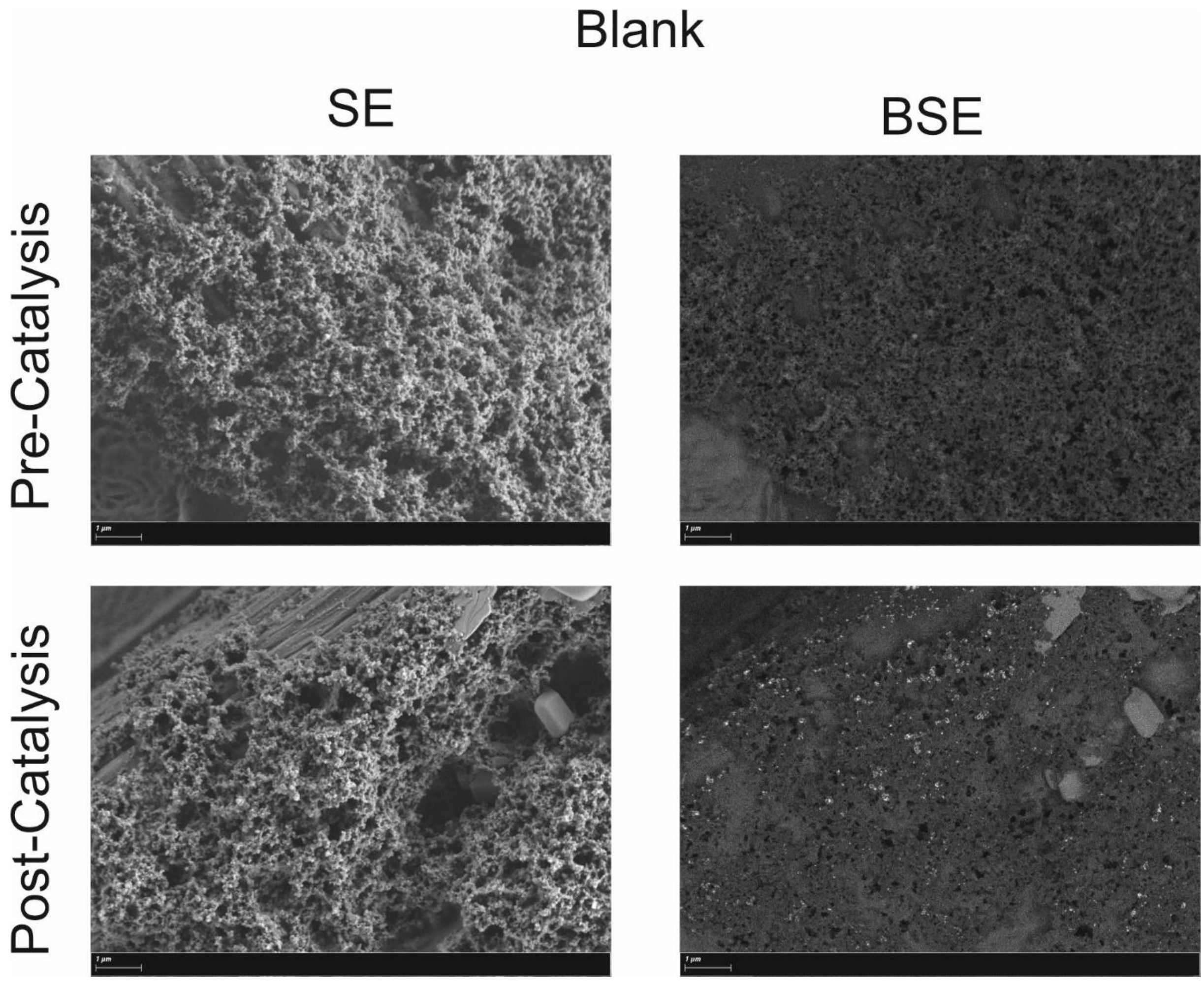

Supplementary Figure S2.8.2: SEM analysis of blank electrode pre- (top) and post- (bottom) catalysis with visualization of secondary electrons (left) and backscattered electrons (right).

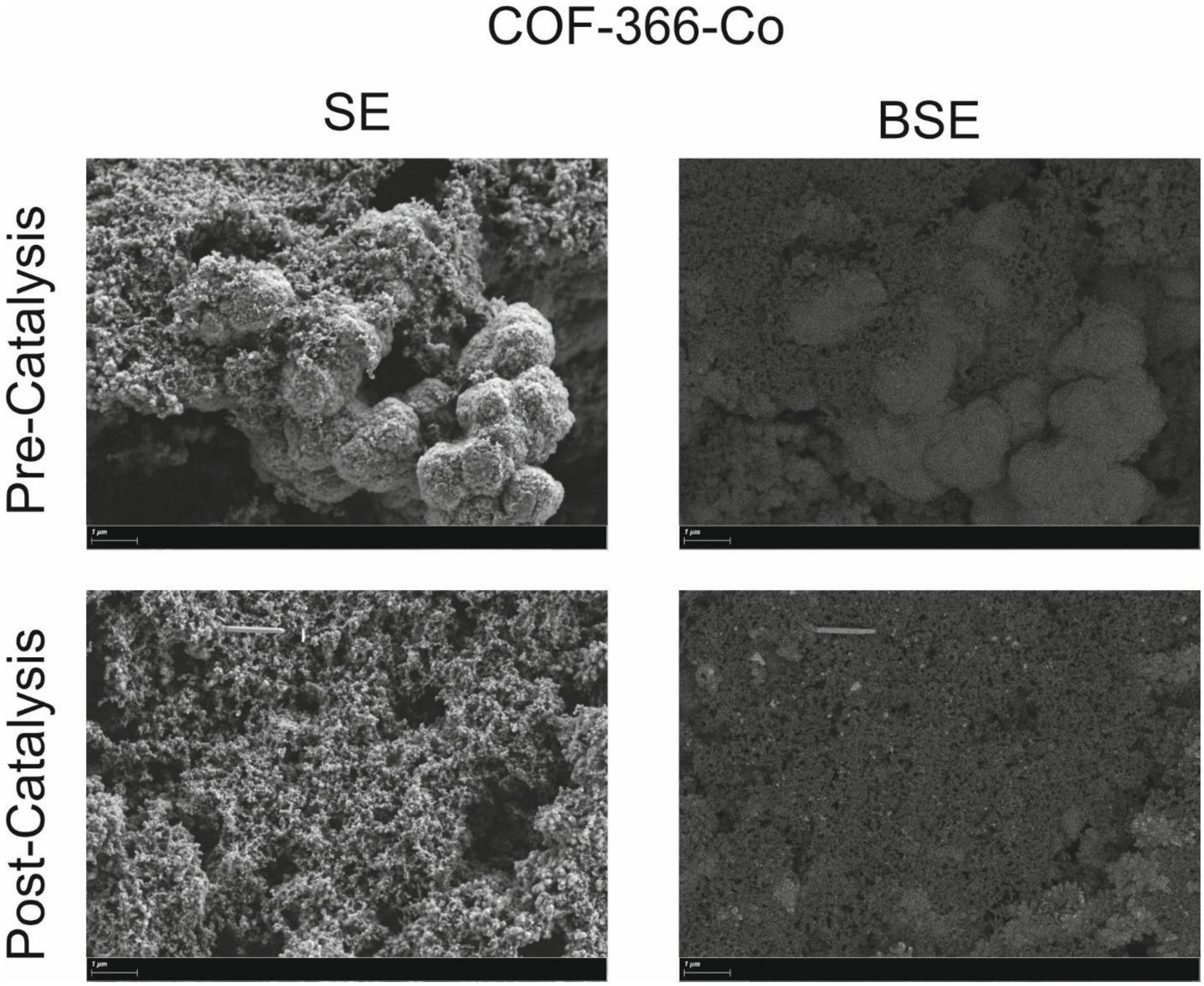

Supplementary Figure S2.8.3: SEM analysis of COF-366-Co electrode pre- (top) and post- (bottom) catalysis with visualization of secondary electrons (left) and backscattered electrons (right).

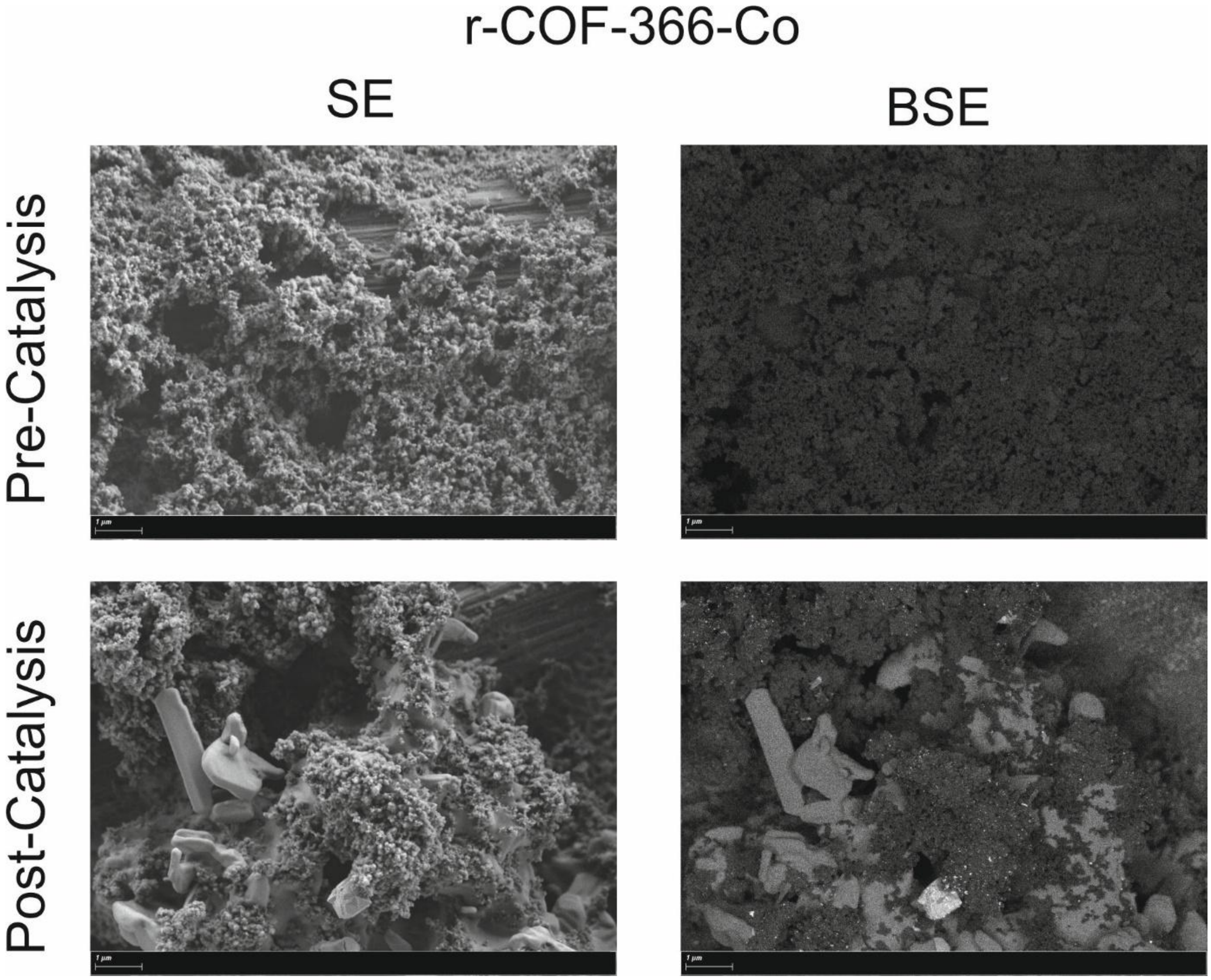

Supplementary Figure S2.8.4: SEM analysis of r-COF-366-Co electrode pre- (top) and post- (bottom) catalysis with visualization of secondary electrons (left) and backscattered electrons (right).

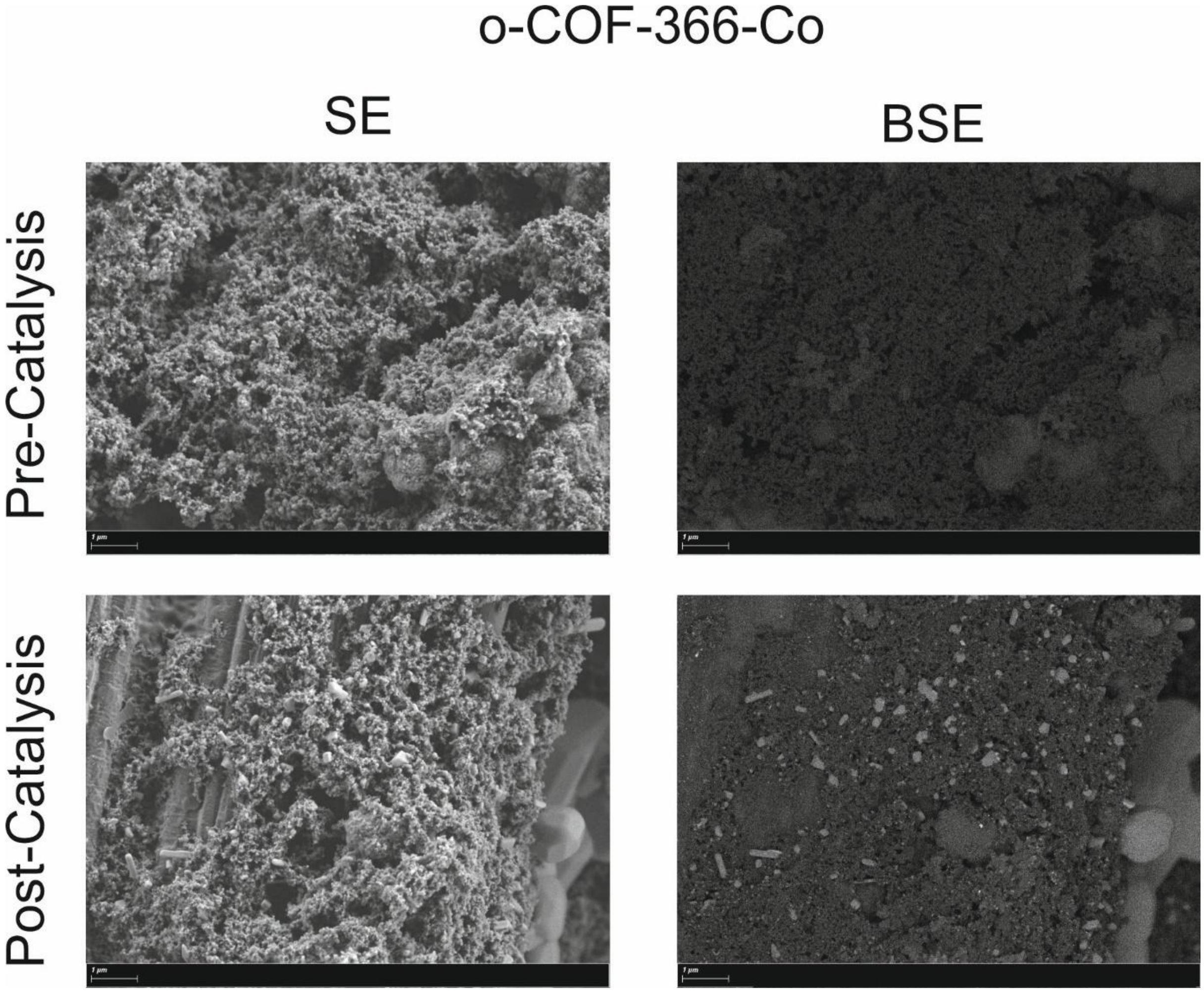

Supplementary Figure S2.8.5: SEM analysis of q-COF-366-Co electrode pre- (top) and post- (bottom) catalysis with visualization of secondary electrons (left) and backscattered electrons (right).

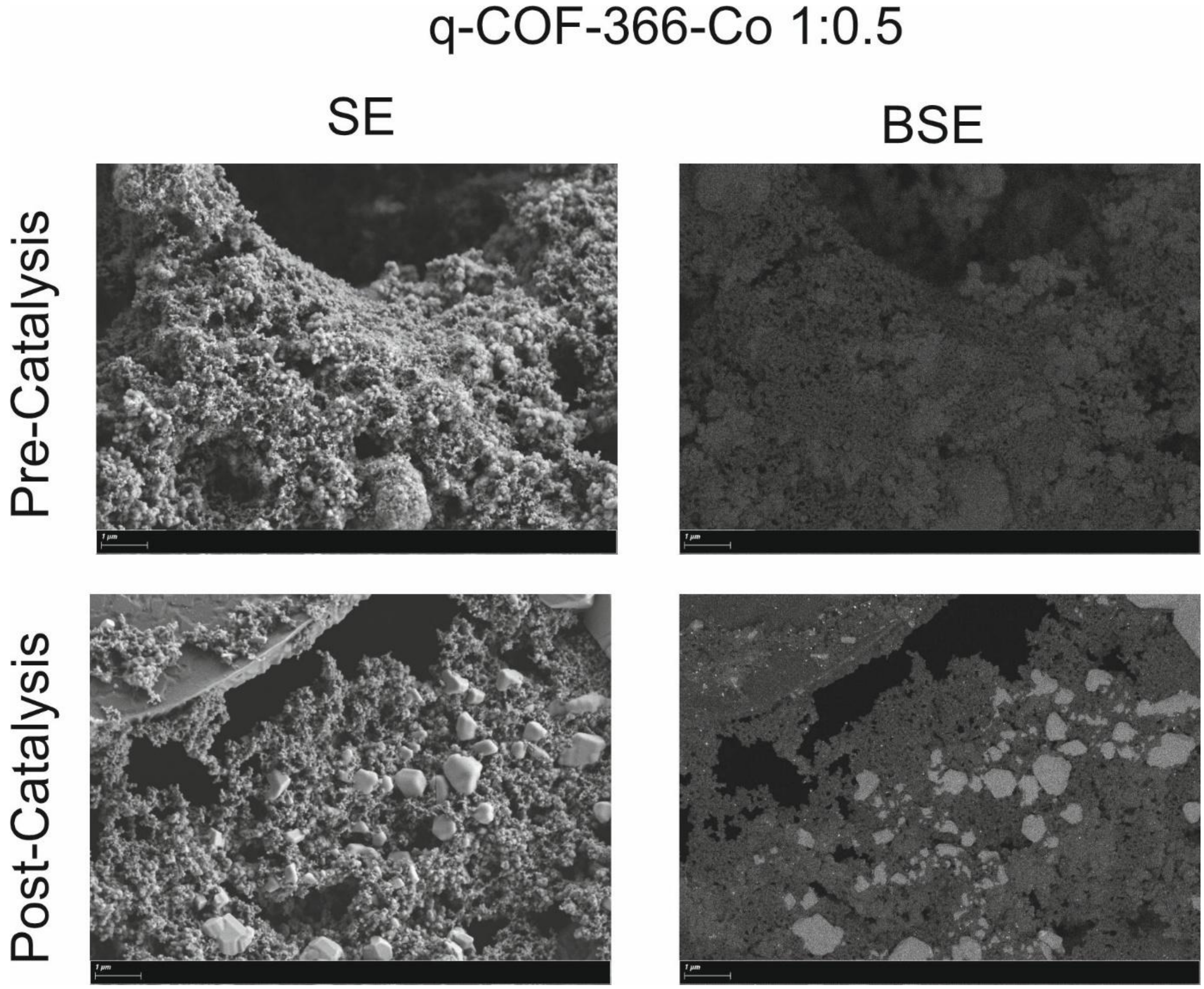

Supplementary Figure S2.8.6: SEM analysis of q-COF-366-Co 1:0.5 electrode pre- (top) and post- (bottom) catalysis with visualization of secondary electrons (left) and backscattered electrons (right).

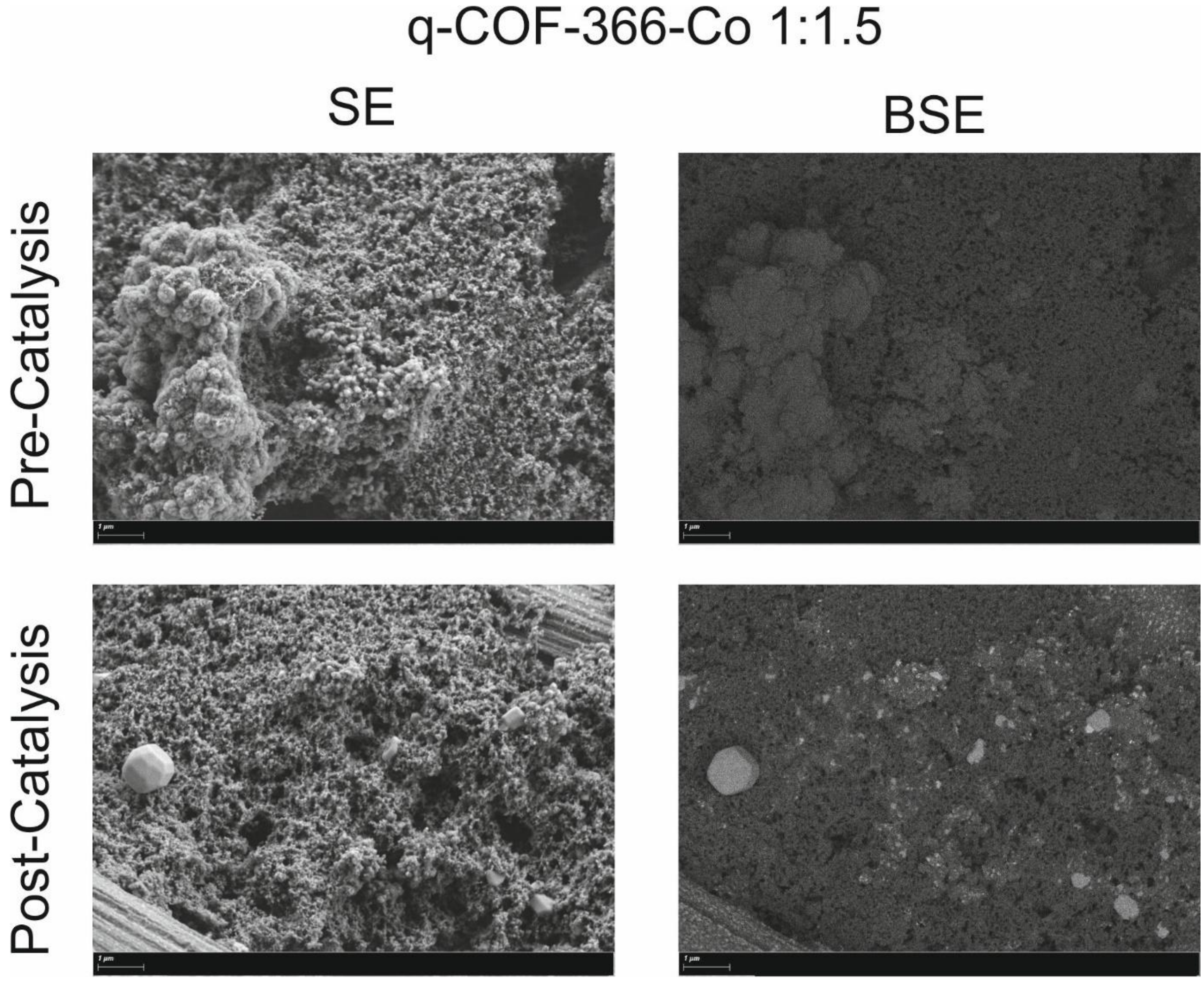

Supplementary Figure S2.8.7: SEM analysis of q-COF-366-Co 1:1.5 electrode pre- (top) and post- (bottom) catalysis with visualization of secondary electrons (left) and backscattered electrons (right).

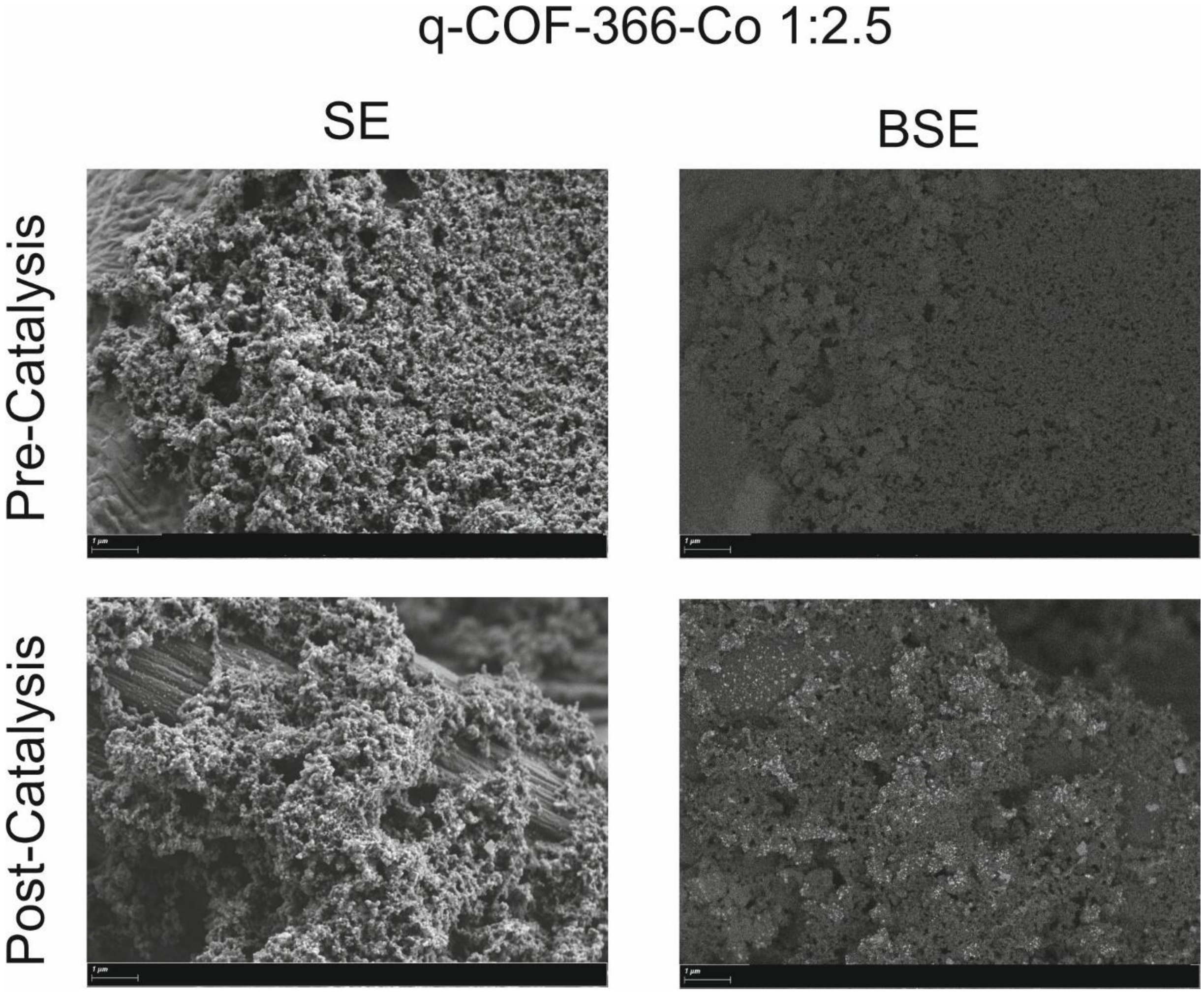

Supplementary Figure S2.8.8: SEM analysis of q-COF-366-Co 1:2.5 electrode pre- (top) and post- (bottom) catalysis with visualization of secondary electrons (left) and backscattered electrons (right).

COF-366-Co electrode post catalysis

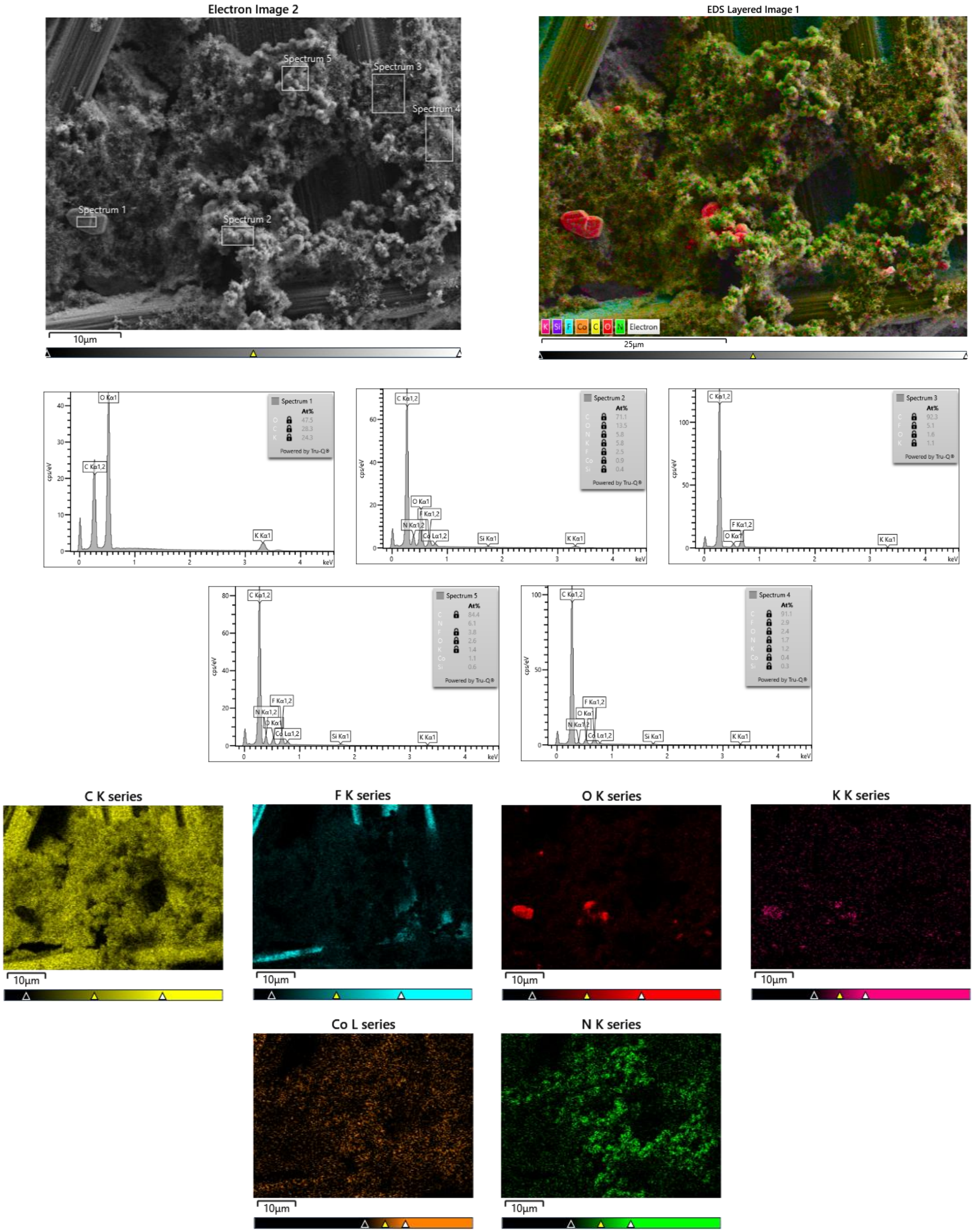

Supplementary Figure S2.8.9: EDX Analysis of COF-366-Co electrode post-catalysis with elemental mapping.

COF-366-Co electrode pre-catalysis

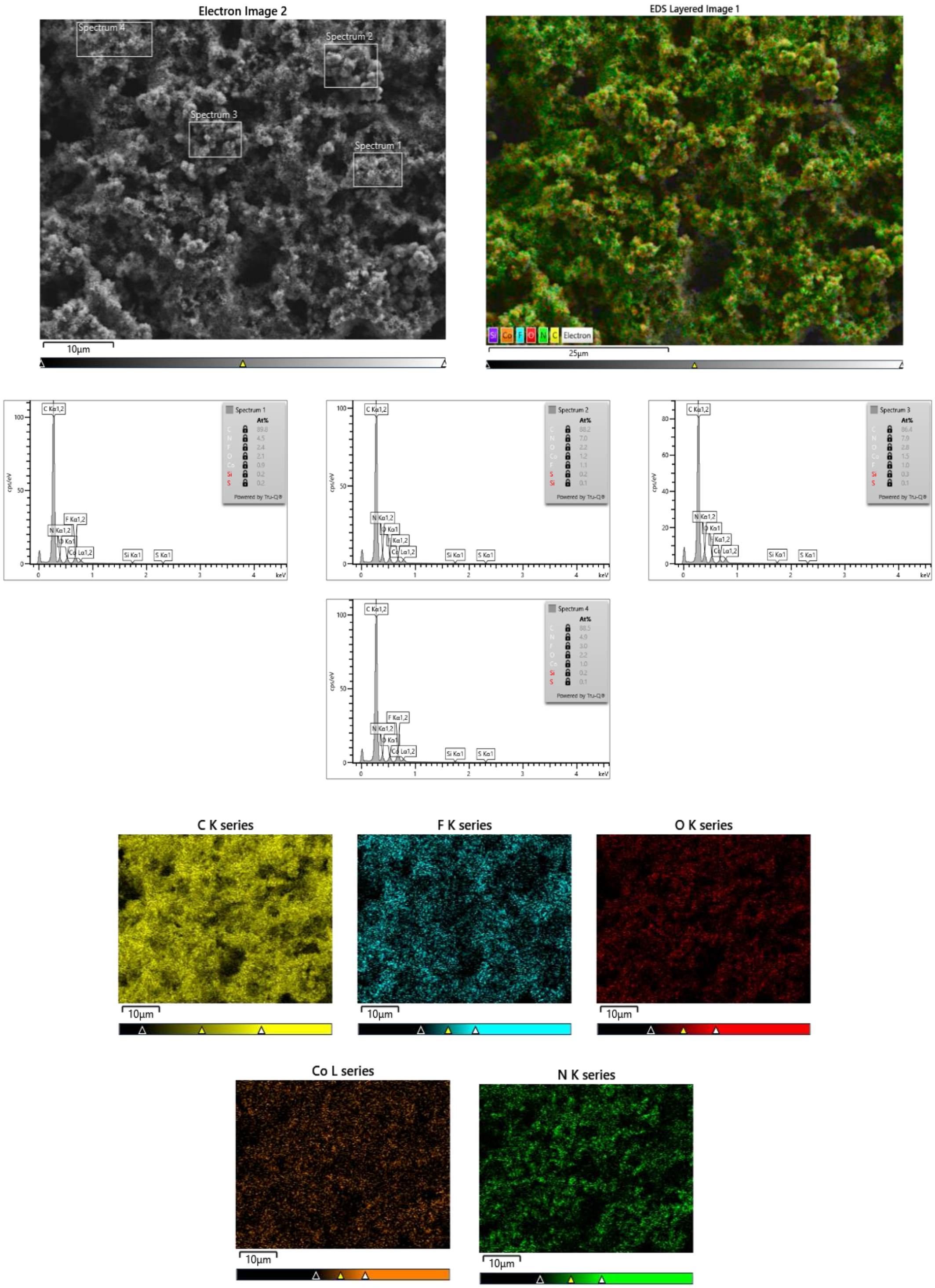

Supplementary Figure S2.8.10: EDX Analysis of COF-366-Co electrode pre-catalysis with elemental mapping.

q-COF-366-Co electrode 1:0.5 post-catalysis

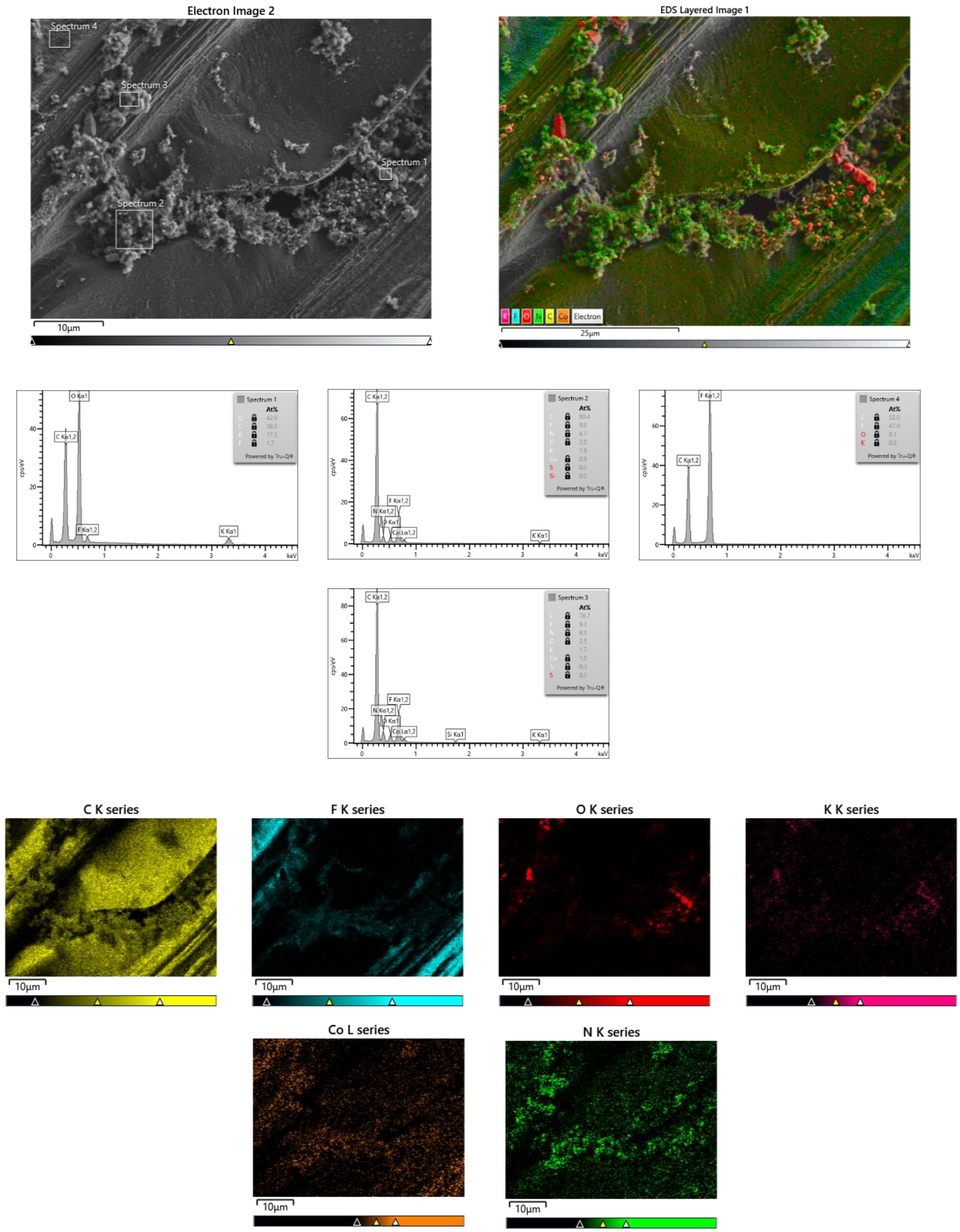

Supplementary Figure S2.8.11: EDX Analysis of q-COF-366-Co 1:0.5 electrode post-catalysis with elemental mapping.

q-COF-366-Co electrode 1:0.5 pre-catalysis

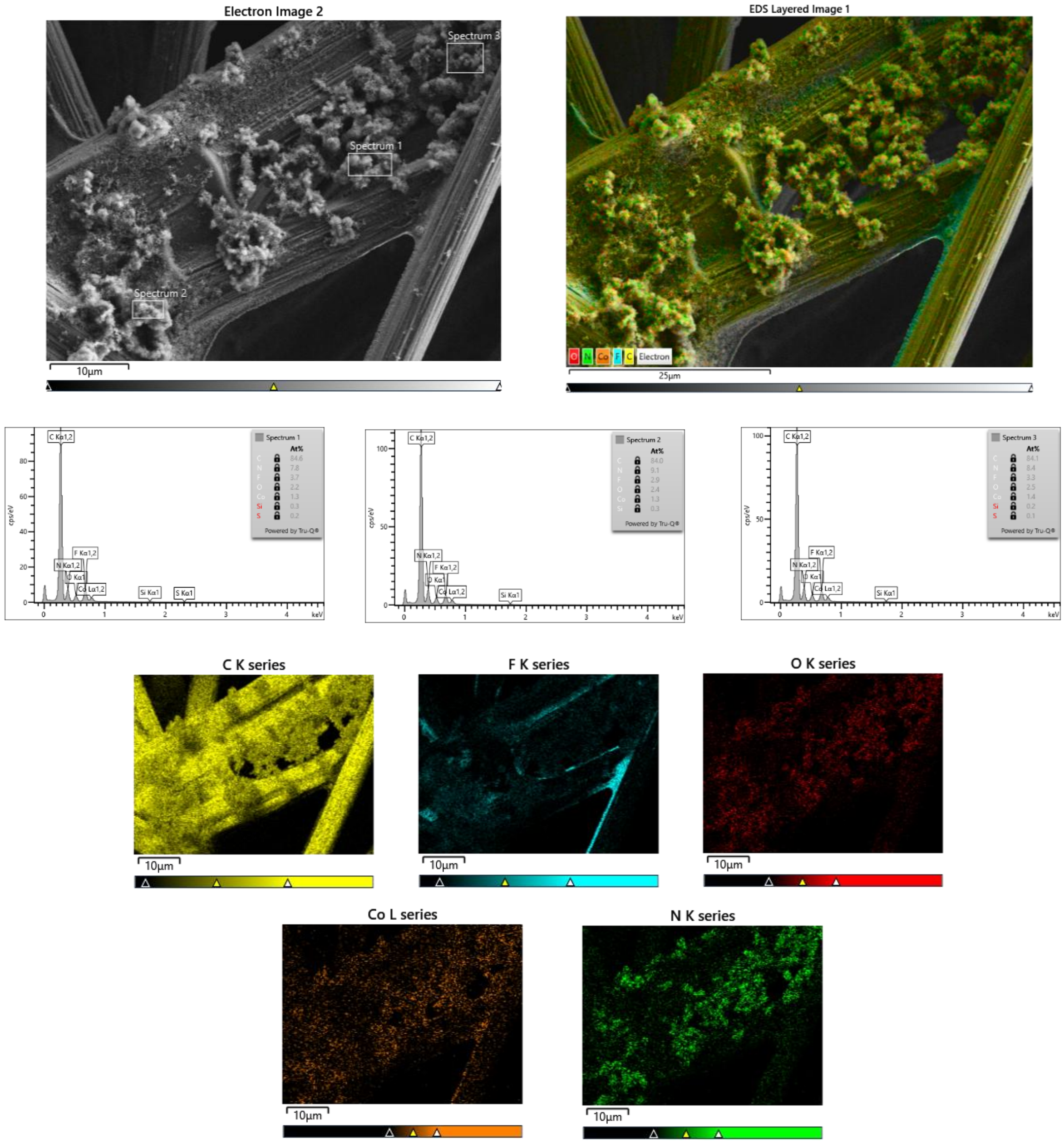

Supplementary Figure S2.8.12: EDX Analysis of q-COF-366-Co 1:0.5 electrode pre-catalysis with elemental mapping.

q-COF-366-Co electrode 1:1.5 post-catalysis

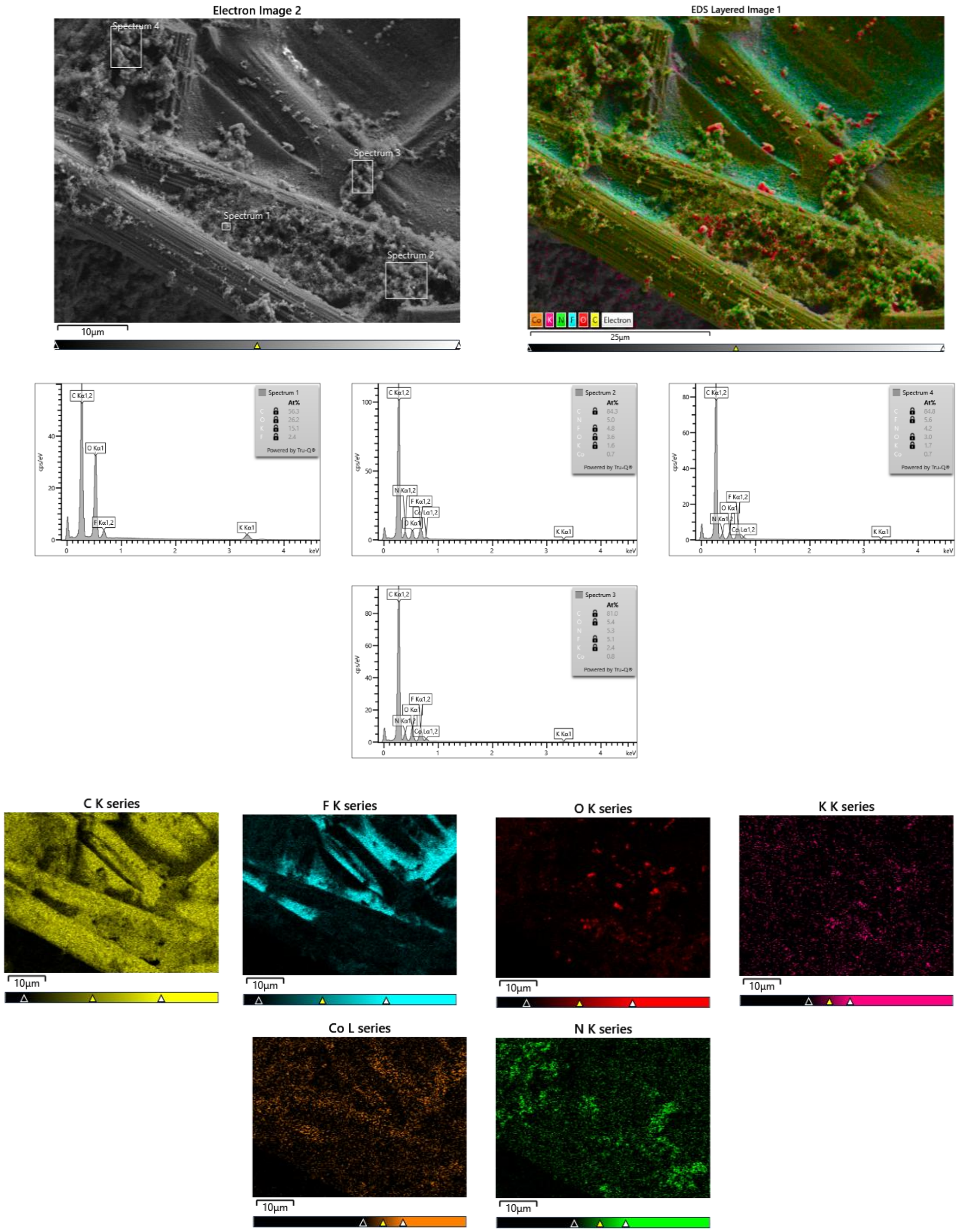

Supplementary Figure S2.8.13: EDX Analysis of q-COF-366-Co 1:1.5 electrode post-catalysis with elemental mapping.

q-COF-366-Co electrode 1:2.5 post-catalysis

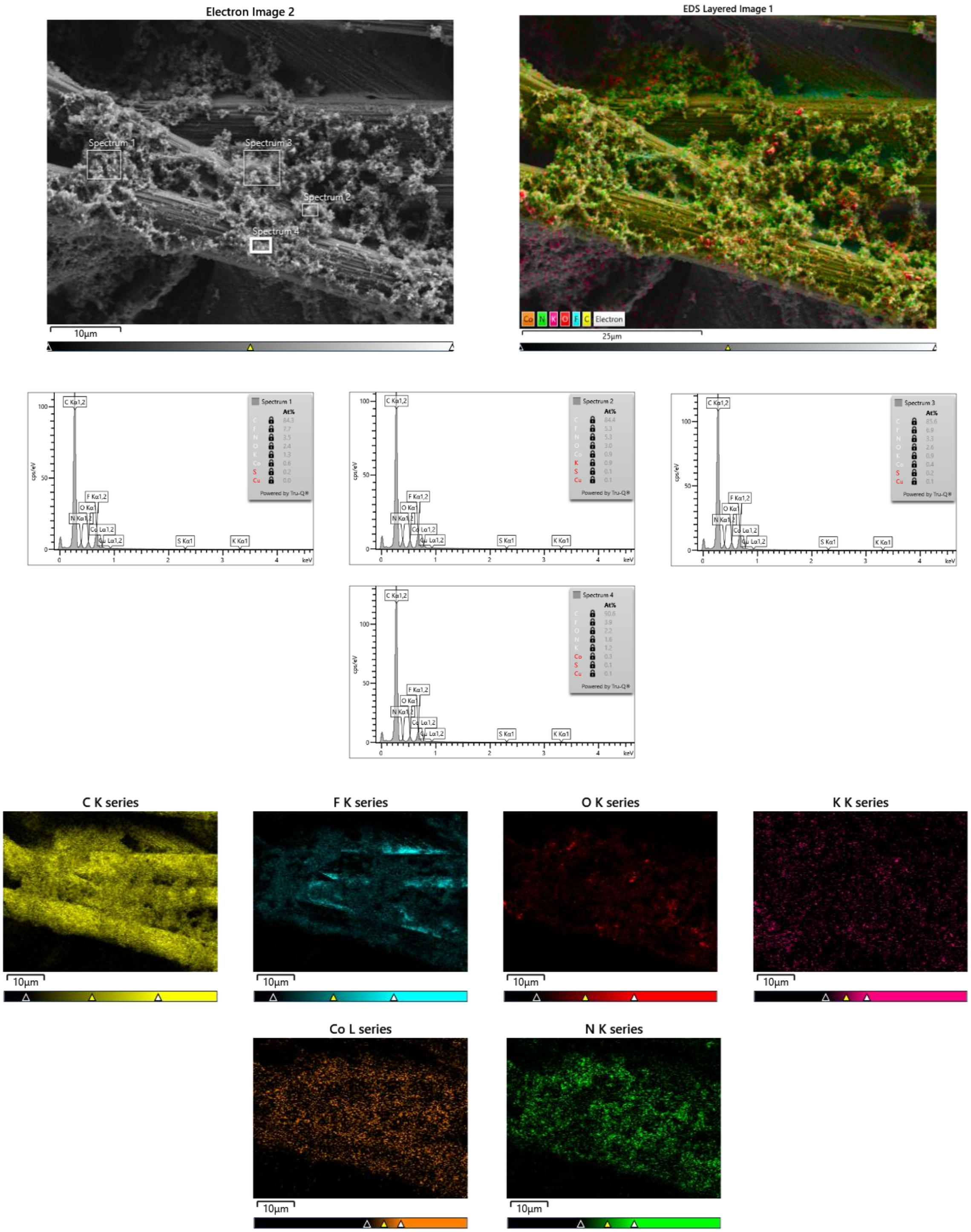

Supplementary Figure S2.8.14: EDX Analysis of q-COF-366-Co 1:2.5 electrode post-catalysis with elemental mapping.

q-COF-366-Co electrode 1:2.5 pre-catalysis

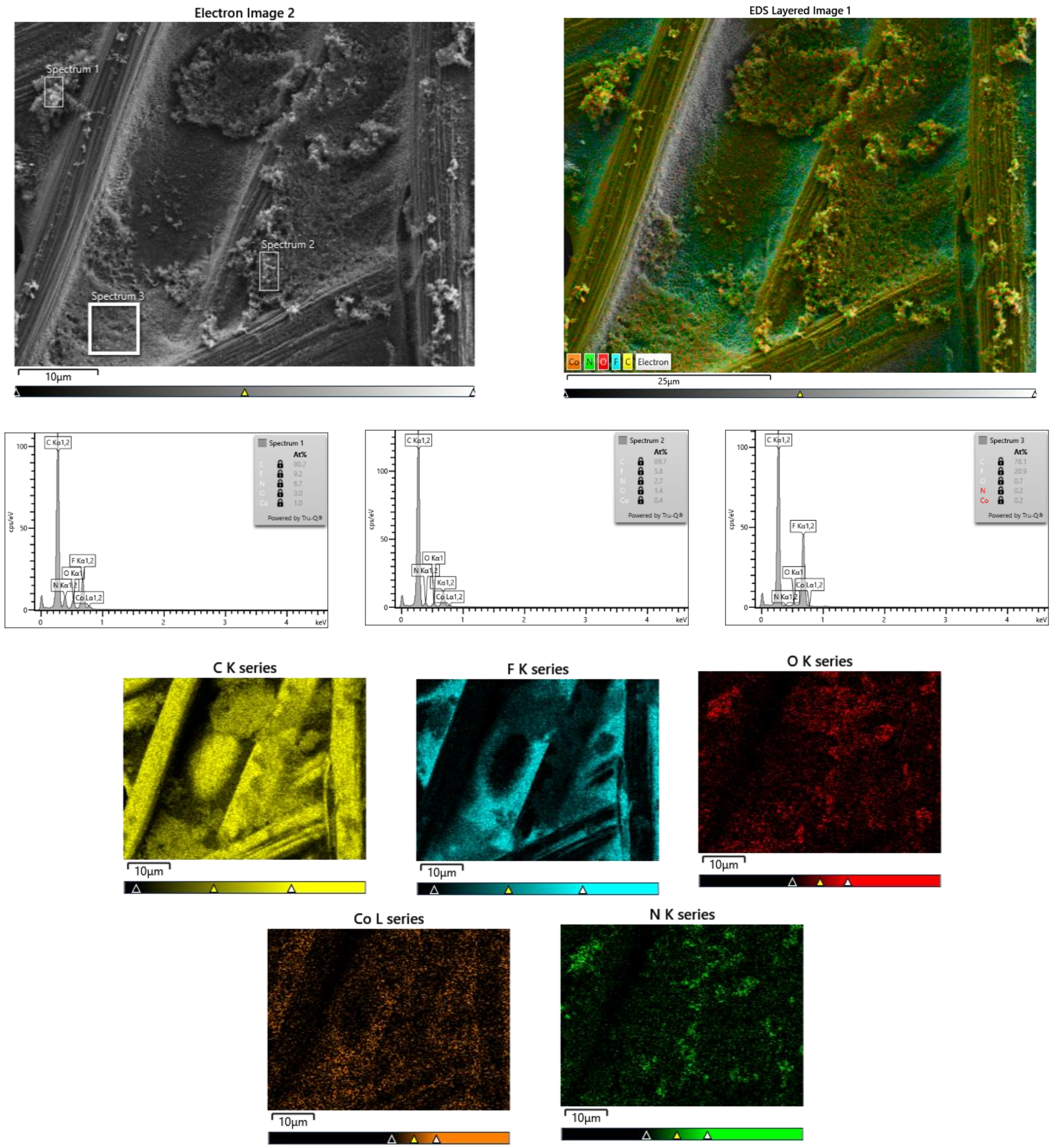

Supplementary Figure S2.8.15: EDX Analysis of q-COF-366-Co 1:2.5 electrode pre-catalysis with elemental mapping.

r-COF-366-Co electrode post-catalysis

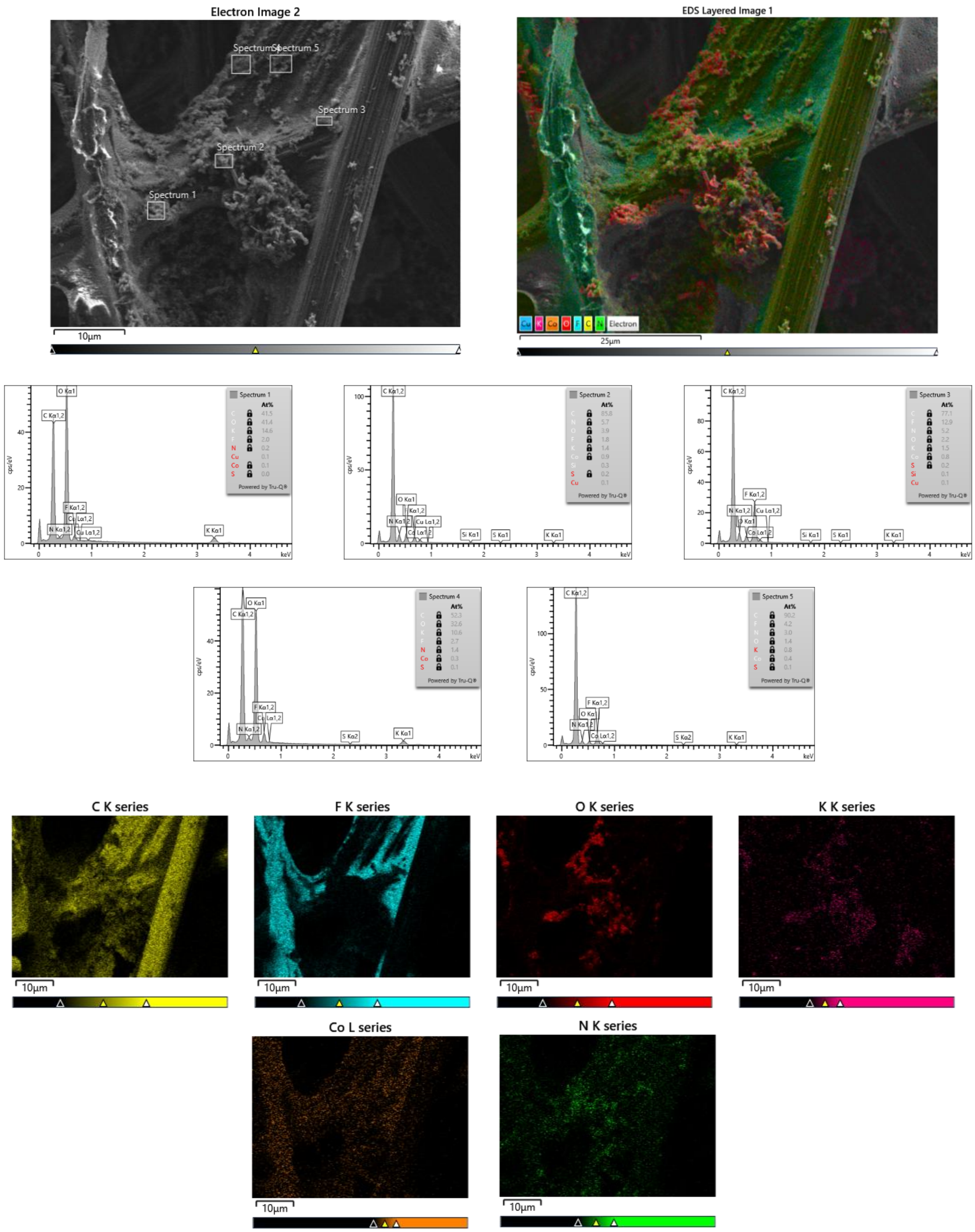

Supplementary Figure S2.8.16: EDX Analysis of r-COF-366-Co electrode post-catalysis with elemental mapping.

r-COF-366-Co electrode pre-catalysis

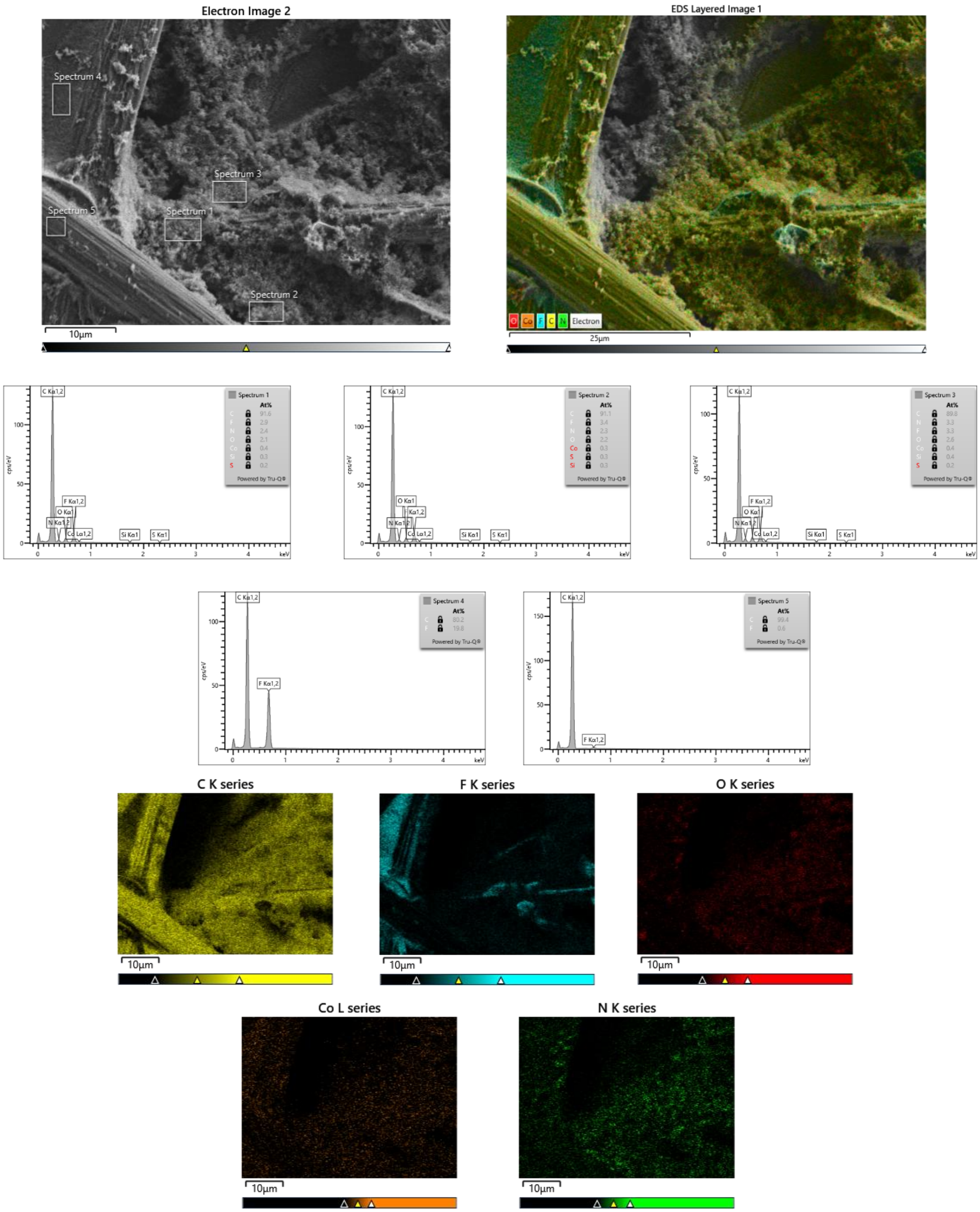

Supplementary Figure S2.8.17: EDX Analysis of r-COF-366-Co electrode pre-catalysis with elemental mapping.

o-COF-366-Co electrode post-catalysis

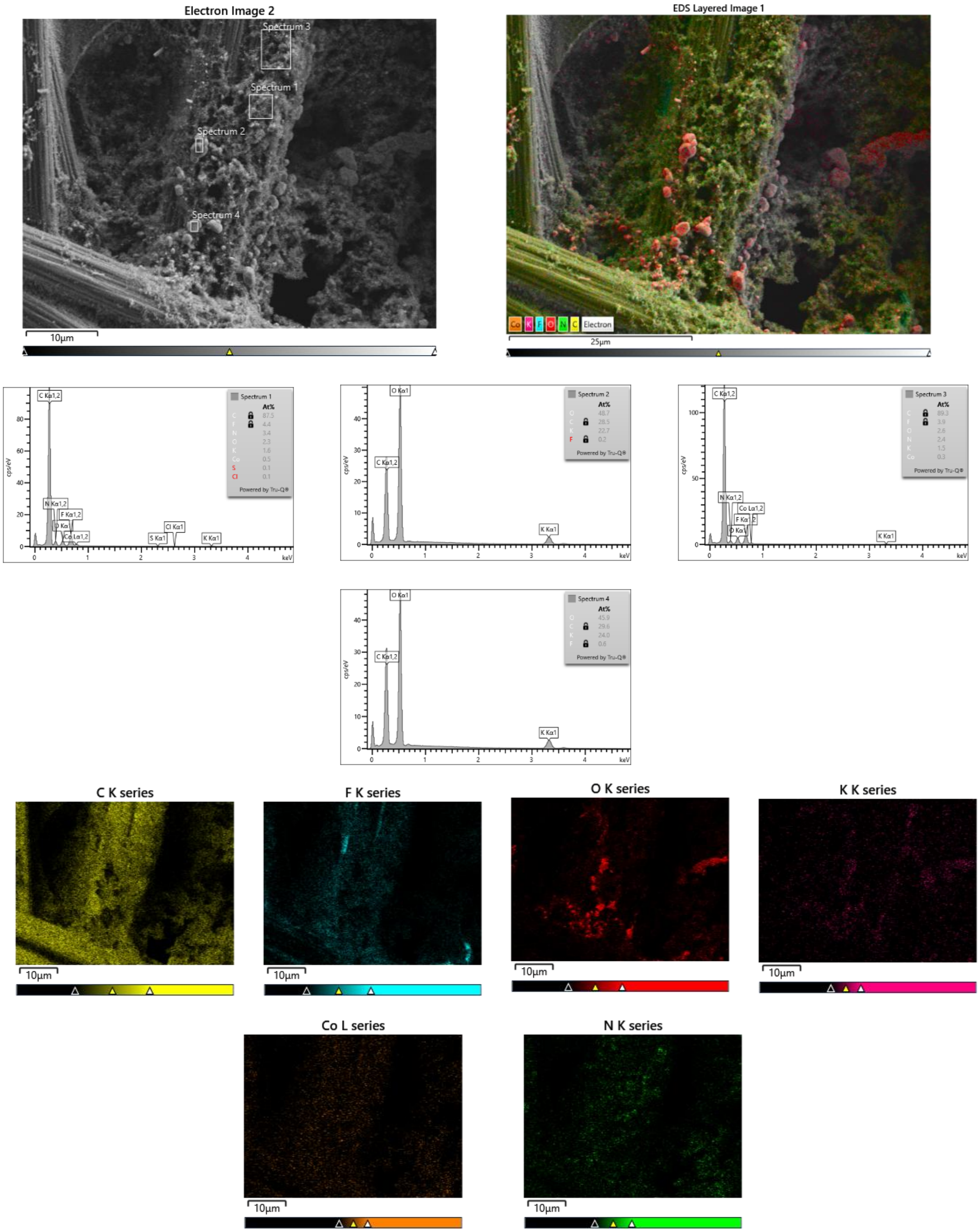

Supplementary Figure S2.8.18: EDX Analysis of o-COF-366-Co electrode post-catalysis with elemental mapping.

o-COF-366-Co electrode pre-catalysis

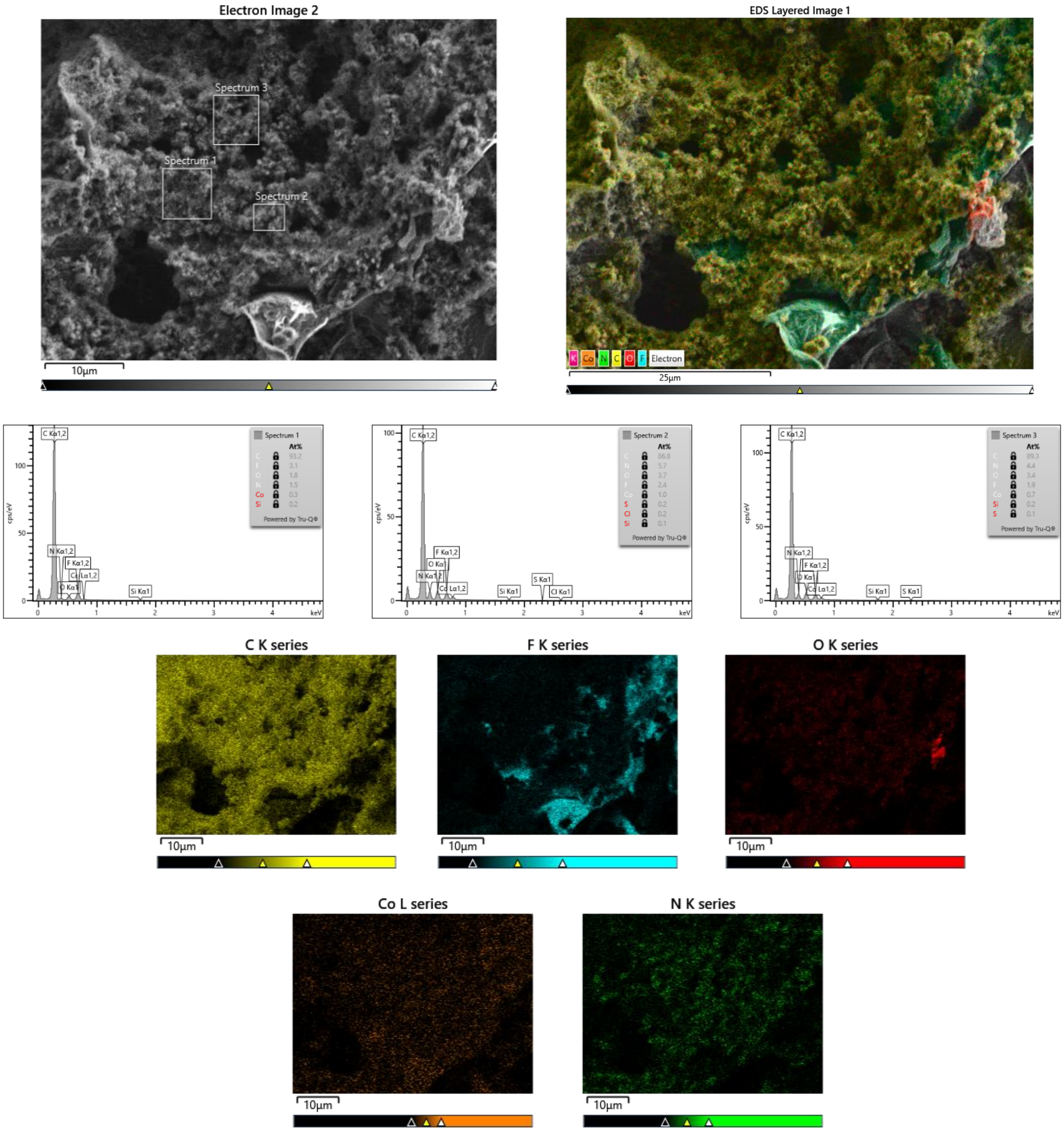

Supplementary Figure S2.8.19: EDX Analysis of o-COF-366-Co electrode pre-catalysis with elemental mapping.

Blank electrode post-catalysis

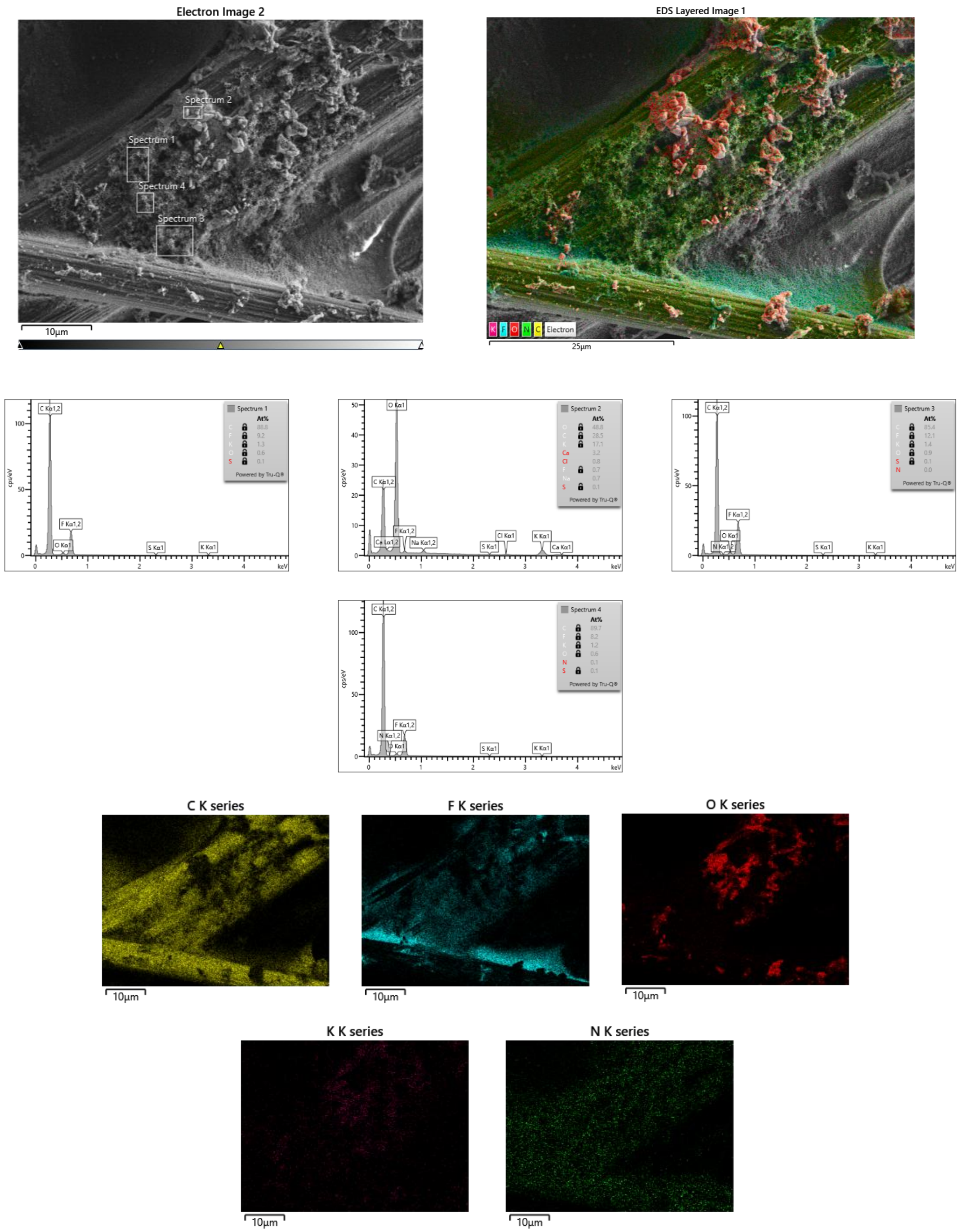

Supplementary Figure S2.8.20: EDX Analysis of blank electrode post-catalysis with elemental mapping.

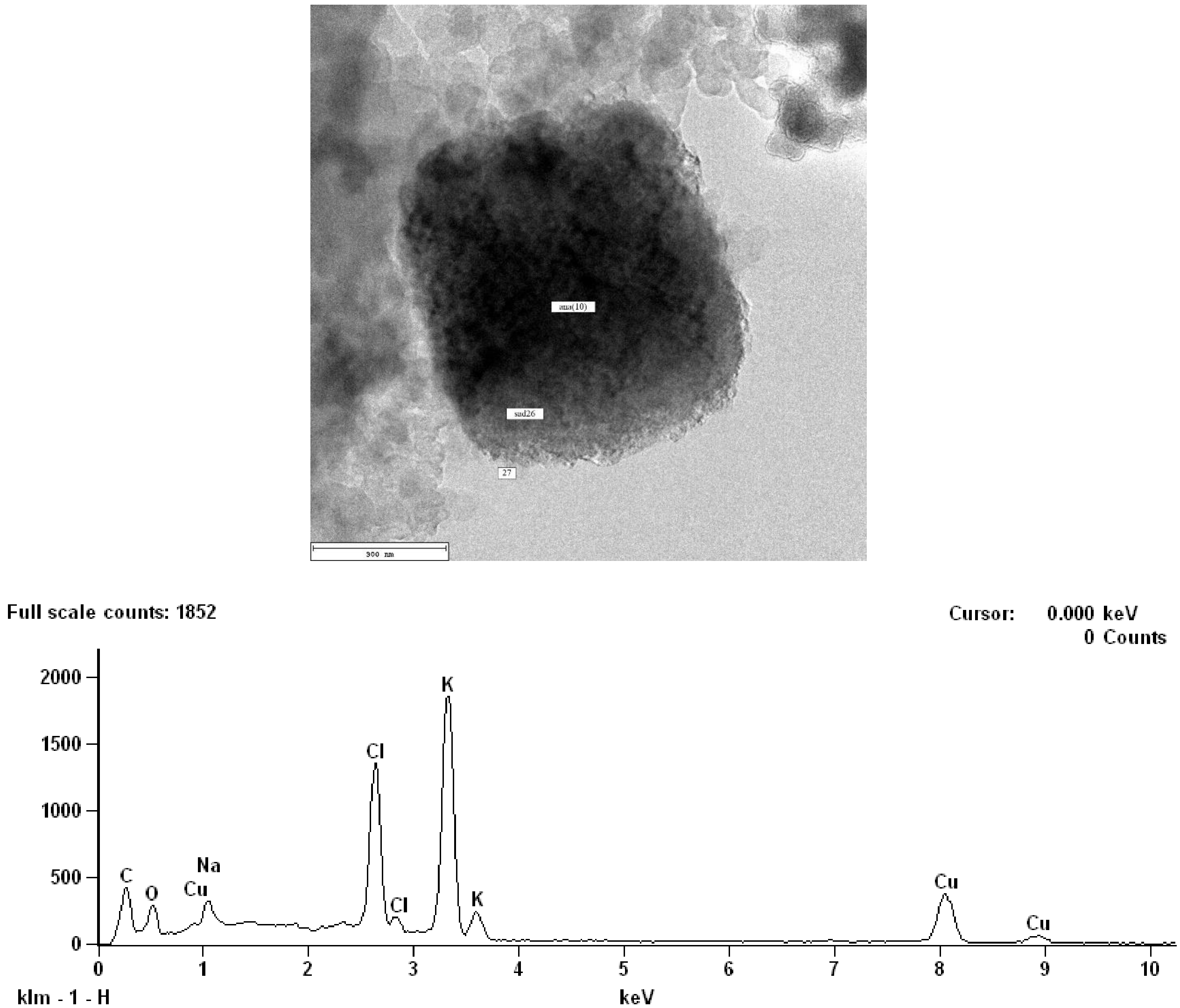

Supplementary Figure S2.8.21: TEM and EDX Analysis of small KCl particles present on electrode post-catalysis

S2.9 Electrochemical data

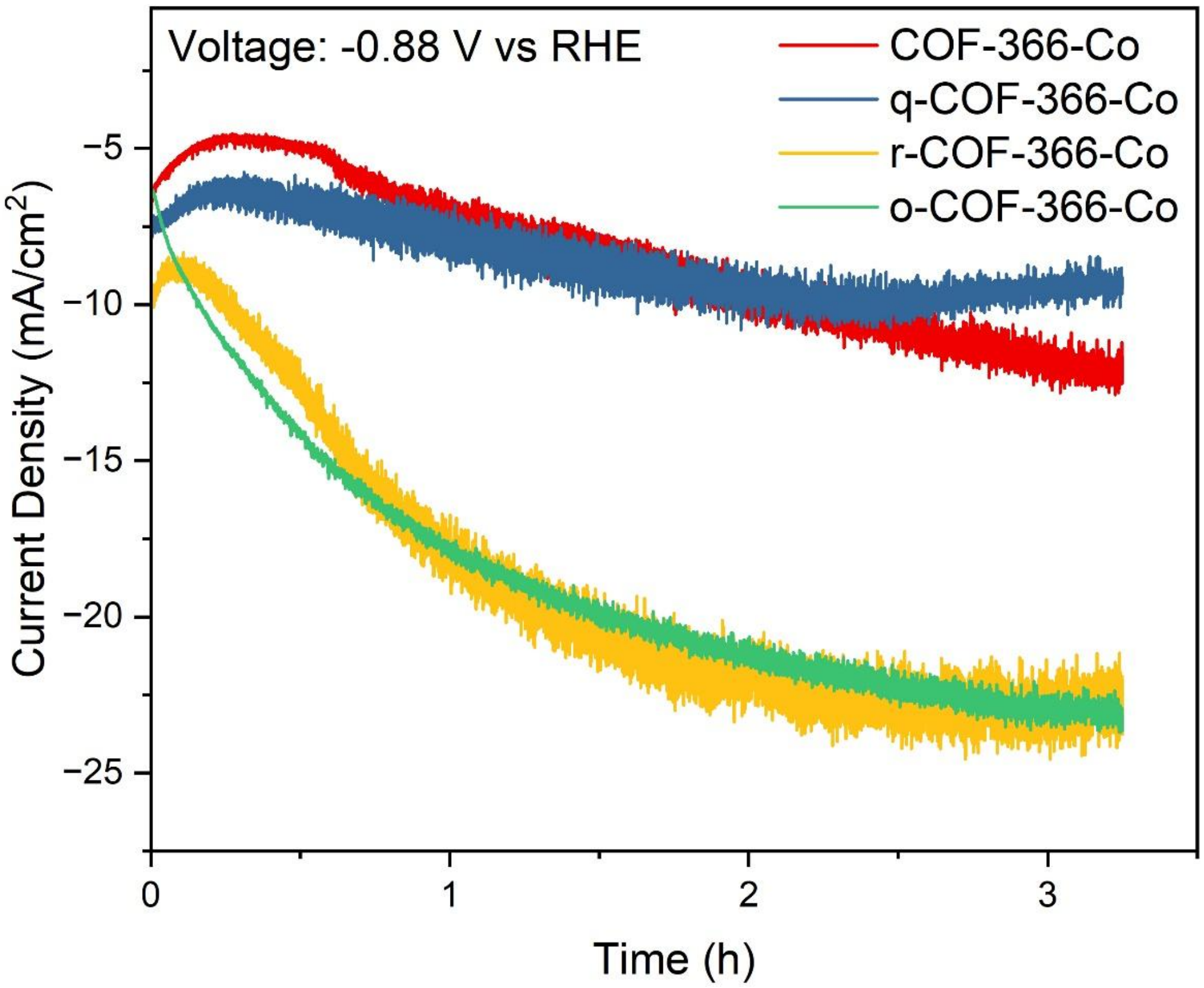

Supplementary Figure S2.9.1: CA of COF-366-Co, as well as functionalized q-, r-, and o-COF-366-Co over a 3-hour period at an applied potential of -0.88V vs RHE.

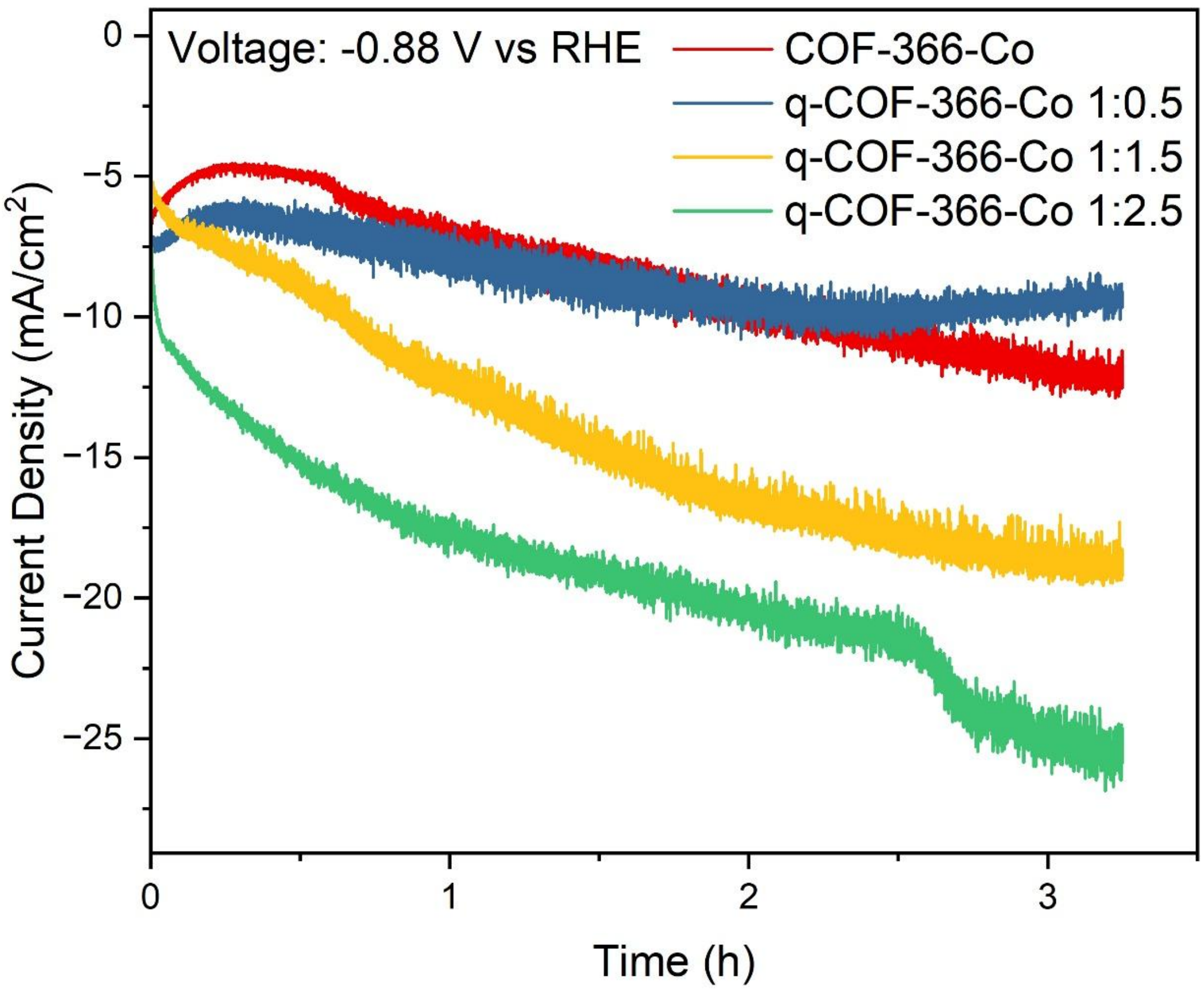

Supplementary Figure S2.9.2: CA of COF-366-Co, as well as functionalized q-COF-366-Co 1:0.5, 1:1.5, and 1:2.5 over a 3 hour period at an applied potential of -0.88V vs RHE.

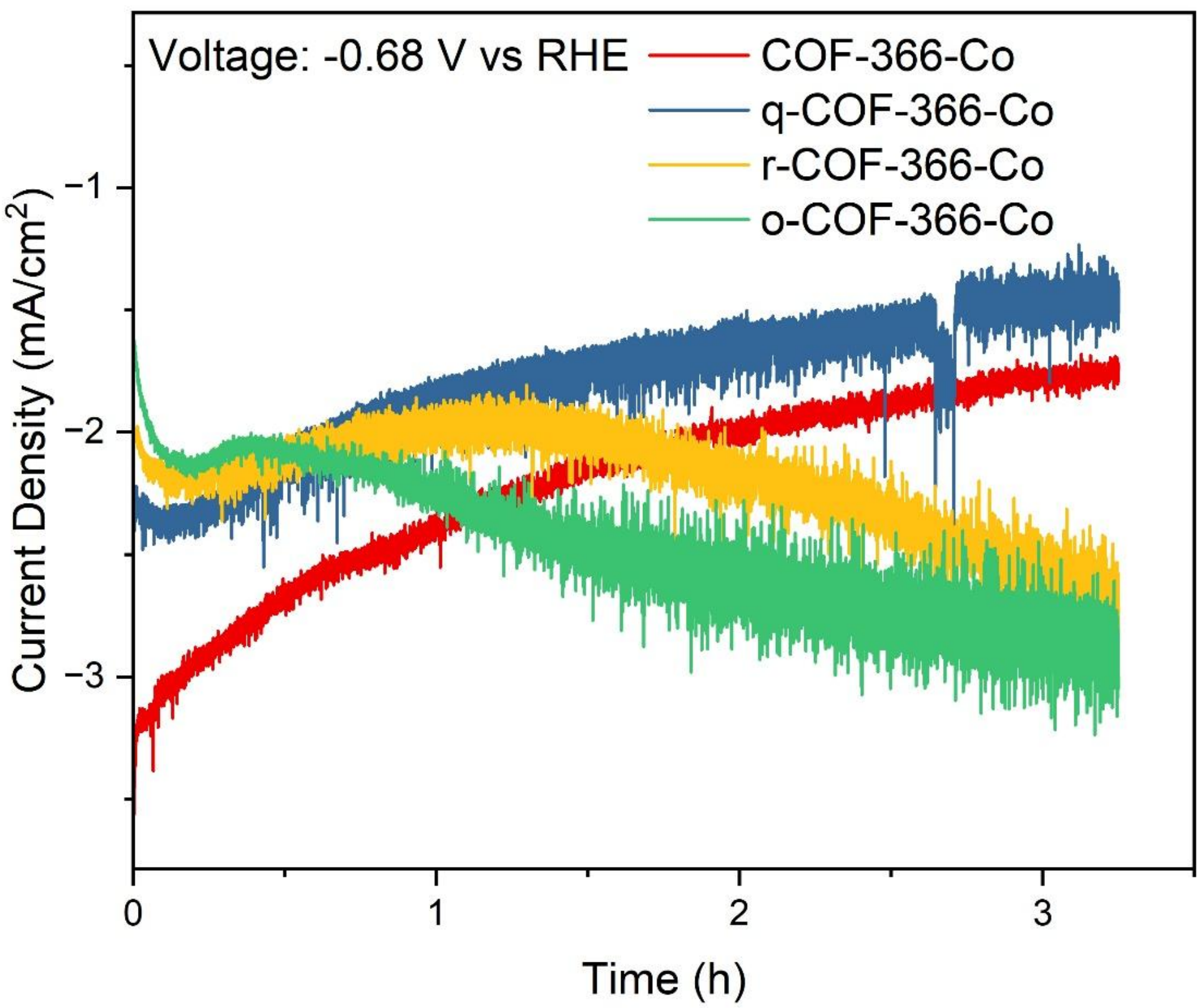

*Supplementary Figure S2.9.3: **CA** of COF-366-Co, as well as functionalized q-, r-, and o-COF-366-Co over a 3 hour period at an applied potential of -0.**6**8V vs RHE.*

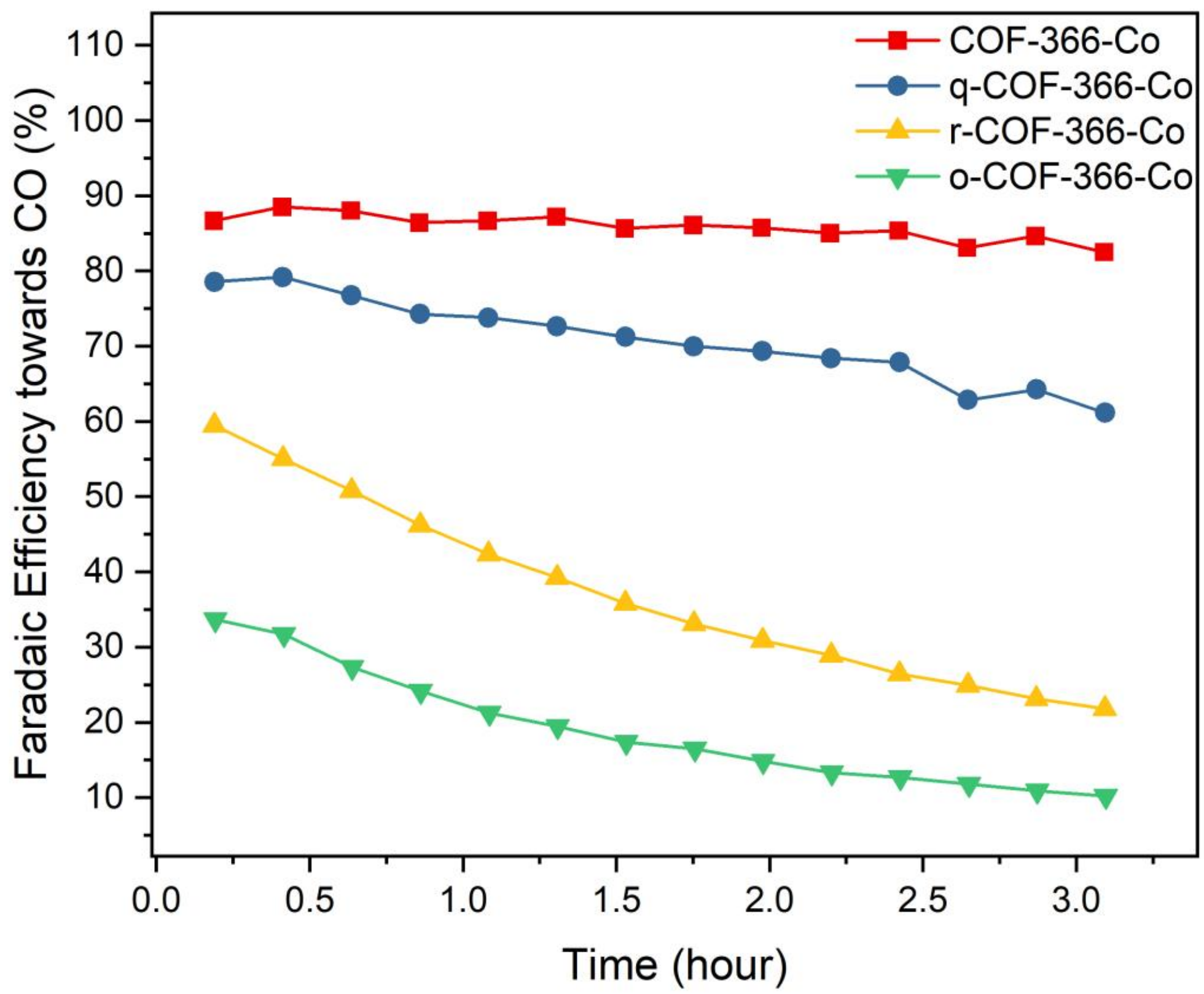

Supplementary Figure S2.9.4: FE toward CO during a 3-hour chronoamperometric measurement at -0.68 V (vs RHE) of COF-366-Co, q-COF-366-Co, r-COF-366-Co, and o-COF-366-Co.

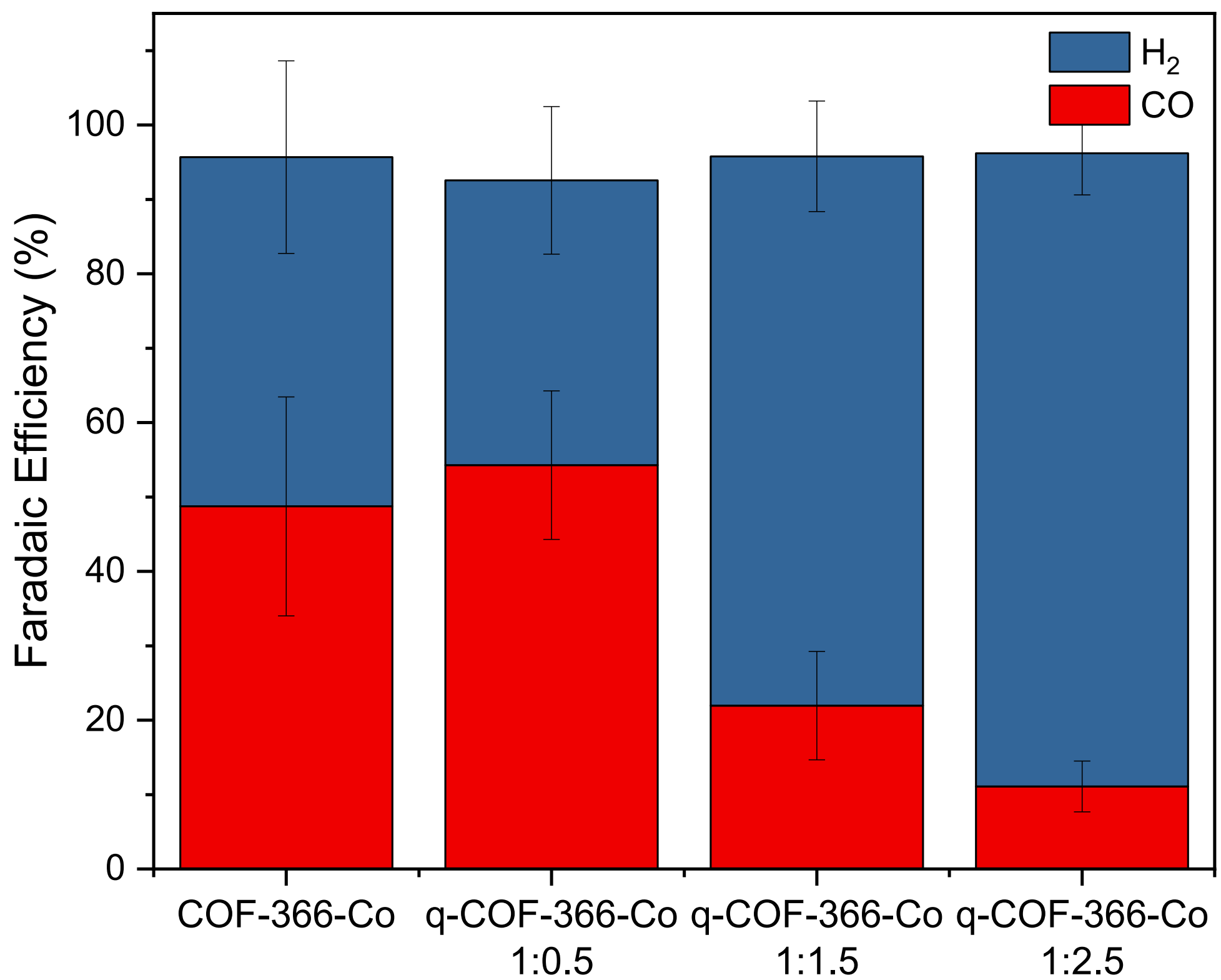

Supplementary Figure S2.9.5: FE of COF-366-Co compared with q-COF-366-Co series at -0.88 V vs RHE.

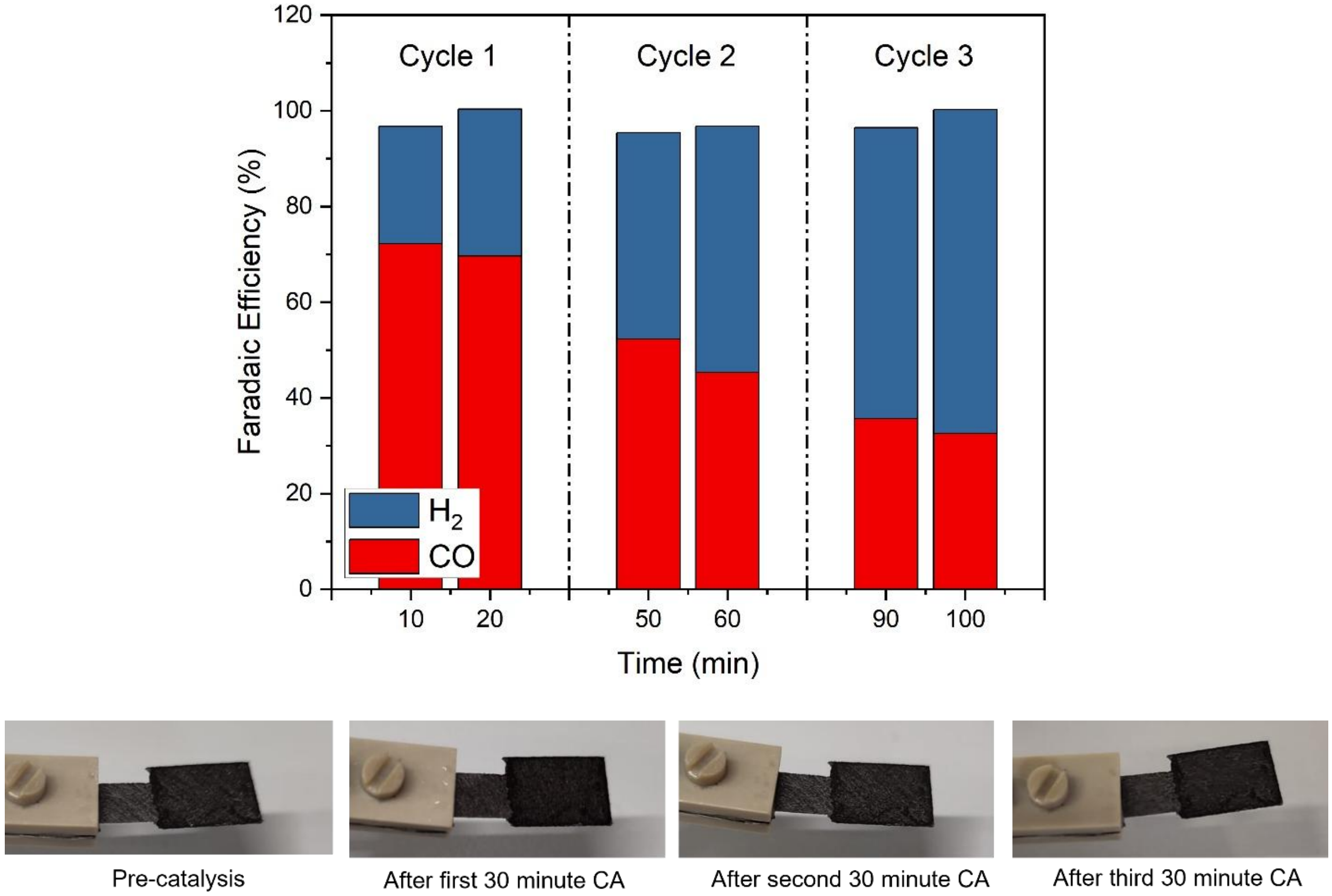

Supplementary Figure S2.9.6: Faradaic Efficiencies of COF-366-Co electrode at -0.88 V vs RHE along 3 consecutive 30-minute CA cycles (top). Pictures of electrode before catalysis and between each CA (bottom).

References

(1) *Contact Angle*. https://imagej.net/ij/plugins/contact-angle.html (accessed 2025-11-17).

(2) Biesinger, M. C. Accessing the Robustness of Adventitious Carbon for Charge Referencing (Correction) Purposes in XPS Analysis: Insights from a Multi-User Facility Data Review. *Applied Surface Science* **2022**, *597*, 153681. https://doi.org/10.1016/j.apsusc.2022.153681.

(3) Fairley, N.; Fernandez, V.; Richard-Plouet, M.; Guillot-Deudon, C.; Walton, J.; Smith, E.; Flahaut, D.; Greiner, M.; Biesinger, M.; Tougaard, S.; Morgan, D.; Baltrusaitis, J. Systematic and Collaborative Approach to Problem Solving Using X-Ray Photoelectron Spectroscopy. *Applied Surface Science Advances* **2021**, *5*, 100112. https://doi.org/10.1016/j.apsadv.2021.100112.

(4) Endo, K.; Raza, A.; Yao, L.; Van Gele, S.; Rodríguez-Camargo, A.; Vignolo-González, H. A.; Grunenberg, L.; Lotsch, B. V. Downsizing Porphyrin Covalent Organic Framework Particles Using Protected Precursors for Electrocatalytic CO2 Reduction. *Advanced Materials* **2024**, *36* (19), 2313197. https://doi.org/10.1002/adma.202313197.

(5) Li, X.; Zhang, C.; Cai, S.; Lei, X.; Altoe, V.; Hong, F.; Urban, J. J.; Ciston, J.; Chan, E. M.; Liu, Y. Facile Transformation of Imine Covalent Organic Frameworks into Ultrastable Crystalline Porous Aromatic Frameworks. *Nat Commun* **2018**, *9* (1), 2998. https://doi.org/10.1038/s41467-018-05462-4.

(6) Grunenberg, L.; Savasci, G.; Terban, M. W.; Duppel, V.; Moudrakovski, I.; Etter, M.; Dinnebier, R. E.; Ochsenfeld, C.; Lotsch, B. V. Amine-Linked Covalent Organic Frameworks as a Platform for Postsynthetic Structure Interconversion and Pore-Wall Modification. *J. Am. Chem. Soc.* **2021**, *143* (9), 3430–3438. https://doi.org/10.1021/jacs.0c12249.
